# Spontaneously charged water drops induce corrosion

**DOI:** 10.1038/s41586-026-10941-6

**Published:** 2026-08-26

**Authors:** Zhongyuan Ni, Xiaomei Li, Aaron D. Ratschow, Lin Jian, Xiaoteng Zhou, Pravash Bista, Diego Cortes, Gunnar Glasser, Haojian Luo, Shuai Chen, Jiyao Yu, Yongkang Wang, Katrin Amann-Winkel, Kaloian Koynov, Rüdiger Berger, Hans-Jürgen Butt

**Affiliations:** 1 Max Planck Institute for Polymer Research, Mainz, Germany; 2 South China Advanced Institute for Soft Matter Science and Technology, School of Emergent Soft Matter, South China University of Technology, Guangzhou, China; 3 Department of Mechanical Engineering, Massachusetts Institute of Technology, Cambridge, MA USA; 4 Institute for Inorganic Chemistry, University of Bonn, Bonn, Germany; 5 Institute of Physics, Johannes Gutenberg University Mainz, Mainz, Germany

**Keywords:** Corrosion, Surfaces, interfaces and thin films, Corrosion, Wetting, Fluids

## Abstract

Water drops spontaneously become electrically charged when moving on different surfaces, such as plant leaves, insect wings, building walls, window glass and plastic^1–9^. This process, known as contact or sliding electrification, is analogous to tribocharging between solids^10–13^. The electric potential of water drops charged in this way can exceed 1 kV (refs. ^14–16^). A vital but as yet unanswered question is whether the charge in water drops causes corrosion. Here we analyse the effect of series of water drops hitting metal surfaces, which are protected by a non-conductive coating. Before hitting the coated metal, the drops acquire a charge spontaneously by sliding over an insulating surface. We demonstrated that these charged drops can cause the coating to break down electrically and lead to corrosion of the metal. As spontaneously charged water drops form naturally, this previously overlooked corrosion mechanism may contribute to the degradation of cultural heritage sites, buildings, ships, cars and other metal components. Our findings can improve anticorrosion strategies and emphasize the need for protective materials capable of resisting charge-induced damage from water drops.

## Abstract (web summary)

Experiments have shown that spontaneously charged water drops can damage coatings on metal surfaces, leading to corrosion of the metal.

## Main

Metal corrosion is a serious economic and safety issue facing modern society^17–19^. Outdoor metal products are particularly vulnerable to water drops from rain, dew, ocean waves and melting snow^20–25^. Using anticorrosion coatings is the simplest and most effective method; however, these coatings inevitably degrade over time^26,27^. Conventional understanding attributes the coating failure induced by water drops mainly to two mechanisms: physical abrasion caused by moving drops^28–30^ and chemical degradation from acidic substances and pollutants in the drops^31–33^.

In recent years, it has been demonstrated that water drops moving over insulating solid surfaces can spontaneously acquire a charge and deposit an opposite charge to the surface^1–9^. Hereby the electric potential of charged water drops can reach several thousand volts^14–16^, depending on the specific materials involved and the thickness of the insulating layer. Here we show a previously overlooked corrosion mechanism induced by water drops: spontaneously charged water drops, whether hitting or sliding, can cause strong electric fields, leading to dielectric breakdown and cause degradation of the protective coating (polymer or oxide layer), thereby allowing subsequent corrosion of the metal substrate. Typically, ‘degradation’ is more commonly used to describe the damage to coatings. However, according to the International Union of Pure and Applied Chemistry (IUPAC), ‘corrosion’ also refers to materials other than metals, such as ceramics and polymers^34^. In the following discussion, we use ‘corrosion’ to refer to the damage of both coatings and metals.

## Spontaneously charged water drops induce corrosion

**Fig. 1:**
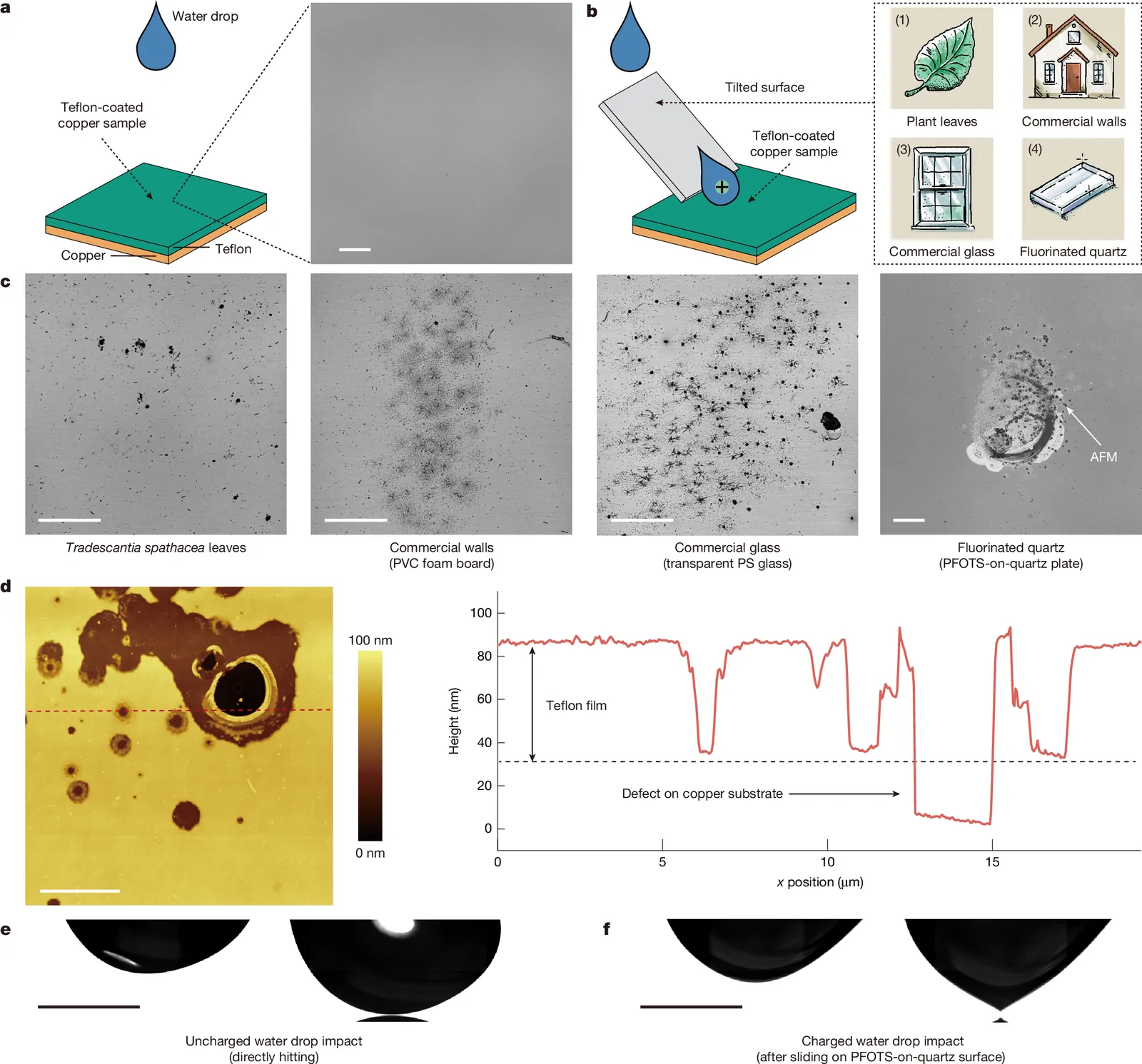
Corrosion induced by spontaneously charged water drops. **a**, Schematic of a water drop directly hitting a 60-nm-thick Teflon film on copper. Reflection mode confocal microscopy (LSM 880, Carl Zeiss) image of the surface morphology after 3,000 water drops. The image was recorded in reflection mode with an Ar-ion laser at 488 nm. The surface morphology of the impact area remains flat and smooth. **b**, Schematic diagram of a water drop sliding on a tilted surface to acquire charge and then hitting a Teflon-coated copper sample. As surface we used plant leaves (1), commercial walls (2), commercial glass (3) or fluorinated quartz (4). **c**, Reflection mode confocal microscopy images of the surface morphology of the Teflon-coated copper sample after impact of 3,000 charged water drops. In contrast to the direct impact of neutral drops, every image shows corrosion of the sample surface. Different images represent different tilted surfaces used to charge water drops. From left to right: *Tradescantia spathacea* leaves, PVC foam board, transparent PS glass and lab-prepared fluorinated quartz (PFOTS coated on quartz plate). **d**, AFM (Dimension Icon, Bruker) image of the surface morphology for the fourth image in **c**. We plotted a line profile along the dashed red line. The corrosion pattern corresponds to hole-like defects. The depth of the defect is in the range of several nanometres and even exceeds the Teflon film thickness into the underlying copper substrate. **e**,**f**, Side-view high-speed camera images of uncharged and charged water drops before hitting the Teflon-coated copper. The bottom of the uncharged water drops from direct impact experiments always retains a smooth arc shape. The bottom of charged water drops forms a cone shape when approaching the Teflon-coated copper surface. Scale bars, 100 μm (**a**,**c**); 5 μm (**d**); 1 mm (**e**,**f**). Source data

As a reference, we studied electrically neutral water drops directly falling onto a sample (Fig. 1a). 35-μl water drops were continuously released at 12-s intervals from a height of 4 cm and directly dropped onto a Teflon-coated copper sample. Usually, water drops in nature, such as rainwater, are not distilled water but contain some salt, whose concentration depends on local environmental conditions^35,36^. In our experiments, the water drops contained 1 mM NaCl. We made the sample by sputtering a 35-nm-thick layer of copper onto a smooth quartz plate. Then a 60-nm-thick Teflon film was deposited by dip-coating onto the copper substrate from a 1-wt% Teflon AF 1600 solution. Finally, the samples were annealed at 160 °C under vacuum for 24 h. We chose the Teflon coating because it is chemically robust. The samples were tilted by 10° so that water drops ran off before the subsequent drops hit the surface. We analysed the sample surface by reflection mode confocal microscopy after 3,000 water drops had directly hit it. This analysis revealed flat and smooth surfaces. There was no notable difference in the morphology of the sample surface before or after the drop hit it.

**Extended Data Fig. 1:**
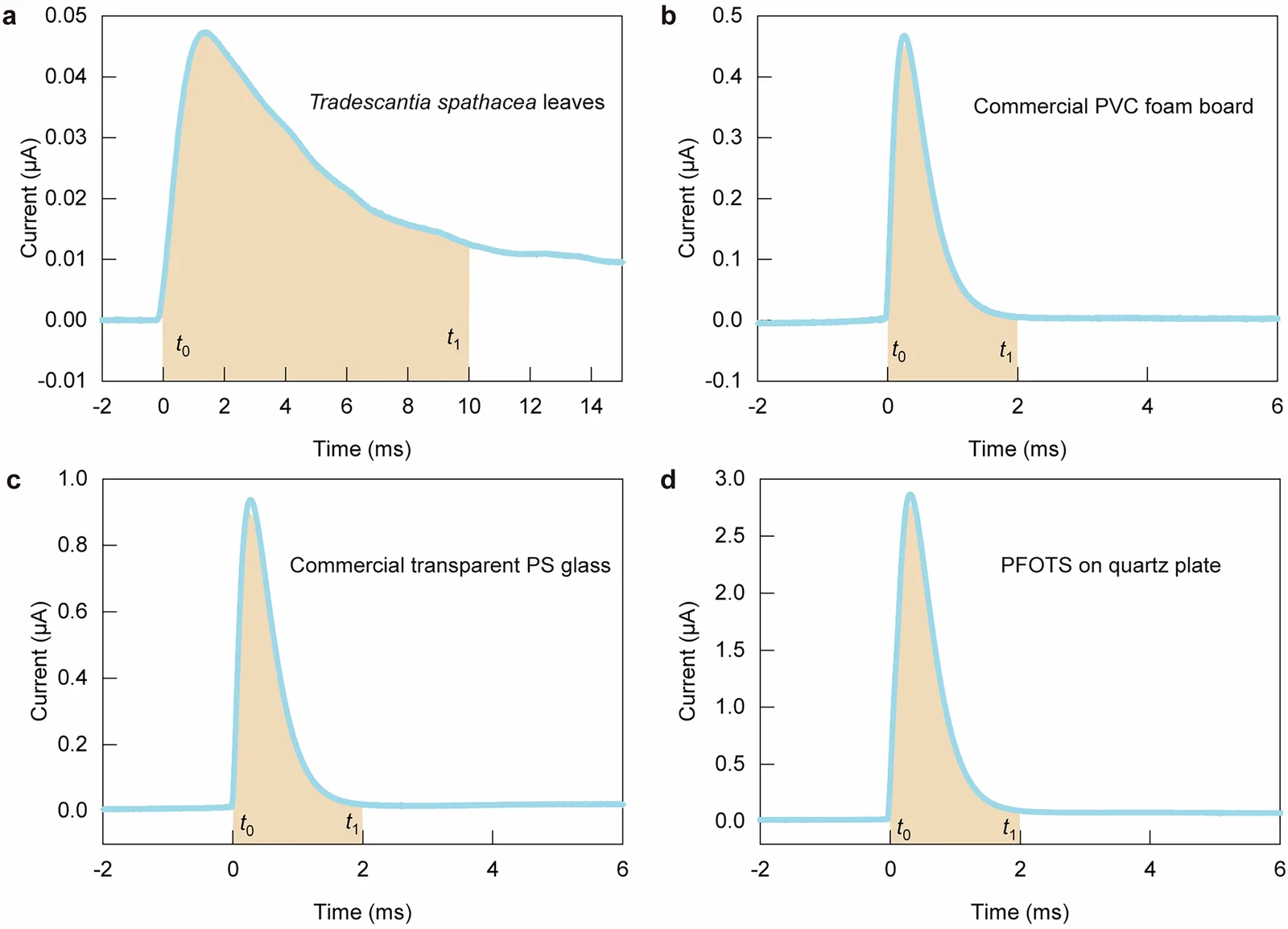
Slide electrification of water drops on various tilted surfaces. Water drops slide down about 4 cm on the surface at a tilt angle of 50°. Then the front part of the water drop touches the tungsten wire electrode of the current amplifier. Panels **a**–**d** show the measured current signals, *I*, for different tilted surfaces. We calculated the amount of charge transferred by integrating the current signal (${\int }_{{t}_{0}}^{{t}_{1}}I{\rm{d}}t$). For *Tradescantia spathacea*, PVC, PS and PFOTS, we registered charges of 0.2, 0.3, 0.7 and 2 nC, respectively. Source data

To demonstrate that the charge caused by slide electrification in water drops induces corrosion, while keeping other conditions unchanged, we let water drops slide down four different 50° tilted surfaces before they fell onto the Teflon-coated copper sample. We selected four surfaces that are representative of many surfaces we encounter every day (Fig. 1b). A plant leaf: *Tradescantia spathacea* leaves (Fig. 1b (1)). A construction material: polyvinyl chloride (PVC) foam boards of 3 mm thickness (Fig. 1b (2)). A window: polystyrene (PS) glass of 2 mm thickness (Fig. 1b (3)). PVC and PS were purchased from a local hardware store. A widely used hydrophobic surface coating: perfluorooctadecyltrichlorosilane (PFOTS) coated on quartz (Fig. 1b (4)). The PFOTS was prepared on a 1-mm-thick quartz plate by chemical vapour deposition (CVD). Depending on the specific tilted surface, water drops acquired a charge between 0.2 and 2 nC after sliding for 4 cm (Extended Data Fig. 1). Then the water drops slid off the tilted surfaces and hit the Teflon-coated copper sample, dropping from a height of 5 mm. The reflection mode confocal microscopy images showed corrosion patterns in all four cases after the impact of 3,000 water drops (Fig. 1c). The corrosion pattern was located in the area of the drops’ initial contact with the sample surface. Atomic force microscopy (AFM) revealed that the charged water drop led to the hole-like defects on the sample surface (Fig. 1d). Both the Teflon film and the underlying copper substrate were corroded.

**Extended Data Fig. 2:**
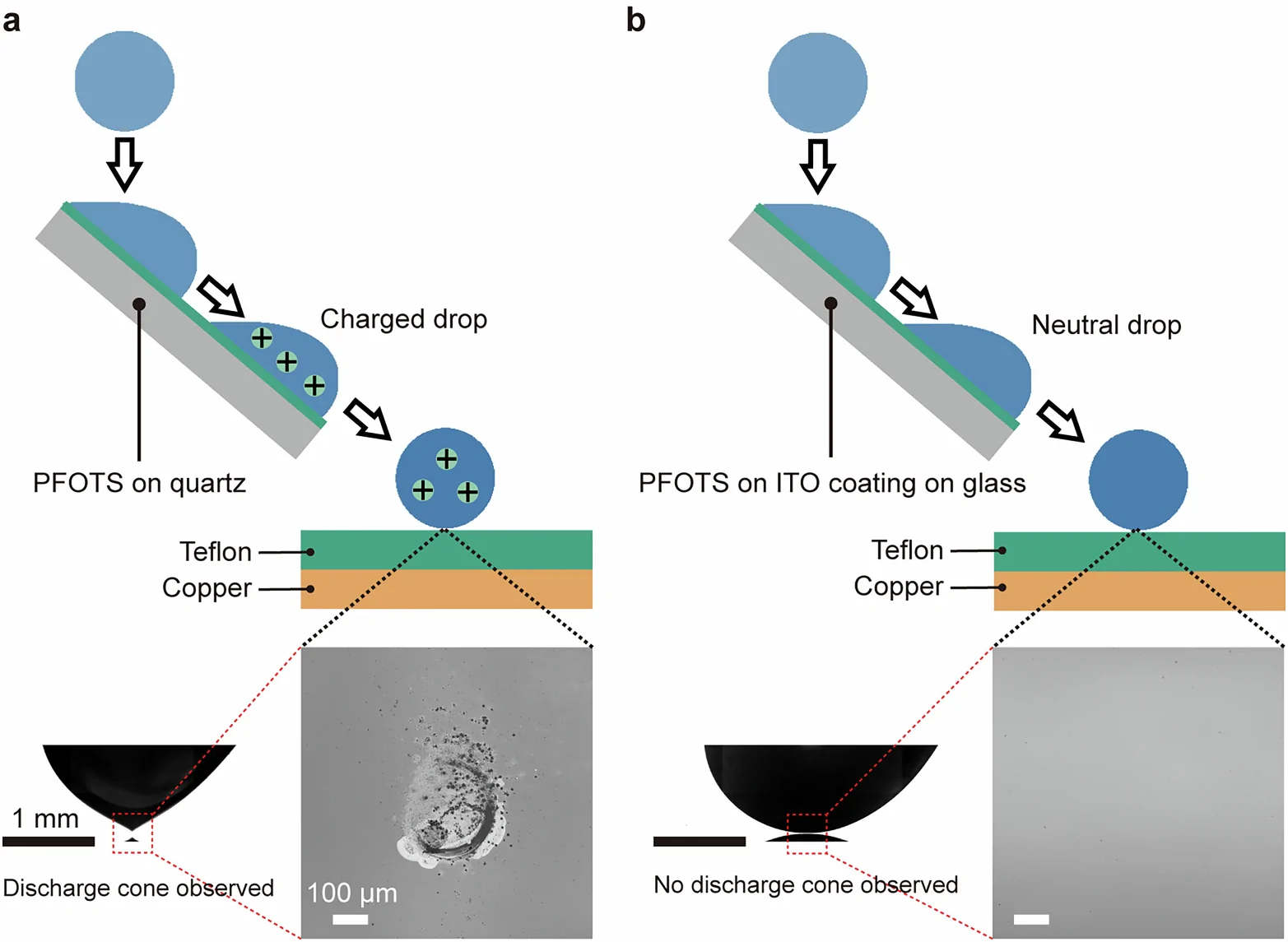
Electrostatic cone formation before drop impact for charged and neutral water drops. The dielectric properties of the substrate affect the slide electrification effect. Quartz is insulating and ITO is electrically conductive. The drop (35 µl, 1 mM NaCl) sliding over PFOTS-coated quartz formed a discharge cone when approaching the Teflon-coated copper, whereas the drop sliding over PFOTS on ITO coating on glass did not. After 3,000 drops had hit, obvious surface damage or corrosion in the drops’ initial contact area with the sample surface was observed when drops slid by PFOTS on quartz, whereas the surface remained intact when drops slid by PFOTS on ITO glass.

**Extended Data Fig. 3:**
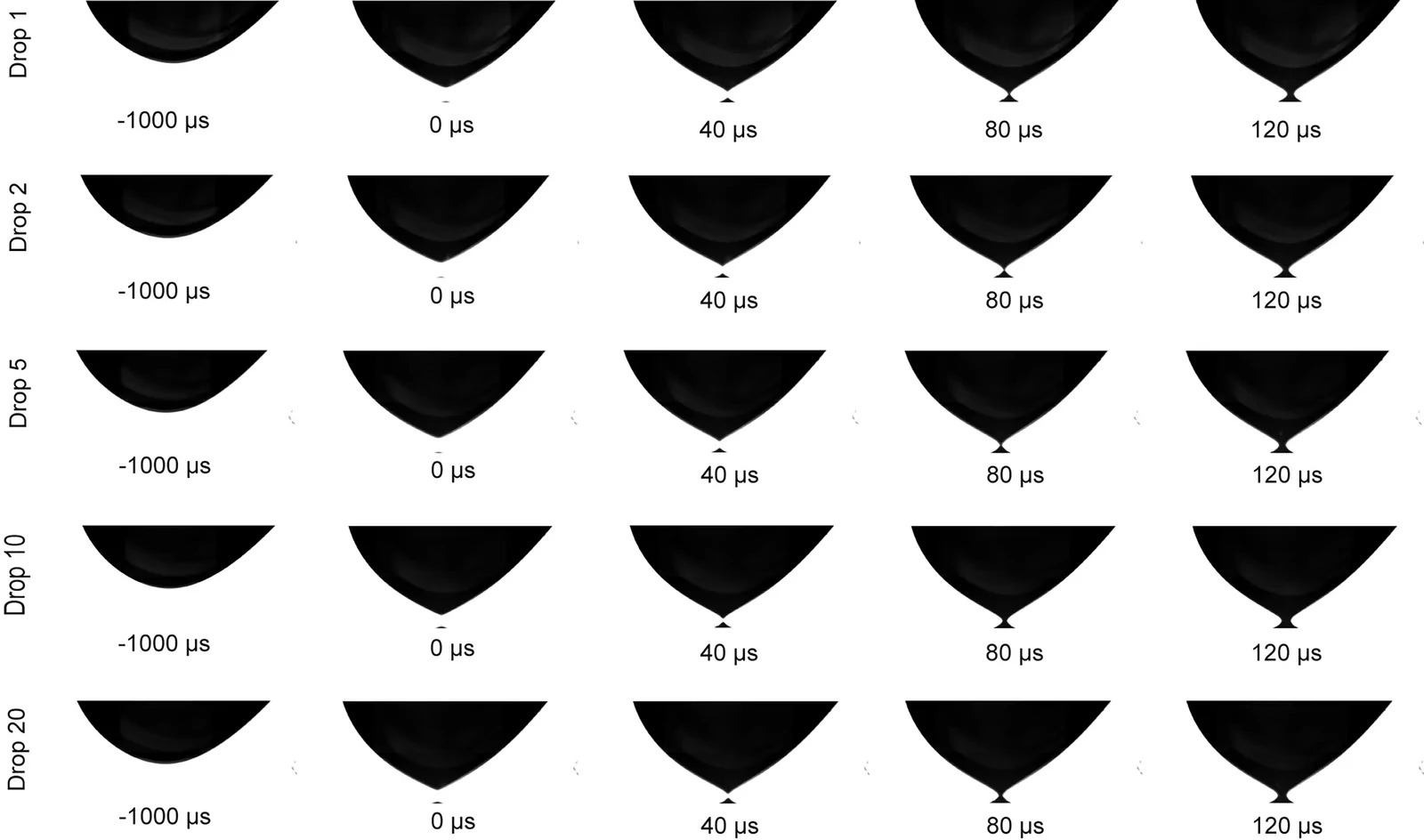
High-speed video images of charged water drops as they approach a Teflon-coated copper surface. Series of images for drops 1, 2, 5, 10 and 20 that hit a Teflon-coated copper surface (horizontal rows). Each water drop always showed the same behaviour. When it approached the surface, the bottom of the water drop stretched downwards, pulling the surrounding liquid to form a cone-like structure. Then the drop touched the surface (40 µs to 80 µs) and shot onto the sample surface. By contrast, electrically neutral drops showed no cone-like deformation before impact (Supplementary Fig. 19).

To gain better insight into the cause of corrosion, water drops hitting the Teflon-coated copper sample were imaged from the side with a high-speed camera. Electrically neutral drops showed no sign of deformation in the drop shape before contact with the sample surface; these drops either came out of a grounded metal needle (Fig. 1e and Supplementary Video 1) or had slid over a grounded PFOTS layer on indium tin oxide (ITO) (Extended Data Fig. 2). By contrast, when water drops charged by sliding over a PFOTS-on-quartz surface and then came close to the sample surface, the bottom of the drop elongated and formed a cone shape (Fig. 1f, Extended Data Fig. 3 and Supplementary Video 2). This behaviour is similar to the formation of a Taylor cone in the electrospraying process, in which the electrostatic force acting on the liquid surface overcomes the surface tension, thus causing deformation^37,38^. We attribute this cone formation of the drop to the strong electric field generated when approaching the sample, which typically serves as an indicator of sample surface corrosion.

**Extended Data Fig. 4:**
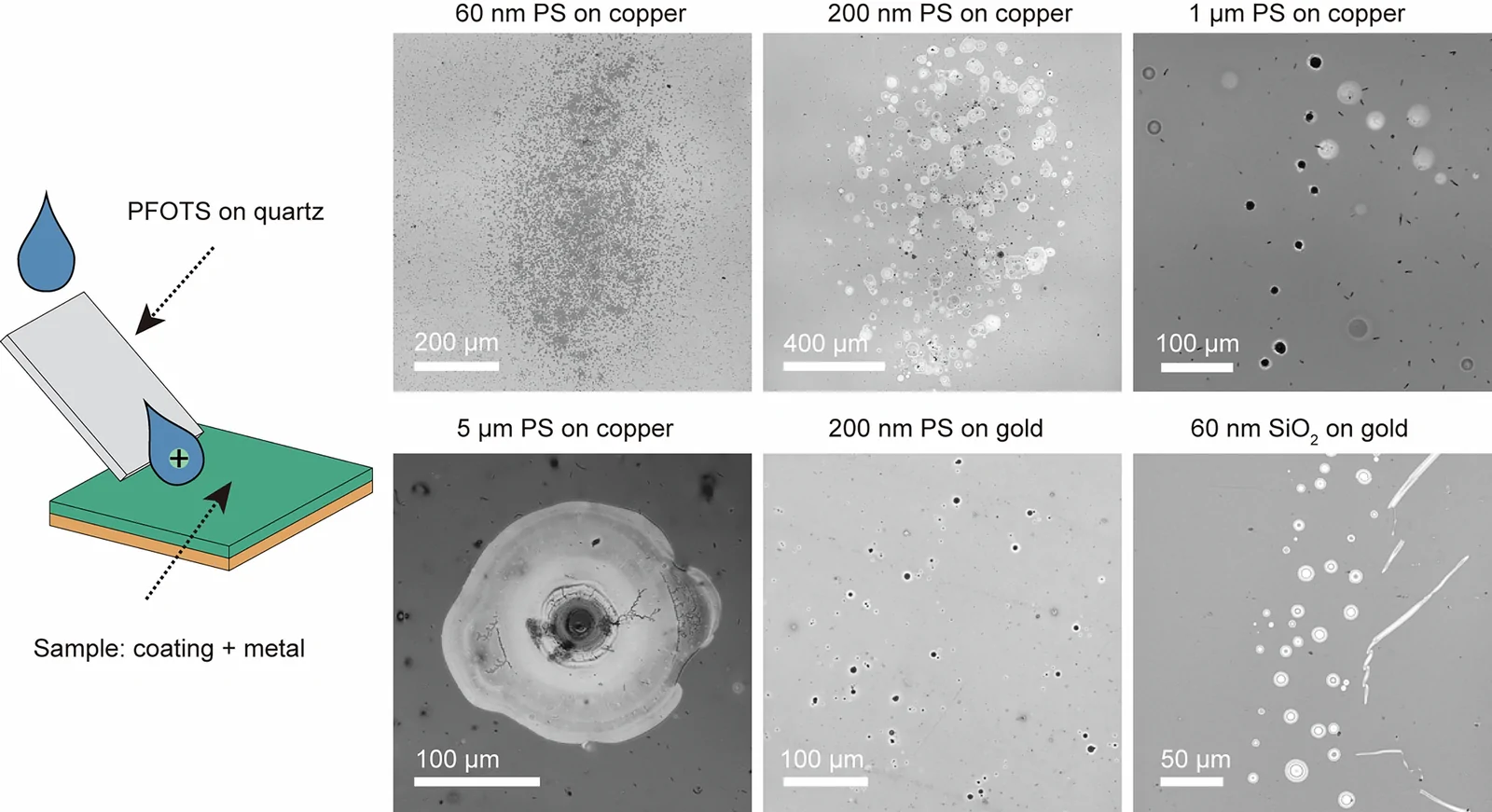
Corrosion of metal substrates protected by a coating. We used a tilted surface made of PFOTS on quartz for slide electrification of water drops. The samples that were hit by 3,000 charged water drops are: 60-nm PS-coated copper, 200-nm PS-coated copper, 1-μm PS-coated copper, 5-μm PS-coated copper, 200-nm PS-coated gold and 60-nm SiO_2_-coated copper. Corrosion was observed in all samples.

To demonstrate that the charge-induced corrosion is not restricted to Teflon-coated copper surfaces, PS films (192 kg mol^−1^) of thickness 60 nm, 200 nm, 1 μm and 5 μm were prepared on copper, as well as a 200-nm-thick PS film on sputtered gold by dip-coating and a 60-nm-thick SiO_2_ sputter-coated film on gold. Surface corrosion similar to that shown in Fig. 1c was observed at the initial contact area after 3,000 charged drops had hit (Extended Data Fig. 4). We conclude that corrosion induced by spontaneously charged water drops on metals coated with an insulating layer is a general phenomenon.

## Water drop discharge

**Fig. 2:**
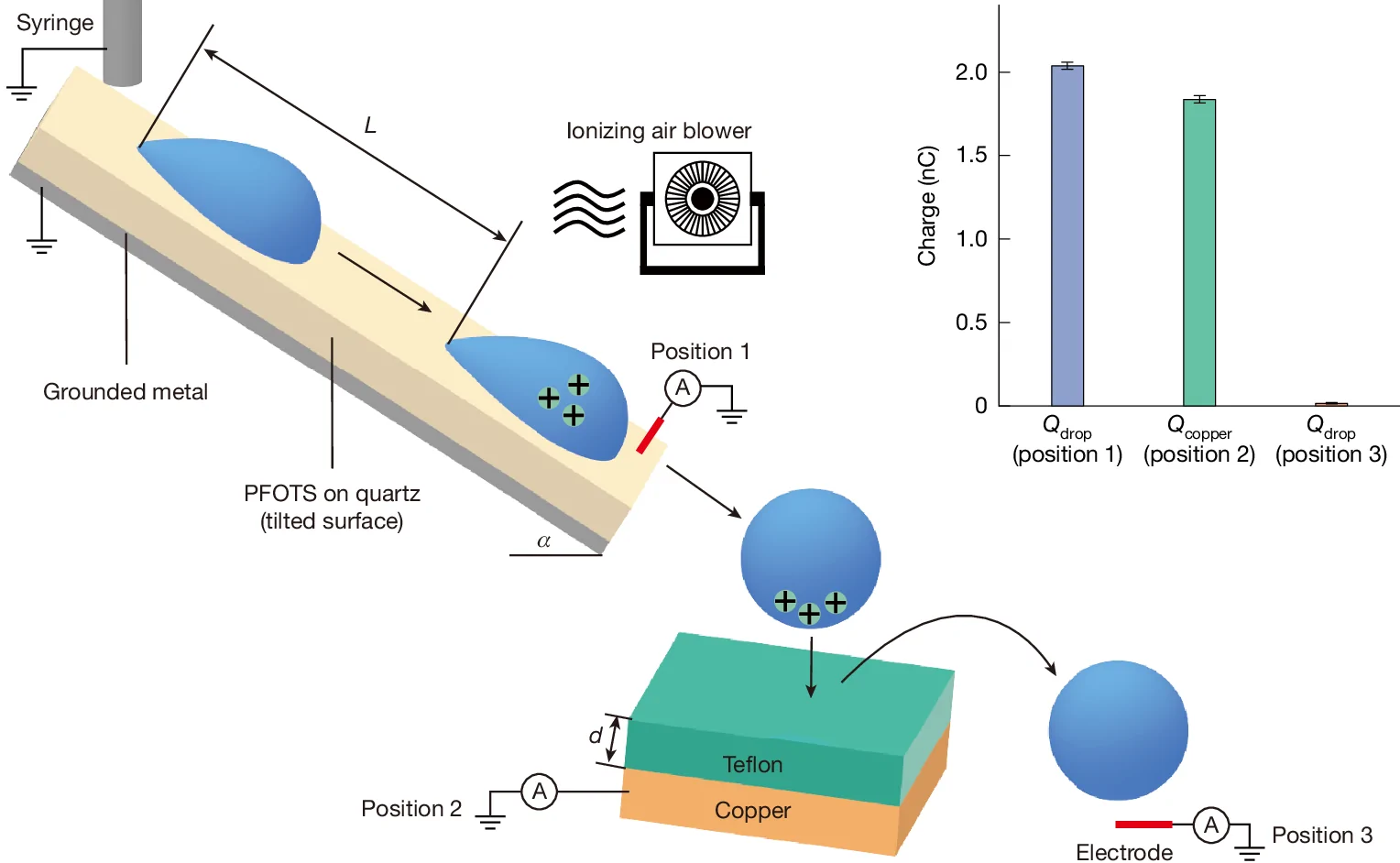
Charge measurement. Schematic of the charge measurement. Water drops fall from the syringe tip onto a tilted surface (*α* = 50°), here a PFOTS on quartz. Drops slide down a length *L* = 4 cm to reach the edge and then fall onto a Teflon-coated copper sample, which is slightly tilted to enable drop roll-off. A current amplifier (response time: 0.8 ms, FEMTO DDPCA-300), placed at three distinct positions, was used to monitor the respective charge changes. In position 1, a tungsten wire (0.7 mm in diameter) was used, which acts as an electrode connected to the current amplifier. Here a charge of +2.0 ± 0.021 nC (inset blue bar) generated by drop slide electrification is detected when the drop makes contact with the tungsten wire. In position 2, the copper substrate is directly connected to the current amplifier. In this case, we detected the charge transfer of +1.8 ± 0.022 nC (inset green bar) when the drop hits the sample. In position 3, the drop rolls off the sample and then makes contact with the tungsten electrode. Almost no charge (+0.016 ± 0.003 nC) is detected remaining in the drop (inset orange bar). The set-up is purged continuously with an ionizing air blower (Aerostat PC ionizing air blower, Simco-Ion) to neutralize the PFOTS surface between two subsequent drops to ensure that each water drop has the same slide electrification effect on the PFOTS on quartz. The bar chart shows the average and standard deviation of 20 measurements of drop charge obtained at three positions. Source data

**Extended Data Fig. 5:**
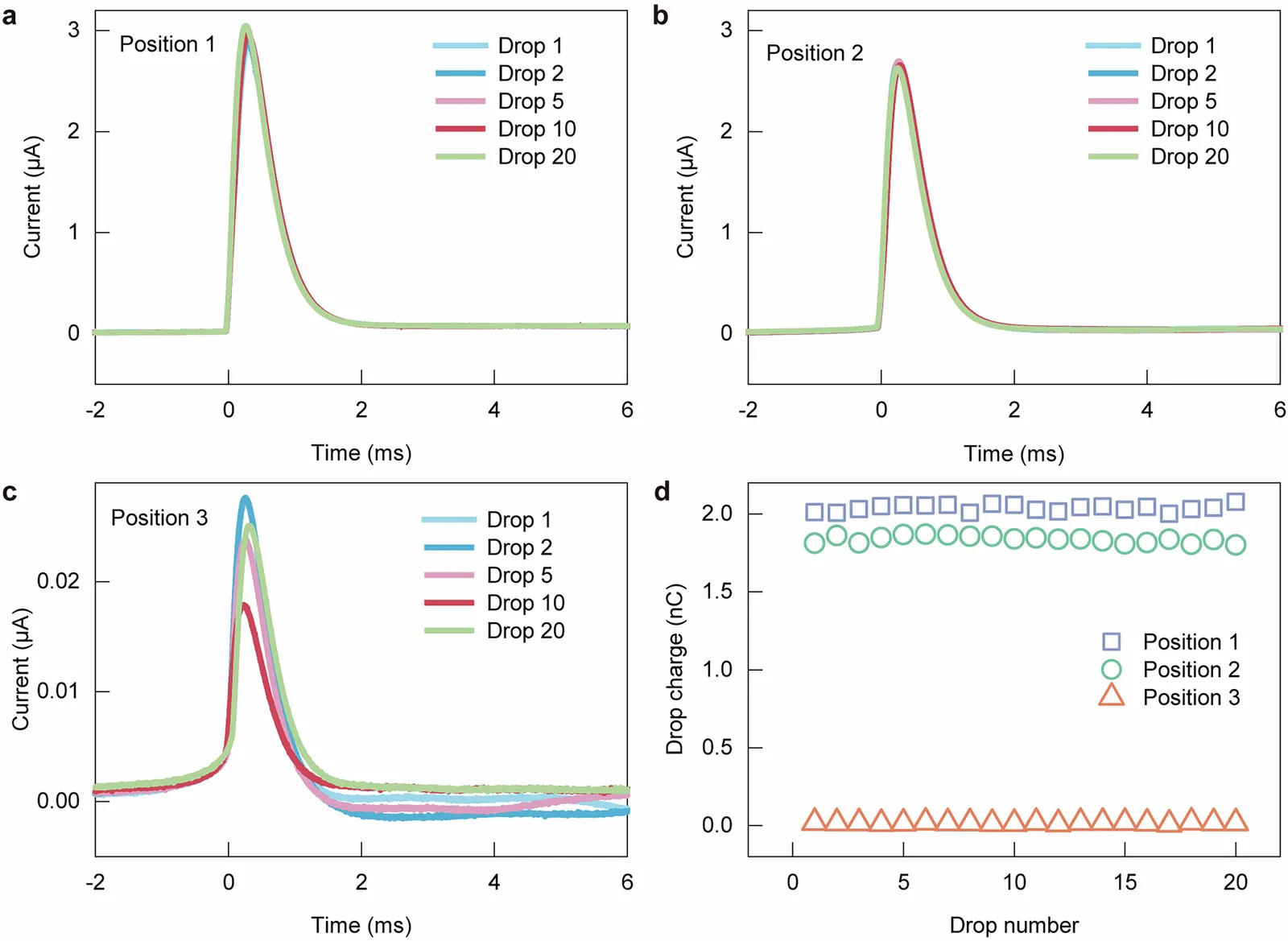
Current measurements at different positions. **a**–**c**, The current (*I*) of a drop was measured 20 times at the end of the sliding process (**a**), at the electrode where the drops hit (**b**) and after sliding of the sample (**c**). Note that the current is reduced by a factor of approximately 100 in **c**. **d**, We calculated the amount of charge by integrating the current signal (${\int }_{{t}_{0}}^{{t}_{1}}I{\rm{d}}t$, *t*_0_ = 0, *t*_1_ = 2 ms). Source data

Given that drop cone formation is strongly correlated with discharge phenomena, we placed a current amplifier at three distinct positions and monitored the respective charge changes (Fig. 2). A drop sliding 4 cm down a tilted PFOTS-on-quartz surface acquired a charge of +2.0 ± 0.021 nC (position 1, Fig. 2 inset blue bar and Extended Data Fig. 5a). When the drop hit the 60-nm Teflon-on-copper surface, most of the charge was transferred into the copper layer, +1.8 ± 0.022 nC, (position 2, Fig. 2 inset green bar and Extended Data Fig. 5b). At position 3, after leaving the Teflon-on-copper surface, the drop retained only +0.016 ± 0.003 nC (Fig. 2 inset orange bar and Extended Data Fig. 5c). These results indicate that the highest charge is exchanged between the drop and copper substrate at the moment of impact. We conclude that corrosion of the sample is caused by the discharge of water drops.

## Water drop discharge model

**Fig. 3:**
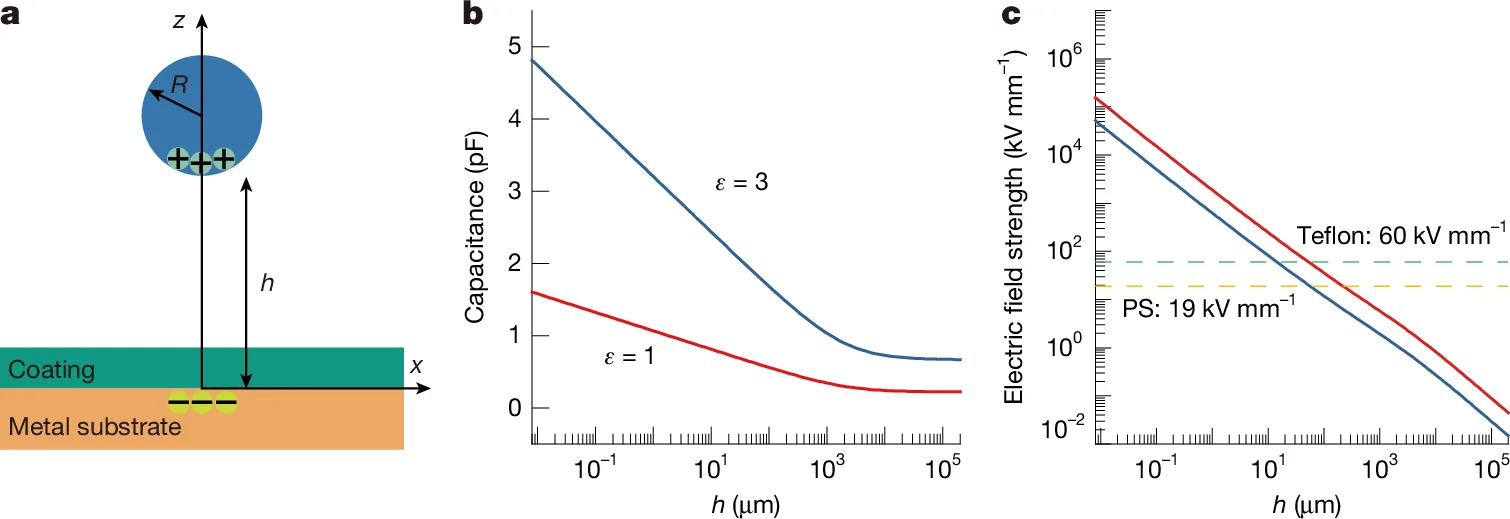
Mechanism of water drop discharge. **a**, Schematic of a charged water drop over a metal substrate. **b**, Capacitance of a water drop *C* versus the distance *h* calculated for air (*ε* = 1) and a typical polymer (*ε* = 3). The true capacitance is described by *ε* = 1 for large distances (main medium is air) and *ε* = 3 for short distances (main medium is a polymer). **c**, Electric field strength between the charged water drop and the copper substrate versus distance. A water drop containing a charge of 2 nC can cause dielectric breakdown at a distance of about 10 μm for a Teflon-coated (dielectric strength, 60 kV mm^−1^) or about 50 μm for a PS-coated (dielectric strength, 19 kV mm^−1^) metal substrate.

**Extended Data Fig. 6:**
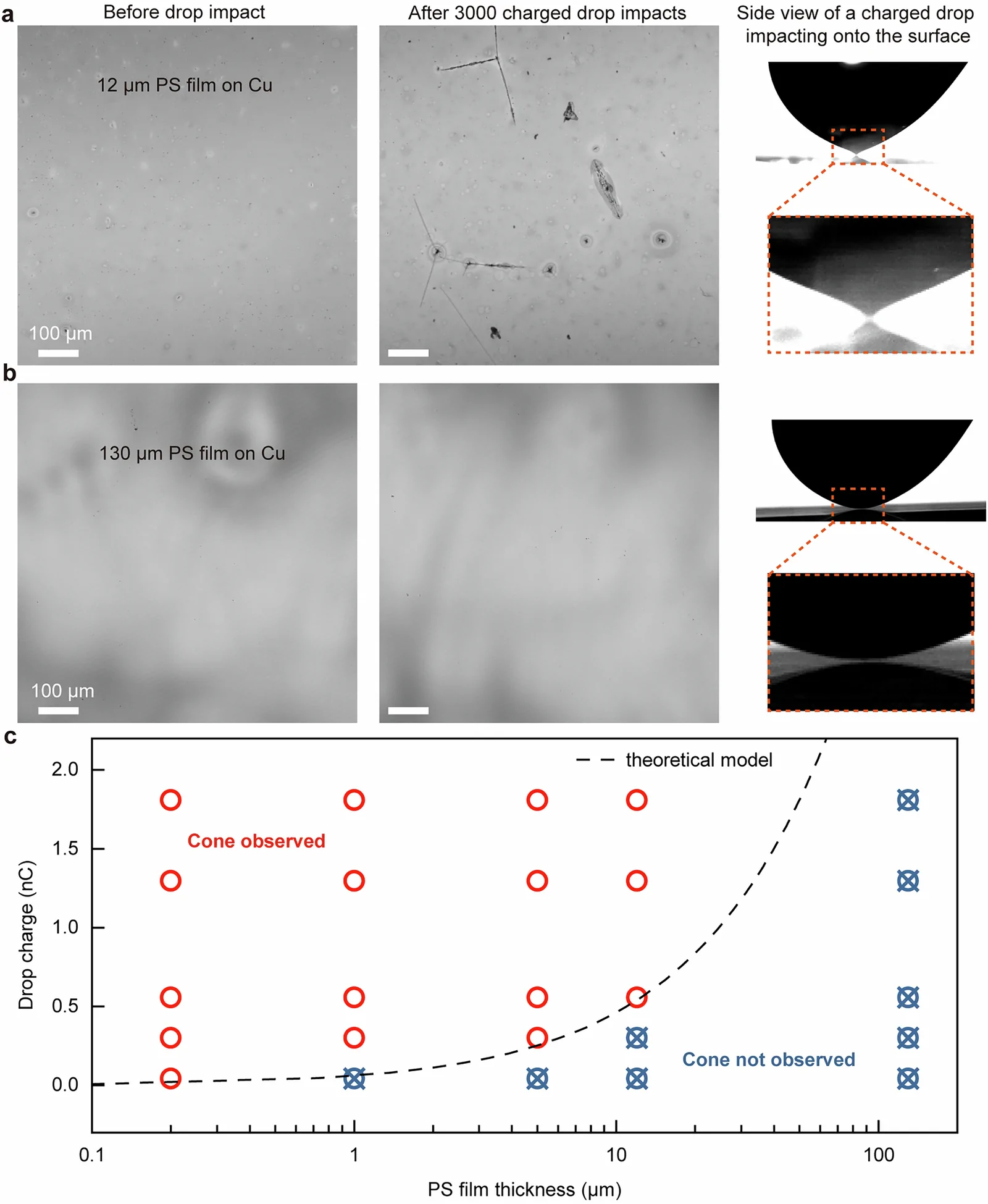
Charged-drops impact on different thicknesses of PS film on Cu. Water drops (35 µl, 1 mM NaCl) were charged by sliding down commercial FEP films (about 1 nC; Supplementary Fig. 4) and then they hit PS-coated Cu samples. **a**,**b**, Reflection confocal microscopy images of the surface morphology of a 12-μm-thick (**a**) and a 130-μm-thick (**b**) PS film. The blurred feature arose from a reflection at the film bottom. On the left are images before and in the middle are images after 3,000 charged drops had hit the PS films on Cu. On the right are images of a side-view high-speed camera video of a single charged drop hitting the surface. Cone formation of the drop serves as an indicator of sample surface corrosion. **c**, Phase diagram of cone formation for different drop charges and PS film thicknesses. Red circles indicate that a cone was observed and blue crossed circles indicate that no cone was observed. Dashed black line represents the threshold calculated from the model in Fig. 3. Source data

**Extended Data Fig. 7:**
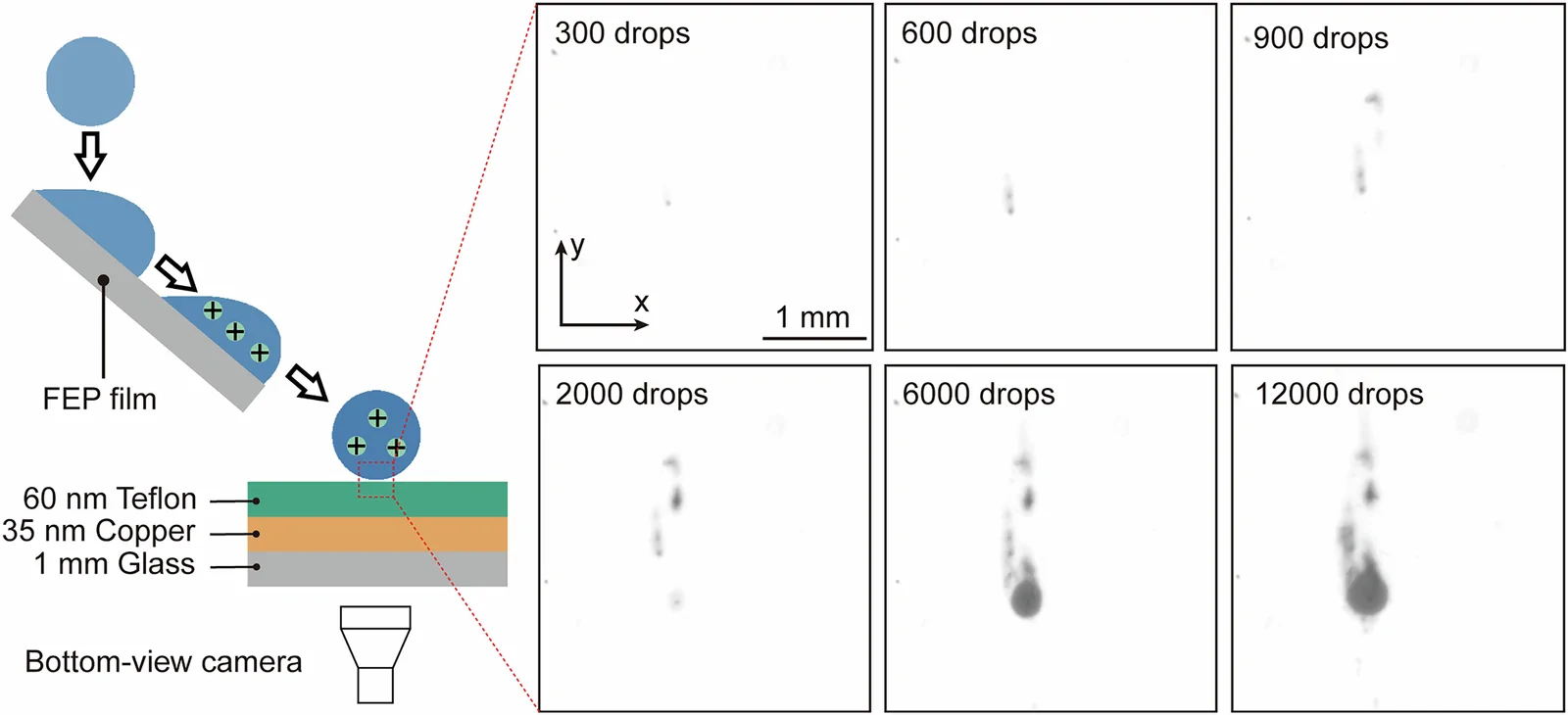
In situ observation of the development of surface damage with increasing number of charged-drop hits. Water drops (35 µl, 1 mM NaCl) charged by sliding down a commercial FEP film (about 1 nC). We used a bottom-view camera (not high speed, FLIR Blackfly S) to observe the initial contact region on the surface during drop impact. As the number of drops increased, the damaged and corroded area also increased.

We suggest that discharge is caused by dielectric breakdown of the insulating coating when the water drop approaches the sample surface. When the electric field strength between two electrodes exceeds the dielectric strength of the medium, dielectric breakdown occurs. Charge is transferred from one electrode to another through the medium. In our case, the water drop and the metal substrate, respectively, act as two electrodes (Fig. 3a). The water drop accumulates a positive charge through slide electrification. In the metal substrate, an equivalent amount of negative image charge is induced. Together, the positive charge in the water drop and the negative charge in the metal substrate create an electric field. As the drop approaches the surface, the electric field increases. To estimate the electric field during approach, the water drop was assumed to act as a conductive sphere of radius *R* opposite to a conductive wall formed by the metal. Then the capacitance of the water drop changes with distance as *C* = 4π*εε*_0_*R*(1 + 0.5ln(1 + *R*/*h*)) (ref. ^39^). Here, *ε* is the relative permittivity of the intervening medium, *ε*_0_ is the vacuum permittivity and *h* is the distance from the surface of the metal substrate to the bottom of the water drop. In the experiment, *R* is 2.0 mm. When the distance is large, the capacitance should be calculated using the relative permittivity of air; at impact, it is more appropriate to use the relative permittivity of the coating (Fig. 3b). According to *U* = *Q*/*C* (*U*: voltage between the water drop and metal, *Q*: charge of the water drop), the change in electric field strength can be estimated, *E* = *U*/*h*, as the water drop approaches the surface (Fig. 3c). The charged water drop leads to breakdown of up to a film thickness of about 10 μm for Teflon or about 50 μm for PS. This corresponds to dielectric strengths of 60 kV mm^−1^ and 19 kV mm^−1^, respectively. Thus, the calculations demonstrate that a water drop carrying approximately nC charge can break down most micrometre-thick insulating coatings (Extended Data Fig. 6). On the basis of Joule heating effect estimation, the damage caused to the Teflon film by a single charged drop is in the sub-μm range (Supplementary Information Discussion 1). The accumulation of Joule heating-induced localized damages (Extended Data Fig. 7) led to metal exposure and increased corrosion risk.

Our estimation of electric field strength represents a lower limit. Before the water drop touches the sample, it forms a conical structure at the bottom (Fig. 1f). This deformation would lead to a lower effective radius of curvature and thus a locally higher electric field. A more complete analysis of dielectric breakdown needs to take into consideration the coating thickness. Rather than assuming a constant dielectric strength, in reality, the dielectric breakdown strength of a coating is known to depend on the thickness^40^.

## Products of corrosion

**Fig. 4:**
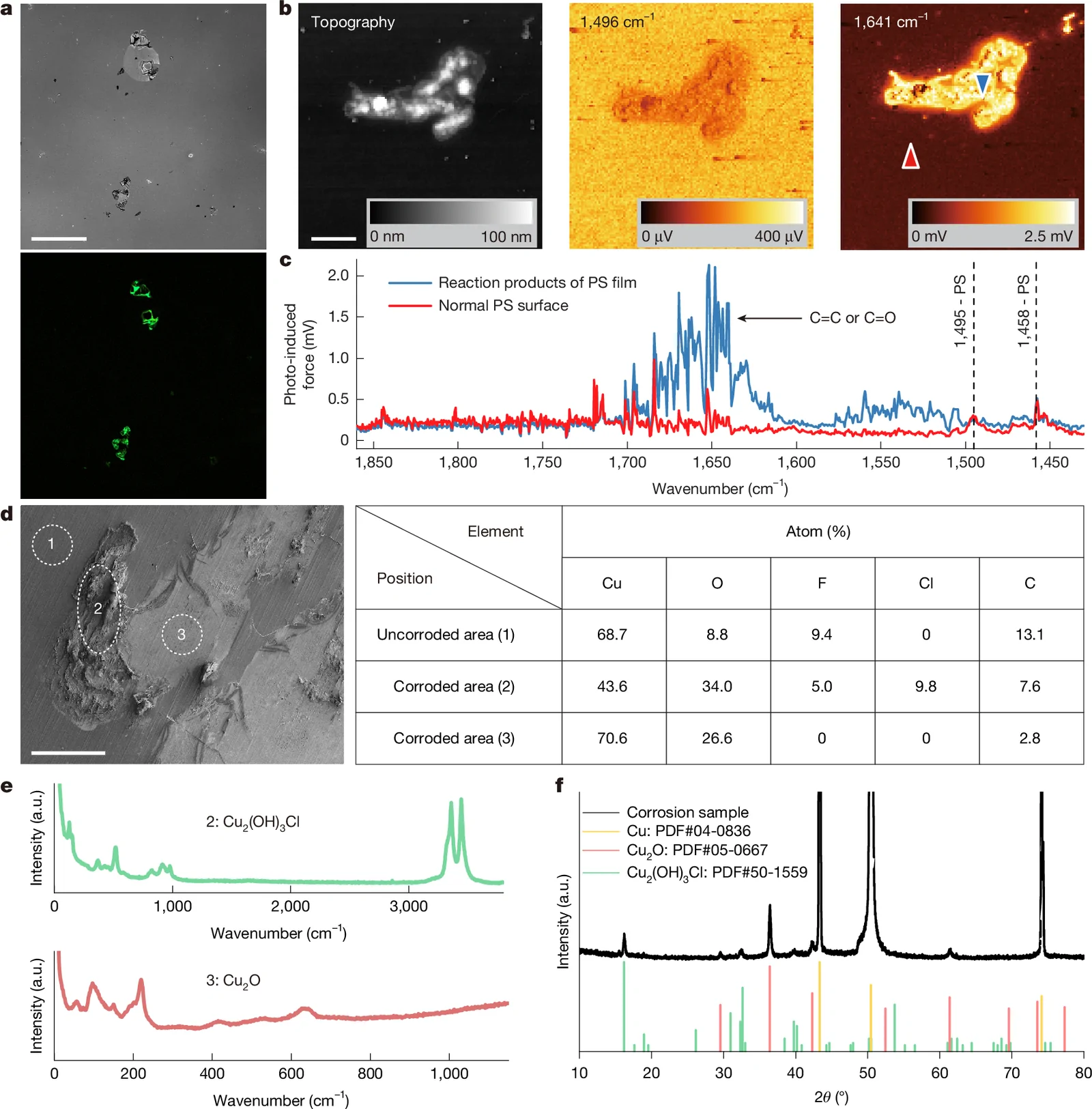
Products created by charged-drop impacts. **a**, Reflection and fluorescence confocal microscopy images of a 200-nm-thick PS film after impact of 3,000 charged water drops generated on a PFOTS-on-quartz plate. Excitation: 488 nm; fluorescence detection: 517–696 nm. **b**, AFM topography and nano-IR response of a region containing reaction products and normal PS. Nano-IR images were recorded by PiFM at 1,496 cm^−1^ (absorption of PS) and 1,641 cm^−1^. **c**, IR spectra were recorded in PiFM mode by varying the wavenumber of the IR light between 1,400 and 1,900 cm^−1^. Blue and red spectra correspond to the reaction products (blue triangle in **b**) and pristine PS regions (red triangle in **b**), respectively. Reaction products exhibit strong IR signals between 1,600 and 1,700 cm^−1^, suggesting the presence of C=C or C=O double bonds. **d**, SEM image and EDS analysis of 60-nm Teflon-coated Cu foil after 50,000 impacts of charged drops (35 µl 10 mM NaCl). Region 1 is an uncorroded area and regions 2 and 3 are corroded. EDS results showed a loss of fluorine and carbon, an increase in oxygen and the presence of chlorine in area 2, indicating that the corrosion products are mainly oxides with some chlorides. **e**, Raman spectra indicated that area 2 mainly contained basic copper chloride, whereas area 3 mainly contained cuprous oxide. **f**, XRD analysis of the corroded area. The diffraction peaks were dominated by pure copper, with weaker peaks corresponding to cuprous oxide and basic copper chloride based on comparison with the Powder Diffraction File (PDF) database. Because the X-ray beam was 1 mm wide, it covered both the corroded areas and the surrounding Cu. Scale bars, 200 µm (**a**); 1 µm (**b**); 1 mm (**d**). a.u., arbitrary units. Source data

**Extended Data Fig. 8:**
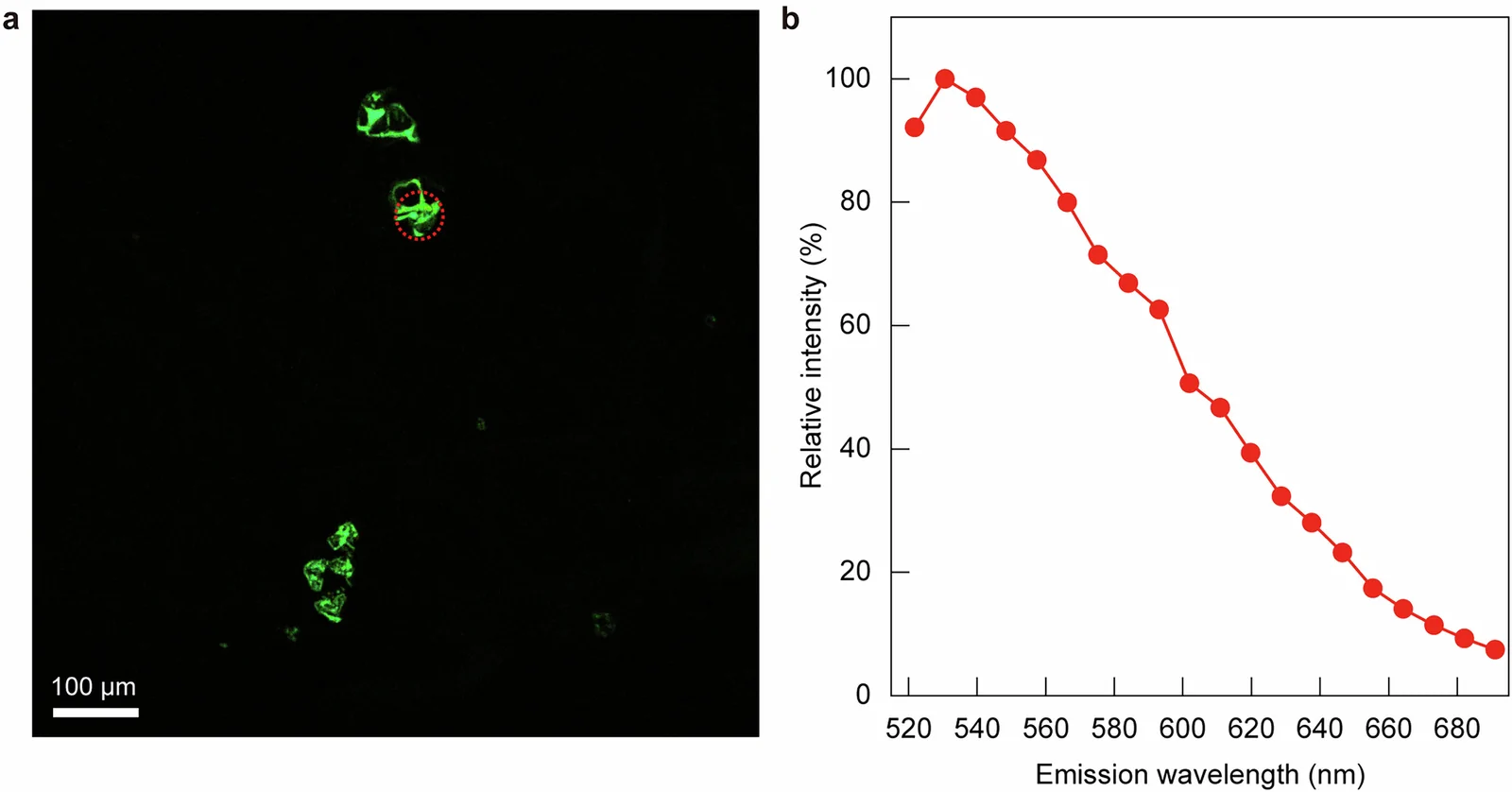
Fluorescence of products from PS film. **a**, Confocal microscopy fluorescence mode image of a 200-nm-thick PS film on copper after impact of 3,000 charged water drops, which had slid down a PFOTS-on-quartz plate. Excitation: 488 nm, fluorescence detection range: 517–696 nm. **b**, Spectral profile of the detected fluorescence as recorded using the lambda mode of the LSM 880 confocal microscope (red circle in **a**). Source data

As well as morphological damage, dielectric breakdown can cause chemical changes within the coating, resulting in new substances forming. Confocal microscopy images of a 200-nm-thick PS film on copper after 3,000 water drop impacts (Fig. 4a) showed the appearance of rough regions on the smooth PS surface in reflection mode. The fluorescence mode further revealed that some rough regions emitted fluorescence on 488-nm excitation, with broad emission spectra covering 520–680 nm (Extended Data Fig. 8). PS contains benzene rings and does not form a conjugated π-electron cloud system. Therefore the observed fluorescence does not originate from the PS but from the products of a reaction that have such a π-electron cloud system.

AFM-based nano-infrared (IR) analysis provided further characterization of the reaction products in the PS films^41,42^. The topography image showed a roughened region corresponding to reaction products surrounded by PS (Fig. 4b). Nano-IR imaging at 1,495 cm^−1^, corresponding to the aromatic C=C stretching of PS, showed lower absorption in the roughened area than in the surroundings, whereas imaging at 1,641 cm^−1^ produced an inversion of the contrast, indicating chemical bonds not present in PS. IR spectra recorded from the roughened and flat areas (Fig. 4c) both exhibited the characteristic PS peaks at 1,458 cm^−1^ and 1,495 cm^−1^, corresponding to the aromatic C=C stretching of PS (ref. ^42^). However, only the roughened area showed a distinct broad IR absorption band between 1,600 cm^−1^ and 1,700 cm^−1^, a range associated with the double-bond stretching vibration of C=C and carbonyls C=O (refs. ^43,44^). These two types of double bond and the benzene rings possibly form a conjugated π-electron cloud system, thus having fluorescent properties.

**Extended Data Fig. 9:**
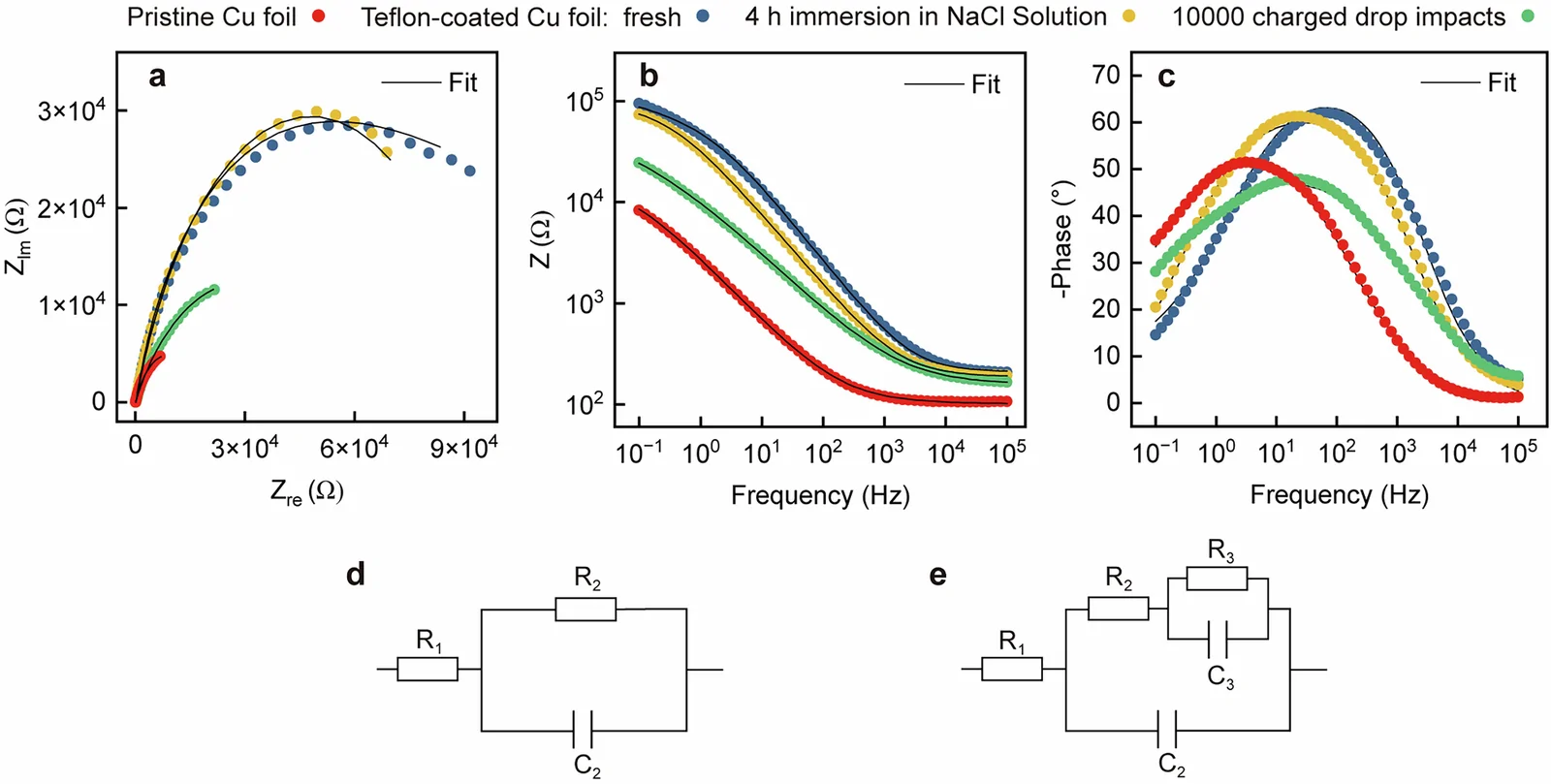
EIS evaluation of coating damage caused by charged-drop impacts. A Teflon-coated Cu foil sample was subjected to 10,000 charged-drop impacts (35 μl, 10 mM NaCl, sliding over FEP film). For comparison, another Teflon-coated Cu foil sample was continuously immersed in 10 mM NaCl solution for 4 h, which is approximately equivalent to the cumulative contact time between the drops and the surface during 50,000 drop impacts. Fresh Teflon-coated Cu foil and pristine Cu foil were included as references. EIS was performed using a potentiostat (Autolab PGSTAT204) in a three-electrode configuration with 10 mM aqueous NaCl as the electrolyte, consistent with the drop impact experiments rather than the 600 mM NaCl solution commonly used in conventional corrosion tests to avoid further effects from the high salt concentration. **a**,**b**, Nyquist plots (**a**) and Bode modulus plots (**b**) show a more pronounced reduction in coating impedance after charged-drop impacts than after continuous immersion. **c**, Bode phase angle plots further reveal a lower and narrower phase-angle peak after charged-drop impacts. This comparison indicates that the charged-drop impacts cause more severe damage to the coating than continuous immersion. **d**,**e**, Equivalent circuit models used to fit the EIS data of pristine Cu foil (**d**) and the three Teflon-coated Cu foil samples (**e**). The black lines in **a**–**c** represent the fitting results. R_1_: solution resistance; R_2_: charge transfer resistance; R_3_: coating resistance; C_2_ and C_3_ represent the double-layer capacitance and coating capacitance; *n*: exponent of the C. All of the C elements are constant phase elements. The fitted parameters related to the coating performance are listed in Extended Data Table 1. Source data

To demonstrate corrosion under broader conditions, we used commercial copper foil instead of the sputtered copper on quartz and coated with a 60-nm-thick Teflon film by dip-coating. Charged drops damaged the coating through dielectric breakdown. Electrochemical impedance spectroscopy (EIS) results consistently demonstrated that the initially excellent barrier properties of the coating were degraded after 10,000 charged-drop impacts (Extended Data Fig. 9 and Extended Data Table 1). Under the same salt concentration, charged-drop impacts caused much more coating damage than continuous immersion in the bulk solution. After approximately 50,000 charged drops had hit the surface, corrosion of more than 1 mm in size occurred on the surface (Fig. 4d). Scanning electron microscopy with energy-dispersive X-ray spectroscopy (SEM-EDS) elemental analysis showed that, relative to area 1, areas 2 and 3 showed substantially increased oxygen contents and decreased fluorine and carbon contents, with chlorine also being detected in area 2 (right of Fig. 4d). The decrease in fluorine and carbon indicated damage to the Teflon film in areas 2 and 3, whereas the increase in oxygen suggested oxide-dominated corrosion products and the chlorine signal in area 2 implied the presence of chlorides.

To further analyse the corrosion products, confocal Raman spectra collected over areas 2 and 3 (Fig. 4e) identify basic cupric chloride (Cu_2_(OH)_3_Cl)^45^ and cuprous oxide (Cu_2_O)^46^ as main corrosion products in area 2 and area 3, respectively. X-ray diffraction (XRD) analysis of the samples (Fig. 4f) revealed dominant diffraction peaks that correspond to metallic copper, reflecting that the corrosion products constitute a small fraction of the overall copper foil sample. But minor diffraction peaks corresponding to basic cupric chloride (Cu_2_(OH)_3_Cl) and cuprous oxide (Cu_2_O) are also observed, consistent with the Raman results.

The above results reveal the occurrence of corrosion phenomena. Corrosion is initiated by localized dielectric breakdown of the coating induced by charged drops. As a result, the underlying metal becomes exposed to the aqueous drop environment. As more charged drops hit the surface, the defective area gradually grows (Extended Data Fig. 7). The change in wettability in the defective area promotes drop retention and enhances direct contact with the metal surface, resulting in a localized potential difference at the liquid–solid interface that drives the formation of micro-galvanic cells and subsequent electrochemical corrosion. Also, charged drops can further influence the corrosion process by inducing external electric fields that alter the local electrode polarization state and promote the formation of strongly oxidative species inside drops (detail shown in Supplementary Information Discussion 2). The corrosion mechanism of charged drops is universally applicable. We carried out a series of experiments to validate this, involving variations in drop ionic composition, ambient oxygen (Supplementary Figs. 5–14) and metal substrate (surface blistering on aluminium; Supplementary Information Discussion 3).

## Control of corrosion position

In the experiments described above, there were two separate stages: charging of the water drop and subsequent corrosion, which happened on two different surfaces. Corrosion caused by slide electrification can also occur on a single sample consisting of a structured substrate composed of an electrical insulator and a conductor. A rectangular mask was used to cover two-thirds of a quartz plate and a 35-nm-thick copper film was sputtered onto the uncovered area. Then a 60-nm-thick Teflon film was dip-coated onto the entire substrate surface, followed by annealing at 160 °C for 24 h in vacuum. This method produced a flat, smooth and chemically uniform Teflon surface on a hidden structured substrate.

**Fig. 5:**
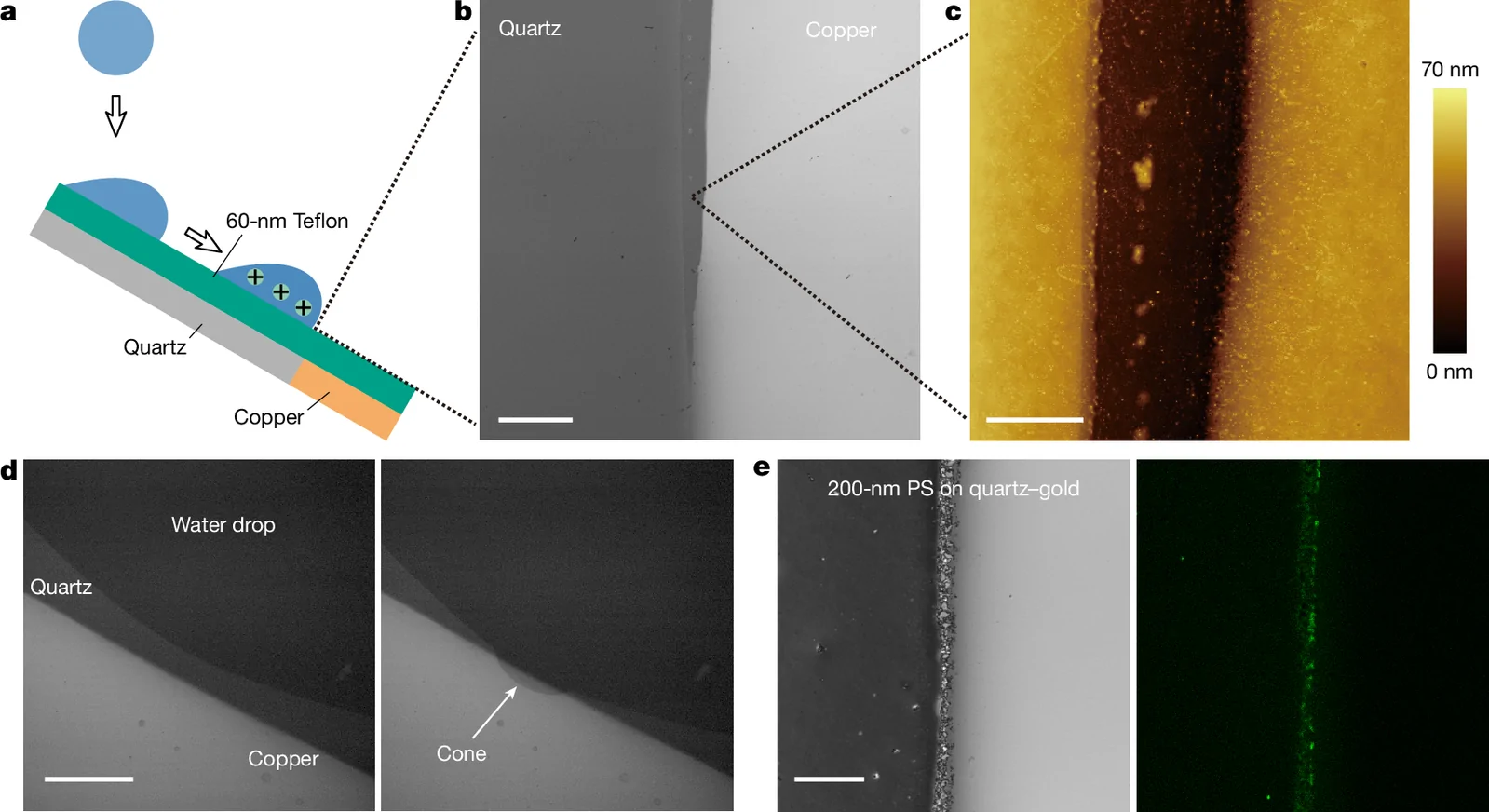
Sliding charged water drops induce corrosion at a specific position. **a**, Schematic of the sliding water drops experiment. The substrate is composed of quartz and copper, which is coated with a 60-nm-thick Teflon film. **b**, Reflection mode confocal microscopy image of the quartz–copper boundary area after sliding 3,000 water drops over it (copper appears bright and quartz dark). The corrosion pattern is distributed along the boundary. **c**, AFM image of the boundary area. Sliding charged water drops cause a trench-like defect on the surface. **d**, Bottom-view high-speed camera image of the water drop’s advancing contact line. When the advancing contact line approaches the quartz–copper boundary, a small cone forms at the front. **e**, Reflection and fluorescence mode confocal microscopy images of the corrosion of a PS film coating. Excitation wavelength was 488 nm and the fluorescence detection range was 535–696 nm. Scale bars, 50 µm (**b**); 10 µm (**c**); 500 µm (**d**); 50 µm (**e**).

When a water drop slid down from the top, a charge was generated in the Teflon-on-quartz area. Then the drop moved over the Teflon-on-copper area (Fig. 5a). After 3,000 water drops, a corrosion pattern appeared along the quartz–copper boundary (Fig. 5b). AFM images revealed the formation of a trench-like defect (Fig. 5c). No defects were observed after electrically neutral drops slid (Supplementary Fig. 17). In the case of the sliding drop, when the advancing contact line of the water drop reached the boundary to the hidden copper layer, a conical liquid meniscus formed and jumped ahead, leading to a local discharge of the drop (Fig. 5d). Using a PS film instead of Teflon leads to fluorescence in the boundary area (Fig. 5e). The corrosion caused by the sliding water drops was always located in the boundary area, indicating that water drop impact is not necessarily required for corrosion to occur; sliding alone can cause corrosion.

We predict that similar corrosion effects on heterogeneous or composite materials occur, in which a higher dielectric permittivity or conductive material is present in an insulating material with low dielectric permittivity as matrix (Supplementary Fig. 18). A drop sliding over such material would discharge when the advancing contact line reaches those parts with high dielectric permittivity, even if they are hidden underneath a coating.

## Conclusion

In our work, we describe a previously unrecognized effect: naturally charged drops can cause a coating to break down electrically, thereby initiating or exacerbating corrosion of the coated metals. This effect remained overlooked because protocols to quantify charge separation at receding contact lines have only been developed in recent years, enabling a fundamental understanding of slide electrification. Similarly, the importance of drop charging in contact angle hysteresis and drop friction has been underestimated. Charged drops can be generated naturally in clouds, thunderstorms, ocean waves, fountains and waterfalls or when sliding over hydrophobic materials before hitting the coated metal. They also occur in industrial processes such as electrostatic spraying, inkjet printing and reactions in chemical and pharmaceutical production. Consequently, this cause of corrosion may be relevant in many everyday or industrial processes.

## Methods

### Natural and commercial materials

*Tradescantia spathacea* leaves were obtained from potted plants in our office. PVC foam boards (PVC-Schaumplatte Fixmaß, Bauhaus AG, 250 × 500 × 3 mm^3^) and transparent PS glass (Hobbyglas Owocor, Bauhaus AG, 250 × 500 × 2 mm^3^) were purchased from a local hardware store. Fluorinated ethylene propylene (FEP) film (Nenull, 140 × 200 × 0.15 mm^3^), copper foil tape (3M 1181 Kupferband leitend, 0.04 mm, 50 mm × 16.5 m), super-hydrophobic spray (Glaco Mirror Coat Zero, 40 ml) and PS sheets (Evergreen Scale Models, 130 μm) were purchased from an online shop.

### Sample preparation

Sixteen sample types were prepared:

1. PFOTS layers on quartz. Quartz plates (75  × 25 × 1 mm^3^, proQuarz GmbH) were treated with O_2_ plasma at 300 W for 10 min (Femto low-pressure plasma system, Diener electronic). Using CVD, 1H,1H,2H,2H-PFOTS (97%; Sigma-Aldrich) was then coated on the surface of the quartz plate. The cleaned quartz plates were subsequently placed in a vacuum glass container together with a vial containing 0.5 ml PFOTS. Then we evacuated the container to a pressure below 100 mbar, closed the pump and let it react for about 30 min. Finally, the quartz plates were rinsed with ethanol to remove any unbound silane molecules.
2. PFOTS layers on ITO glass. ITO glass (75 × 25 × 1.1 mm^3^, surface resistivity 30–60 Ω sq^−1^, Sigma-Aldrich) were treated with O_2_ plasma at 300 W for 10 min (Femto low-pressure plasma system, Diener electronic). Using CVD, 1H,1H,2H,2H-PFOTS (97%; Sigma-Aldrich) was then coated on the surface of the ITO glass. The cleaned ITO glass was subsequently placed in a vacuum glass container together with a vial containing 0.5 ml PFOTS. Then we evacuated the container to a pressure below 100 mbar, closed the pump and let it react for about 30 min. Finally, the ITO glass was rinsed with ethanol to remove any unbound silane molecules.
3. 60-nm Teflon-coated copper. To obtain flat copper substrates, a 35-nm-thick copper layer was sputtered onto a quartz plate. A 60-nm-thick Teflon film was then coated onto the copper by dip-coating with a pulling speed of 10 mm min^−1^ from 1 wt% Teflon AF 1600 (*ε*_r_ = 1.9, Sigma-Aldrich) in FC-75 (97%, Fisher Scientific). Finally, the Teflon-coated copper samples were heated in an oven at 160 °C under vacuum for 24 h. The film thickness was measured by a profiler (P-7 stylus profiler, KLA-Tencor).
4. A 60-nm-thick PS film was coated onto the copper by dip-coating with a pulling speed of 60 mm min^−1^ from 2 wt% PS (molecular weight 192 kg mol^−1^, *ε*_r_ = 2.6; Sigma-Aldrich) in toluene (99.8%, Sigma-Aldrich). The sample was then heated in an oven at 120 °C under vacuum for 24 h.
5. A 200-nm-thick PS film was coated onto copper by dip-coating with a pulling speed of 40 mm min^−1^ from 4 wt% PS in toluene. The samples were then annealed (120 °C under vacuum for 24 h).
6. A 1-μm-thick PS film was coated onto copper by dip-coating with a pulling speed of 20 mm min^−1^ from 10 wt% PS in toluene. The samples were then annealed (120 °C under vacuum for 24 h).
7. A 5-μm-thick PS film was coated onto copper by dip-coating with a pulling speed of 10 mm min^−1^ from 20 wt% PS in toluene. The samples were then annealed (120 °C under vacuum for 24 h).
8. 200-nm PS-coated gold samples. A 35-nm gold layer was sputtered onto quartz plates. A 200-nm PS film was then coated onto the gold layer by dip-coating, as described previously.
9. 60-nm SiO_2_-coated gold samples. First, a 35-nm gold layer was sputtered onto quartz plates. Then a 60-nm SiO_2_ layer was sputtered onto a gold layer.
10. 60-nm Teflon-coated aluminium sample. First, a 100-nm aluminium layer was deposited onto glass slides (76.2 × 25.4 × 1 mm^3^, Sail Brand) by vacuum thermal evaporation. Then a 60-nm Teflon film was coated on the aluminium layer by dip-coating with a pulling speed of 10 mm min^−1^ from a solution of 1 wt% Teflon AF 1600 (*ε*_r_ = 1.9, Sigma-Aldrich) in FC-75 (97%, Fisher Scientific). Finally, the samples were annealed (160 °C under vacuum for 24 h).
11. PFOTS layers on aluminium. First, a 100-nm aluminium layer was deposited onto glass slides. The sample was then treated with O_2_ plasma at 300 W for 10 min and PFOTS was deposited by CVD, as described previously.
12. 60-nm Teflon-coated quartz–copper samples (for sliding experiments). We used a rectangular mask to cover two-thirds of the quartz plate (75 × 25 × 1 mm^3^, proQuarz GmbH) and 35 nm of copper was sputtered onto the uncovered area. After removing the mask, we obtained a plate with one-third covered by Cu and two-thirds comprising the quartz surface. The boundary between the quartz and copper areas formed a sharp straight line. Finally, a 60-nm Teflon film was coated on this plate by dip-coating with a pulling speed of 10 mm min^−1^ from 1 wt% Teflon AF 1600 (*ε*_r_ = 1.9, Sigma-Aldrich) in FC-75 (97%, Fisher Scientific). Finally, the samples were annealed (160 °C under vacuum for 24 h).
13. 200-nm PS-coated quartz–gold samples (for sliding experiments). We used the same method as above to make a substrate of two-thirds quartz and one-third 35-nm gold. A 200-nm PS film was then coated on this plate by dip-coating with a pulling speed of 40 mm min^−1^ from 4 wt% PS in toluene. Then the samples were annealed (120 °C under vacuum for 24 h).
14. 60-nm Teflon-coated commercial copper foil samples. A 40-μm-thick single-sided copper foil tape (3M 1181 Kupferband leitend, 0.04 mm, 50 mm × 16.5 m) was cut into dimensions of 50 mm × 25 mm, stacked face to face on the adhesive side and cleaned with ethanol. A 60-nm-thick Teflon film was then coated onto the copper by dip-coating with a pulling speed of 10 mm  min^−1^ from 1 wt% Teflon AF 1600 (*ε*_r_ = 1.9, Sigma-Aldrich) in FC-75 (97%, Fisher Scientific). Finally, the samples were heated in an oven at 160 °C under vacuum for 24 h.
15. 500-nm super-hydrophobic coating commercial Copper foil samples. A 40-μm-thick single-sided copper foil tape (3M 1181 Kupferband leitend, 0.04 mm, 50 mm × 16.5 m) was cut into dimensions of 50 mm × 25 mm, stacked face to face on the adhesive side and cleaned with ethanol. A commercial super-hydrophobic spray (Glaco Mirror Coat Zero) was applied to copper foil from a distance of approximately 0.5 m for 3–4 s. The sample was then left undisturbed for 30 min to allow the ethanol in the solution to evaporate.
16. A 12-μm-thick PS film was coated onto copper by dip-coating with a pulling speed of 10 mm min^−1^ from 30 wt% PS in toluene. The samples were then annealed (120 °C under vacuum for 24 h).

### Water drop impact experiments

We generated water drops with a volume of 35 μl water (<1 μS cm^−1^; Gibco, Thermo Fisher Scientific) containing 1 mM NaCl (diluted from 1 M NaCl solution, Carl Roth) through a grounded syringe needle (electrically neutral). The needle was connected to a peristaltic pump (MINIPULS 3, Gilson). Water drops were released from a height of about 4 cm from the sample surface at intervals of 12 s. More than 1,000 water drops were continuously released in each experiment. The sample was tilted by 10° so that, after impact, the water drop rolled off the surface. Each water drop always showed the same behaviour (Supplementary Fig. 19).

In the experiments of the spontaneously charged water drops that induce corrosion, the water drops first fell onto the top of a 50° tilted surface (*Tradescantia spathacea* leaves, PVC foam board, PS glass, FEP film or PFOTS-on-quartz plate) at intervals of 12 s from a release height of about 5 mm. Driven by gravity, water drops slid down roughly 4 cm and then left the tilted surface. During this process, the water drops undergo slide electrification, causing them to accumulate charge and deposit opposite charge onto the tilted surface. To achieve defined electrical conditions, the tilted surfaces were placed on a grounded metal plate. After the water drops left the tilted surface, they carried the electrical charge and, after falling about 5 mm, hit the Teflon-coated copper or other samples. A similar discharge cone was observed at different falling heights (Supplementary Fig. 20). As well as the 1 mM NaCl, there were more salt solutions (10 mM NaCl (Carl Roth), 100 mM NaCl (Carl Roth), 10 mM KCl (Carl Roth), 10 mM NaBr (Carl Roth), 10 mM KNO_3_ (Carl Roth), 10 mM ZnSO_4_ (Fluka) and also deionized water (<1 μS cm^−1^; Gibco, Thermo Fisher Scientific) and rainwater collected from the Mainz area.

The charge deposited by the drops on the tilted surface reduced the slide electrification effect of subsequent drops. Because it takes several minutes or more for the surface to naturally return to electrical neutrality, we needed to speed up the experimental process. To do this, we used an ionizing air blower (Aerostat PC ionizing air blower, Simco-Ion), which continuously neutralized the charge on the tilted surfaces during the experiment. This ensured that each subsequent water drop had the same slide electrification effect. In practice, tilted surfaces were neutralized in the 12 s between subsequent drops. Similarly, more than 1,000 water drops were continuously released in each experiment. Surface neutralization created by the ionized air took several seconds and was negligible while drops were sliding (about 100 ms) but effective in the intervals between drops. We also tested the case without the ionizing air blower and still observed surface corrosion (Supplementary Fig. 21).

For both experiments, we used a side-view high-speed camera (Photron, FASTCAM MINI UX100, 25,000 fps, resolution 1,280 × 200, with 1× SilverTL Telecentric Lens, Edmund Optics) to observe the behaviour of water drops when they hit the Teflon-coated copper surface. For the experiment observing the evolution of surface corrosion patterns from the bottom in situ, we used a non-high-speed camera (FLIR Blackfly S) for recording.

### Water drop sliding experiments

The samples for the sliding experiments were placed on a grounded metal plate tilted at 50°. 35 μl water drops containing 1 mM NaCl were released onto the top of a tilted sample from a grounded syringe needle at intervals of 12 s, with a release height of about 5 mm. We used a peristaltic pump (MINIPULS 3, Gilson) to continuously deliver solution from the tank to the syringe. During the experiment, the ionizing air blower (Aerostat PC ionizing air blower, Simco-Ion) was always turned on. In each experiment, about 3,000 water drops were released continuously. We used a bottom-view high-speed camera (Photron, Phantom TMX 7510, 40,000 fps, resolution 1,280 × 800, with 10× UPlanSApo Microscope Objective, Olympus) to observe the behaviour of the water drops when they slid down tilted samples.

### Confocal laser scanning microscopy imaging

Surface morphology and fluorescent properties of samples were imaged using a confocal microscope (LSM 880, Carl Zeiss) equipped with a Zeiss Plan-Apochromat 10×/0.45 objective. An argon-ion laser (*λ* = 488 nm) coupled with an optical fibre to the microscope was used for excitation. The confocal observation volume was positioned on the surfaces studied. Images were recorded either in reflection or in fluorescence mode using an appropriate dichroic mirror and a spectral detection unit (Quasar, Carl Zeiss). This unit comprises a diffraction grating and a 32-channel GaAsP multianode photomultiplier array detector. While recording the fluorescence images, the maximum spectral range of the detected emission light was 517–696 nm. The emission spectra were recorded using the so-called lambda mode of the instrument. In this mode, the spectral range of 520–690 nm was distributed between the 32 channels and the signal in every channel was detected separately. The 3D image of the surface morphology (Supplementary Figs. 15 and 23) was obtained by another confocal microscope (confocal white light microscope, µsurf, NanoFocus AG) equipped with a 20×/0.46 objective, using point-by-point line scanning laser profilometry.

### Charge measurements

Charge measurements were conducted using a current amplifier (response time: 0.8 ms, FEMTO DDPCA-300). We recorded the current signal of water drops or metal substrates during impact using a National Instruments data acquisition board (USB-6366 X Series).

Measurement of water drops: the current amplifier was connected to wires with a diameter of 0.7 mm made of tungsten serving as electrodes. The tungsten wire was coated with a layer of gold to enhance electrical conductivity. We measured the current of drops after sliding 4 cm on the surface (Extended Data Figs. 1 and 5a). Also, we measured the current of drops bouncing off the surface (Extended Data Fig. 5c). In both cases, the water drops touched the electrode and caused a current flow. The contact to the wire electrode corresponds to 0 ms (*t*_0_) and the drop discharged until *t*_1_. After *t*_1_, there is a low positive current of about 10 nA until the rear of the drop detaches from the electrode after roughly 20 ms. This low positive current is the result of the continued deposition of negative charge on the surface as the drop moves while still in contact with the electrode. For all samples except for the *Tradescantia spathacea* leaves, *t*_1_ was typically 2 ms. For the leaves, *t*_1_ was about 10 ms. The integral of the current from *t*_0_ to *t*_1_ is the amount of charge generated by slide electrification. We also measured the drop current and charge under different salt concentrations, release intervals and natural rainwater (Supplementary Figs. 4, 21 and 22).

Measurement of the current of metal substrates: here the current amplifier was connected directly to the metal layer. When the water drop hit the surface, a current signal was detected as well (Extended Data Fig. 5b). The contact of the drop onto the surface corresponded to 0 ms and the drop discharged until *t*_1,_ which was about 2 ms. The integral of the current from *t*_0_ to *t*_1_ is the amount of charge transferred from the water drop to the metal.

### AFM and AFM-IR measurements

Details of the surface morphology were studied using AFM (Dimension Icon, Bruker) in tapping mode. The developed surfaces were uniform and smooth (Supplementary Figs. 23 and 24). Commercial Cu foil had a rough surface and its roughness was slightly reduced after coating with a Teflon film (Supplementary Fig. 25). The cantilever had a nominal resonance frequency of 300 kHz and a spring constant of 26 N m^−1^ (OTESPA, OPUS). The scan sizes were 0.5 × 0.5, 20 × 20 or 50 × 50 μm^2^.

The products resulting from dielectric breakdown on the PS film were measured using an AFM-based nano-IR method (Vista One, Molecular Vista). The nano-IR response was recorded in photo-induced force microscopy (PiFM) mode. To obtain images of the surface topography, we excited the second eigenmode of the cantilever resonance frequency and kept the oscillation amplitude constant, using an electronic feedback circuit. The first eigenmode of the cantilever resonance frequency was used to record the nano-IR response. The incident IR light was modulated using the difference frequency between the first and second eigenmodes of the cantilever. This difference frequency was tuned to a maximum response amplitude at the first eigenmode.

### SEM-EDS measurements

The determinations by SEM were performed with a HITACHI SU8000 (Hitachi High-Technologies Europe GmbH). The scanning electron microscope was coupled to an XFlash 5010 detector, an X-ray detector that allows simultaneous EDS-based elemental analyses.

### Raman measurements

Raman measurements were performed using a WITec confocal Raman spectrometer (alpha300 R, 10× objective, 600/1,200 grooves mm^−1^ grating, 5 mW) with a 532-nm excitation and 120 s integration. The pristine copper foil was included as a reference sample (Supplementary Fig. 26a).

### XRD measurements

XRD measurements were performed using a Rigaku SmartLab diffractometer with a rotating Cu anode (8 keV, *λ* = 1.5406 Å), a Kβ filter and a HyPix-3000 detector. The sample was placed on a rotating sample stage and measured in *θ*–*θ* geometry in the range 10° < 2*θ* < 80° at a rate of 1° min^−1^. The X-ray beam has a width of 1 mm. The pristine copper foil was included as a reference sample (Supplementary Fig. 26b).

### EIS measurements

EIS was carried out using a Metrohm Autolab N series potentiostat (Autolab PGSTAT204) in a three-electrode configuration. Samples were mounted horizontally in a flat-cell configuration, with the exposed working area defined by an O-ring of diameter 6 mm. An Ag/AgCl electrode (3 M KCl) and a platinum wire were used as the reference and counter electrodes, respectively. The electrolyte was either 10 mM or 600 mM aqueous NaCl and measurements were performed in air at ambient temperature. Impedance spectra were recorded at open-circuit potential over a frequency range of 10^5^ Hz to 0.1 Hz using a sinusoidal perturbation of 10 mV. For 5-μm PS films, the perturbation amplitude was increased to 20 mV to improve the signal-to-noise ratio for these highly resistive samples. The EIS results demonstrated that the developed coatings greatly increase the interfacial resistance, indicating their excellent barrier properties (Supplementary Information Discussion 4). We also used a digital multimeter to test the barrier properties of the coating (Supplementary Fig. 28).

## Online content

Any methods, additional references, Nature Portfolio reporting summaries, source data, extended data, supplementary information, acknowledgements, peer review information; details of author contributions and competing interests; and statements of data and code availability are available at https://doi.org/10.1038/s41586-026-10941-6.

## Supplementary information

Supplementary Information This file contains four Supplementary Discussions, 28 Supplementary Figures, two Supplementary Tables and Supplementary References. It provides more calculations, control experiments, electrochemical measurements and materials characterization supporting the proposed mechanism of coating damage and corrosion induced by charged water drops.

Supplementary Video 1 High-speed recording of an uncharged water drop approaching and hitting a Teflon-coated copper surface.

Supplementary Video 2 High-speed recording of a charged water drop approaching and hitting a Teflon-coated copper surface

## Source data

Source Data Fig. 1

Source Data Fig. 2

Source Data Fig. 4

Source Data Extended Data Fig. 1

Source Data Extended Data Fig. 5

Source Data Extended Data Fig. 6

Source Data Extended Data Fig. 8

Source Data Extended Data Fig. 9

## Figures and Tables

**Extended Data Table 1:**
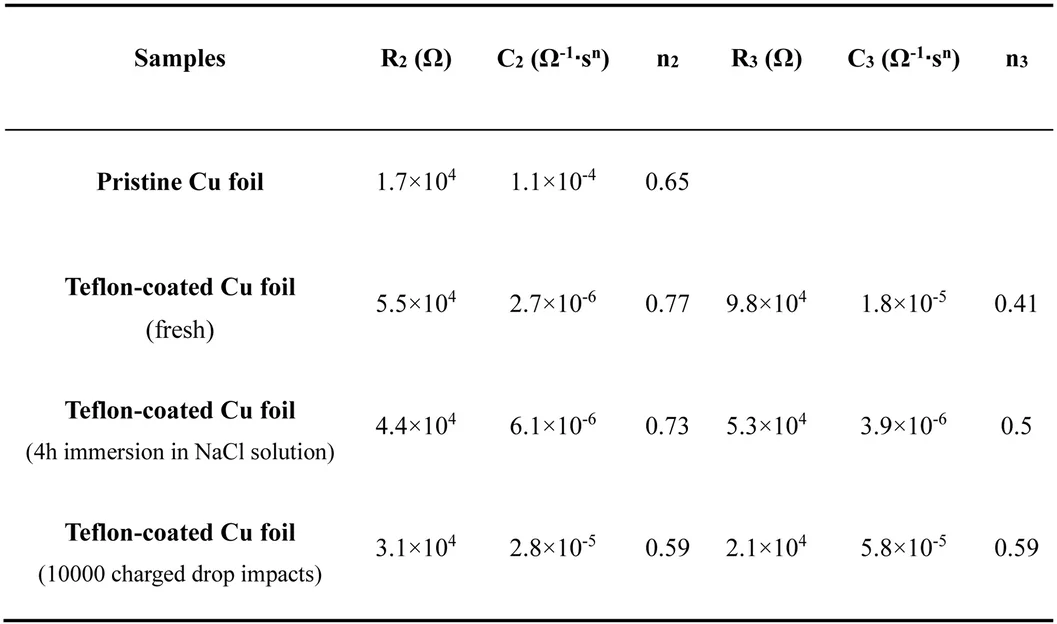
Fitting parameters for Extended Data Fig. 9

## Data Availability

The data supporting the findings of this study are available in the paper, the Extended Data and the Supplementary Information. Source data are provided with this paper.
