# Supplementary material for: Spontaneously charged water drops induce corrosion
Source: Nature. 2026 Aug 26;657(8131):393–9. doi: 10.1038/s41586-026-10941-6 (PMC13558045; doi:10.1038/s41586-026-10941-6)
Supplement: Supplementary file 1 [file 41586_2026_10941_MOESM1_ESM.pdf]

nature portfolio

https://doi.org/10.1038/s41586-026-10941-6

Supplementary information

Spontaneously charged water drops induce corrosion

In the format provided by the authors and unedited

Supplementary information

Spontaneously Charged Water Drops Induce Corrosion

Zhongyuan Ni[1], Xiaomei Li[1,2], Aaron D. Ratschow[1], Lin Jian[1], Xiaoteng Zhou[1,3], Pravash Bista[1], Diego Cortes[1], Gunnar Glasser[1], Haojian Luo[1], Shuai Chen[1,4], Jiyao Yu[1], Yongkang Wang[1], Katrin Amann-Winkel[1,5], Kaloian Koynov[1], Rüdiger Berger[1]*, Hans-Jürgen Butt[1]*

[1]*Max Planck Institute for Polymer Research, Mainz, 55128, Germany.*

[2]*South China Advanced Institute for Soft Matter Science and Technology, School of Emergent Soft Matter, South China University of Technology, Guangzhou 510640, China*

[3]*Department of Mechanical Engineering, Massachusetts Institute of Technology, Cambridge, MA, 02139, USA*

[4]*Institute of Inorganic Chemistry, University of Bonn, Bonn, 53121, Germany*

[5]*Institute of Physics, Johannes Gutenberg University Mainz, 55099 Mainz, Germany*

***Corresponding authors:** berger@mpip-mainz.mpg.de, butt@mpip-mainz.mpg.de

Table of Contents

Discussion 1: Joule heating estimate of the upper limit of coating damage size

Including text and Table S1 3-4

Discussion 2: Mechanism of corrosion induced by charged drops

Including text and Fig. S1-S3 5-9

Slide electrification effect of drops with different ion concentrations: Fig. S4 10

SEM-EDS measurements for different conditions: Fig. S5-S14 11-20

Discussion 3: Surface blistering on aluminum induced by charged drops

Including text and Fig. S15-S16 21-23

Comparison charged and electrically neutral sliding drops: Fig. S17 24

Spontaneous charged drop corrosion effect in composite sample: Fig. S18 25

Electrically neutral drops impacting a Teflon-coated copper surface: Fig. S19 26

Effect of the falling height of charged drops on discharge: Fig. S20 27

Charged drop impact experiment without ionizing air blower: Fig. S21 28

Slide electrification of rainwater drops: Fig. S22 29

Surface topography of samples: Fig. S23-S25.. 30-32

Raman and X-ray diffraction of pristine copper foil: Fig. S26..33

Discussion 4: EIS of sputtered Cu samples with coatings

Including text, Fig. S27, and Table S2... 34-37

Electrical properties of coatings: Fig. S28..38

Supplementary reference 39

Discussion 1: Joule heating estimate of the upper limit of coating damage size

Contents: supporting text and Table S1

For metal surfaces protected by coatings or passivation layers, charged water drops first induce dielectric breakdown, creating defects in the coating. Joule heating during dielectric breakdown can be used to estimate the upper limit of coating damage size.

To estimate the effect of Joule heating in the polymer film, we first consider the volume of polymer which can be heated from 25°C to its melting temperature by the electric energy stored in a charged drop, W. The electric energy is $W = \frac{Q^2}{2C} = \frac{Q^2 d}{2 \cdot A\varepsilon\varepsilon_0}$. Here, d is the layer thickness, C is the capacitance of the drop across the insulating layer, and A is the contact area of the drop. If all this energy is converted to heat a volume element of polymer V would heat up by $\Delta T = \frac{W}{c\rho V}$, c is the specific heat capacity of polymer, and ρ is the density of polymer. We consider that a cylindrical element of the film of radius r is heated. Then $V = \pi r^2 d$, where r is the radius of the cylinder which melts. Vice versa, to heat the polymer to its melting temperature the radius of the cylindrical element should not be larger than $r = \sqrt{\frac{W}{\pi c\rho d\Delta T}} = \sqrt{\frac{Q^2}{2\pi A\varepsilon\varepsilon_0 c\rho\Delta T}}$. It is independent on the thickness of the protective layer. The polymer film is efficiently cooled by the water and the underlying metal. For heat to equilibrate over a distance d a relaxation time $\tau \approx \frac{d^2}{2\kappa_T} = \frac{d^2 \rho c}{2k}$ is sufficient. In the following Table S1 the values parameters for PS and PTFE are given. The radii and relaxation times were calculated for d = 60 nm, Q = 1 nC, $A = 2.5\times10^{-5}$ m^2. According to the calculation (Table S1), the Joule heating generated by a single charged drop can cause damage with a maximum radius of about 700 nm in the 60 nm Teflon film.

Table S1. Parameters of polystyrene (PS) and polytetrafluoroethylene (PTFE)

	PS	PTFE
Density ρ (kg/m^3)	1040	2200
Dielectric permittivity ε	2.5	2.2
Specific heat capacity c (J/(kg K))	1250	1000
Thermal conductivity k (W/(K m))	0.17	0.24
Thermal diffusivity $\kappa_T = \frac{k}{\rho c}$ (m^2/s)	1.3×10^{-7}	1.1×10^{-7}
Melting point (°C)	240	327
r (μm)	1.01	0.70
τ (ns)	14	17

Discussion 2: Mechanism of corrosion induced by charged drops

Contents: supporting text and Fig. S1-S3

1. Classic salt water drop corrosion experiment (single-electrode corrosion)

We prepared a clean copper foil (40 μm thick) without any coating and placed a 1 mL aqueous drop (1 M NaCl) on the copper surface. The drop formed a hemispherical cap covering the copper surface. After natural evaporation of the drop, a distinct corrosion pattern appeared within the drop-covered area, as shown on the right of Fig. S1.

In this single-electrode system corrosion still occurs, although the electrolyte solution is in contact with only one metal (copper). The metal corroded because the surface was not microscopically uniform. Variations in grain orientation, dislocation density, impurity distribution, residual stress, and local environmental conditions (such as oxygen concentration or ionic gradients) can induce local differences in electrode potential. These differences result in the spontaneous formation of micro-galvanic cells, in which regions of lower potential act as local anodes and undergo metal dissolution, while regions of higher potential act as cathodes where reduction occurs.

As schematically shown on the left of Fig. S1, a gradient in oxygen concentration between the interior and edge of the drop is spontaneously formed because the oxygen which was dissolved in water was constantly consumed by the oxidation reaction. The drop edge, being directly exposed to air, maintains a relatively high oxygen concentration, while the center—shielded by a thicker liquid layer and a longer gas diffusion path—exhibits significantly lower oxygen availability. This non-uniform oxygen distribution leads to the formation of a differential aeration cell on the metal surface, which drives localized electrochemical reactions. In practice, the metal–electrolyte interface contains numerous microscopic anode and cathode areas that together constitute a complex system of microcells.

At the anode areas, copper undergoes oxidation and releases electrons:

$$Cu \rightarrow Cu^{1+} + e^-,\ Cu^{1+} \rightarrow Cu^{2+} + e^-$$

In cathodic regions, dissolved oxygen and water molecules combine with electrons to undergo reduction (under neutral or alkaline conditions):

$$O_2 + 2H_2O + 4e^- \rightarrow 4OH^-$$

Subsequently, the copper ions react with hydroxide ions to form insoluble corrosion products such as:

$$2Cu^{1+} + 2OH^{-} \rightarrow Cu_2O + H_2O$$

Chloride ions in the solution also promote corrosion, including destroying the passivation film, exposing fresh metal surfaces, enhancing electrolyte conductivity, and promoting electrochemical reaction rates[1]. In addition, chloride ions can combine with copper ions to form insoluble corrosion products:

$$2Cu^{2+} + 3OH^{-} + Cl^{-} \rightarrow Cu_2(OH)_3Cl$$

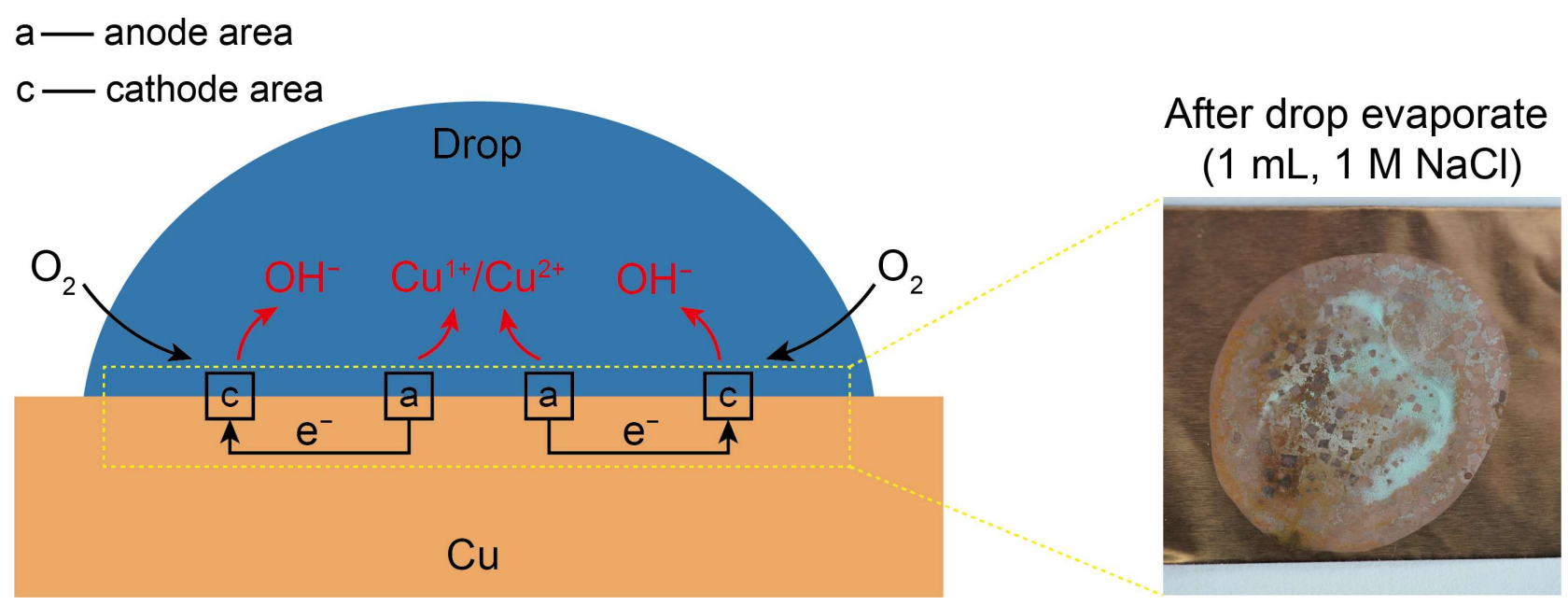

Fig. S1. Spontaneous corrosion of a single copper electrode in a salt water drop system. Left: A micro-galvanic cell forms between the interior and edge of the drop due to the oxygen concentration gradient. Right: The image of the copper foil surface after being corroded by salt water drop. A 1 mL drop (1 M NaCl) was placed on 40 µm thick copper foil surface (Width: 2 cm). After natural evaporation, surface salt crystals were removed by rinsing with deionized water, revealing the underlying corrosion morphology.

2. Charged water drop corrosion mechanism

Based on Section 1 (Joule heating estimate), we conclude that discharge associated with a single charged drop can create sub-μm-scale coating damage, as illustrated on the left of Fig. S2. In these regions, the underlying metal becomes exposed due to the local removal of the hydrophobic coating. This leads to a transition from hydrophobicity to hydrophilicity. When the underlying metal is exposed to air directly, dry corrosion, i.e., chemical corrosion, may occur; however, the corrosion rate is relatively slow.

As the number of charged drops impacting the surface increases, the defective area gradually expands (Extended Data Fig. 7). After impact, a portion of the drop remains on the surface and comes into direct contact with the exposed metal. It forms a micro-galvanic cell structure that is similar to those found in classical single-electrode corrosion systems. This results in electrochemical corrosion (i.e. wet corrosion) of the underlying metal, as indicated on the right of Fig. S2. Electrochemical corrosion proceeds much faster than chemical corrosion. In our experiments, charged drops impacted the surface approximately once every 12 s, while a single drop's impact lasted only about 100 ms. The residual drop volume increased with the extent of coating damage. Once the residual volume becomes large enough, it fails to fully evaporate before the next drop arrives. Therefore, the underlying metal was in direct contact with the liquid for most of the time, and its corrosion process was primarily governed by spontaneously formed micro-galvanic cells.

However, when a charged drop made contact with the surface, it created introduced a transient external electric field that altered the electrode potential at the metal–solution interface. This triggers a significant shift in the corrosion mechanism (Fig. S3). When a positively charged drop impacts the surface, the external electric field induces free electrons in the metal to migrate towards the metal–solution interface. This increases the electron density on the metal side of the interface, resulting in a negative shift in electrode potential, i.e. cathodic polarization. This enhances the supply of electrons at the interface, thereby promoting the reduction of electron acceptors such as O_2 or H^+. In our experiments, the drops consistently carried positive charge. However, in some cases, drops may also carry negative charge[2]. In that case, the external electric field drives free electrons away from the interface, decreasing the electron density on the metal side. This leads to a positive shift in electrode potential—i.e., anodic polarization—which in turn accelerates the oxidation and dissolution of the

metal. In addition, recent studies have shown that electrification may promote the formation of reactive species within drops[3-5], which could influence the corrosion process further. However, this possibility is still under discussion and requires further investigation.

Overall, the primary role of drop charge is to first trigger local discharge and dielectric breakdown in the coating, thereby causing localized coating failure and exposing the underlying metal. It creates a pathway for localized corrosion. This is the key initiating step in the entire corrosion process. Once the local coating has broken down, the subsequent corrosion process is no longer solely determined by the initial net charge carried by the drops; it gradually evolves into a more conventional localized electrochemical corrosion process. At this stage, factors such as dissolved oxygen, chloride ions, evaporation-induced concentration, and local galvanic effects may all contribute to the further corrosion of the Cu substrate. The charge carried by the drops continues to corrosion at this stage. The corrosion mechanism of charged drops, as described above, is generally applicable and can drive corrosion processes under a variety of conditions. Accordingly, corrosion damage was observed in all experimental cases presented in this work, including those involving variations in sample structure, solution composition, and oxygen environment.

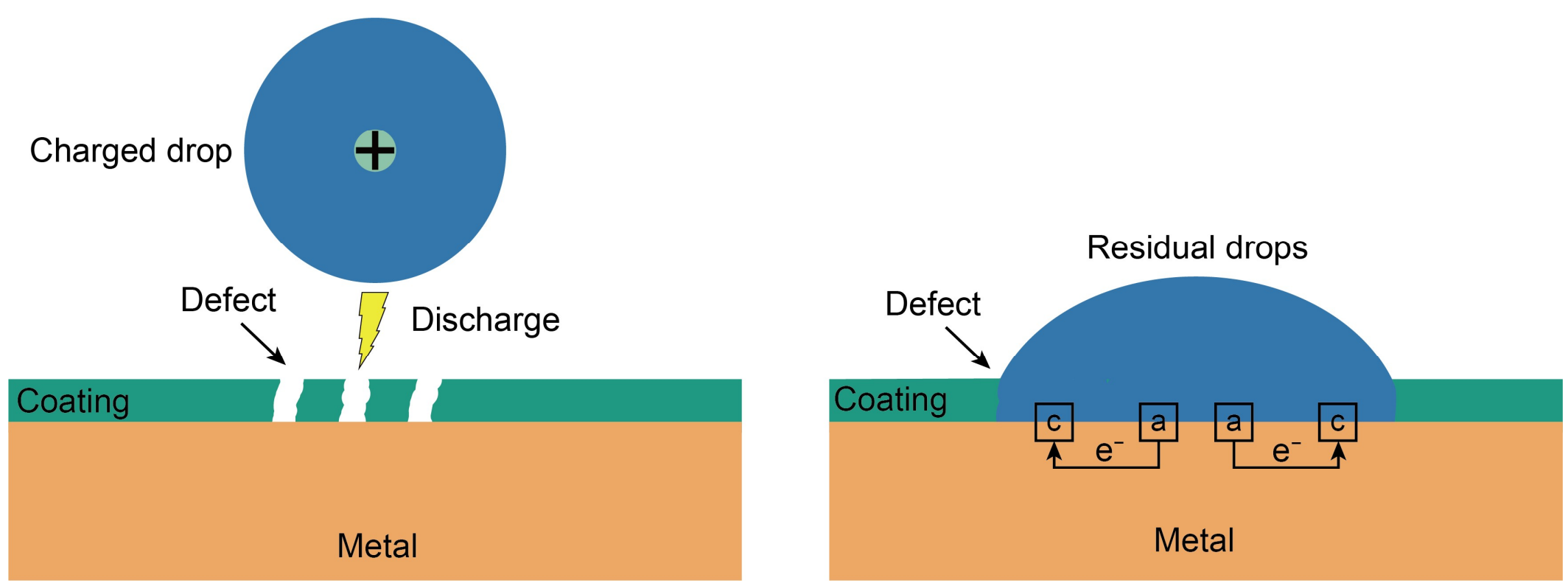

Fig. S2. Schematic diagram of coating failure and corrosion induced by charged drop. Left: coating defect induced by charged drop discharge. Right: localized corrosion at the defect are due to a micro-galvanic cell formed between the residual drops and the underlying metal.

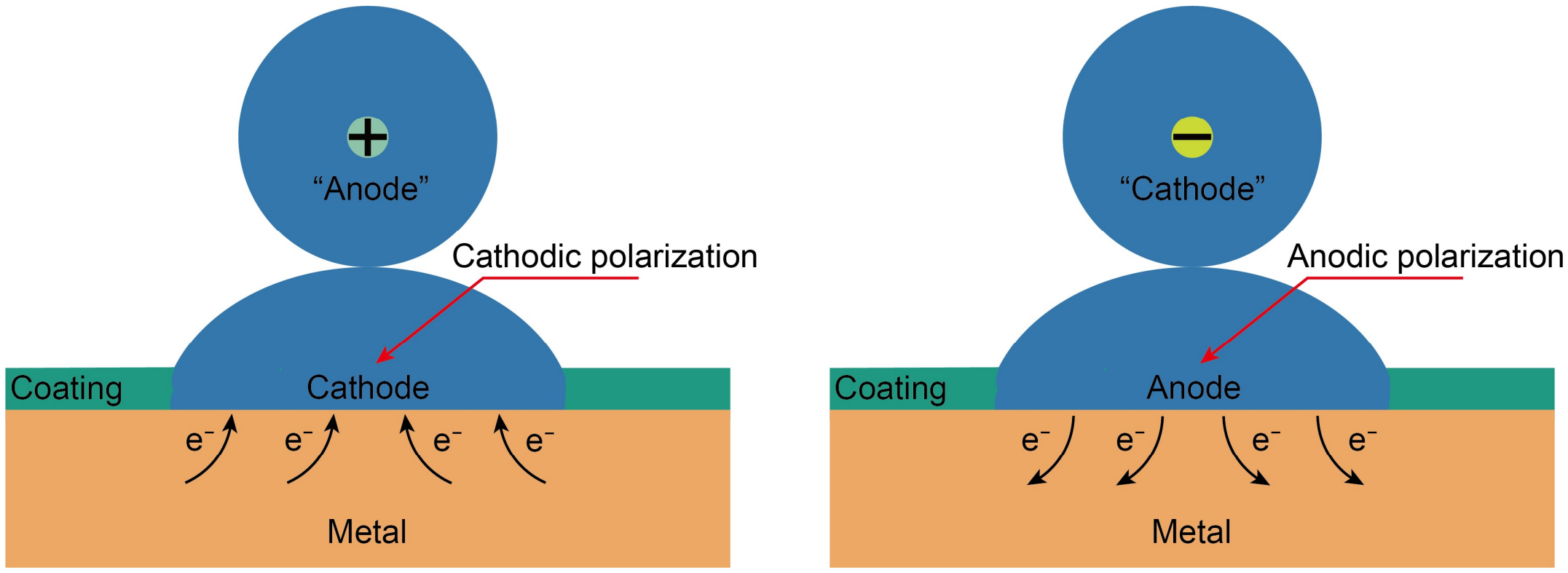

Fig. S3. Schematic diagram of the electrode potential polarization behavior. Left: The positively charged drop acts analogously to an anode, inducing cathodic polarization in the underlying metal upon impact. Right: The negatively charged drop acts analogously to a cathode, leading to anodic polarization of the metal upon impact.

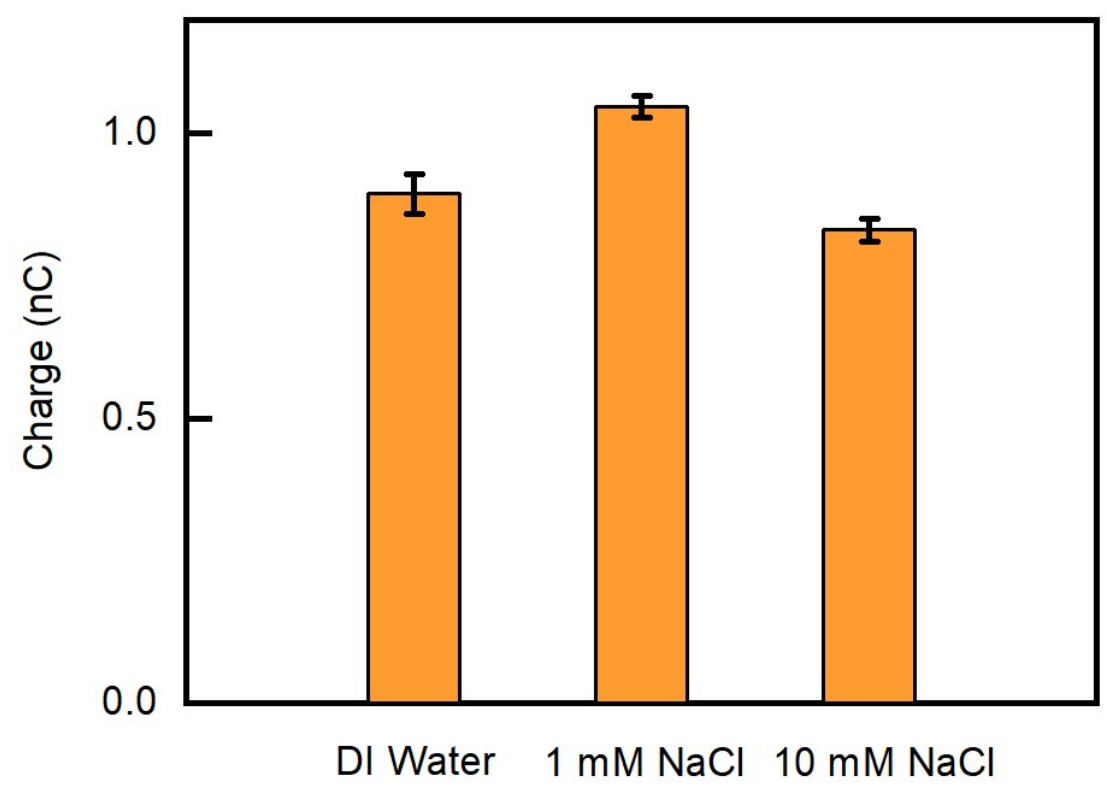

Fig. S4. Slide electrification effect of drops with different ion concentrations. We used 35 μL drops made from DI water, 1 mM NaCl solution, or 10 mM NaCl solution. The experiment was performed on a commercial FEP film surface tilted at 50°. The measured charge values results are averaged and we calculated the standard deviation of measurements with 20 drops, respectively.

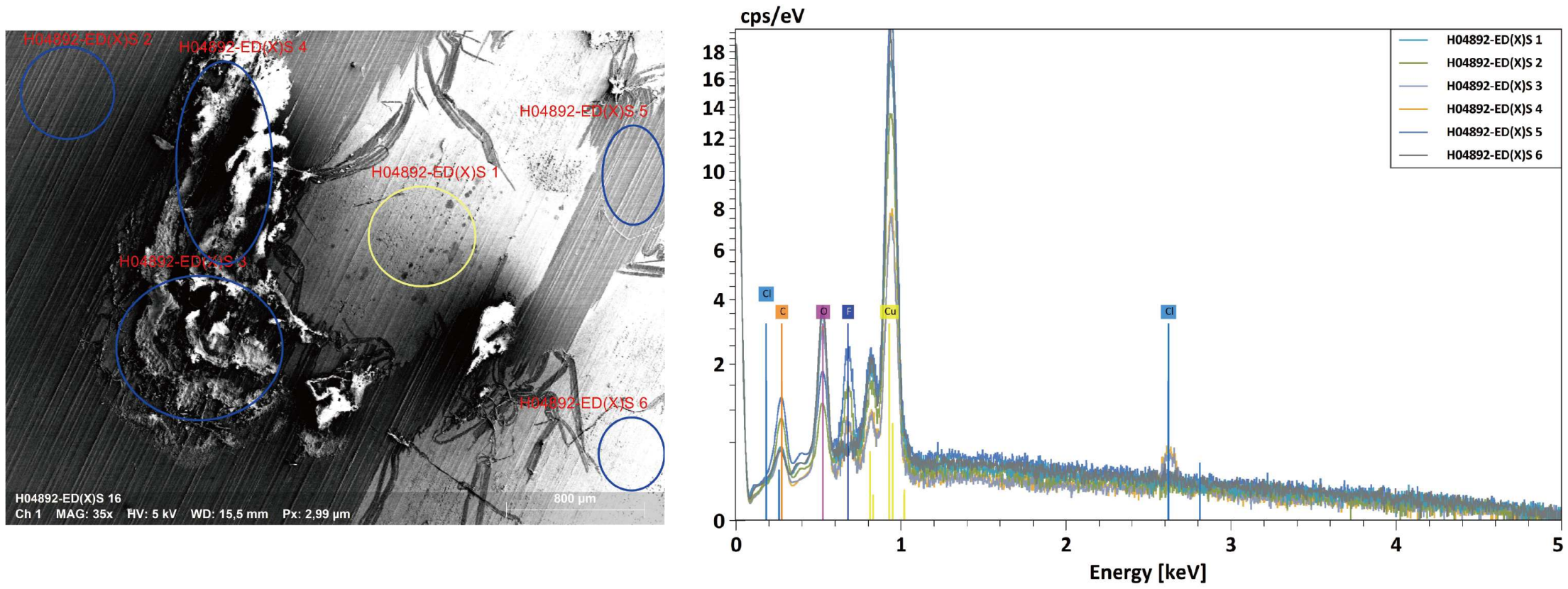

Position		Cu	O	F	Cl	C
(Element / Position)		Atom [%]				
Corroded	H04892-ED(X)S 1	70.6	26.6	0	0	2.8
Uncorroded	H04892-ED(X)S 2	68.7	8.8	9.4	0	13.1
Corroded	H04892-ED(X)S 3	43.9	34.2	5.8	8.7	7.4
Corroded	H04892-ED(X)S 4	43.6	34	5	9.8	7.6
Uncorroded	H04892-ED(X)S 5	69.5	8.9	10.1	0	11.5
Corroded	H04892-ED(X)S 6	71.7	26.9	0	0	1.4

Fig. S5. SEM-EDS measurements for corrosion area of Teflon-coated Cu foil after impact of 50000 charged drops (35 µL, 10 mM NaCl). Water drops were charged by sliding down commercial FEP films (~0.8 nC, Fig. S4). Compared with the uncorroded area, fluorine and carbon decreased significantly in the corroded area, which means Teflon film is locally removed. Oxygen and chlorine element increased significantly in the corroded area, which implies that the corrosion products are mainly oxides and chlorides.

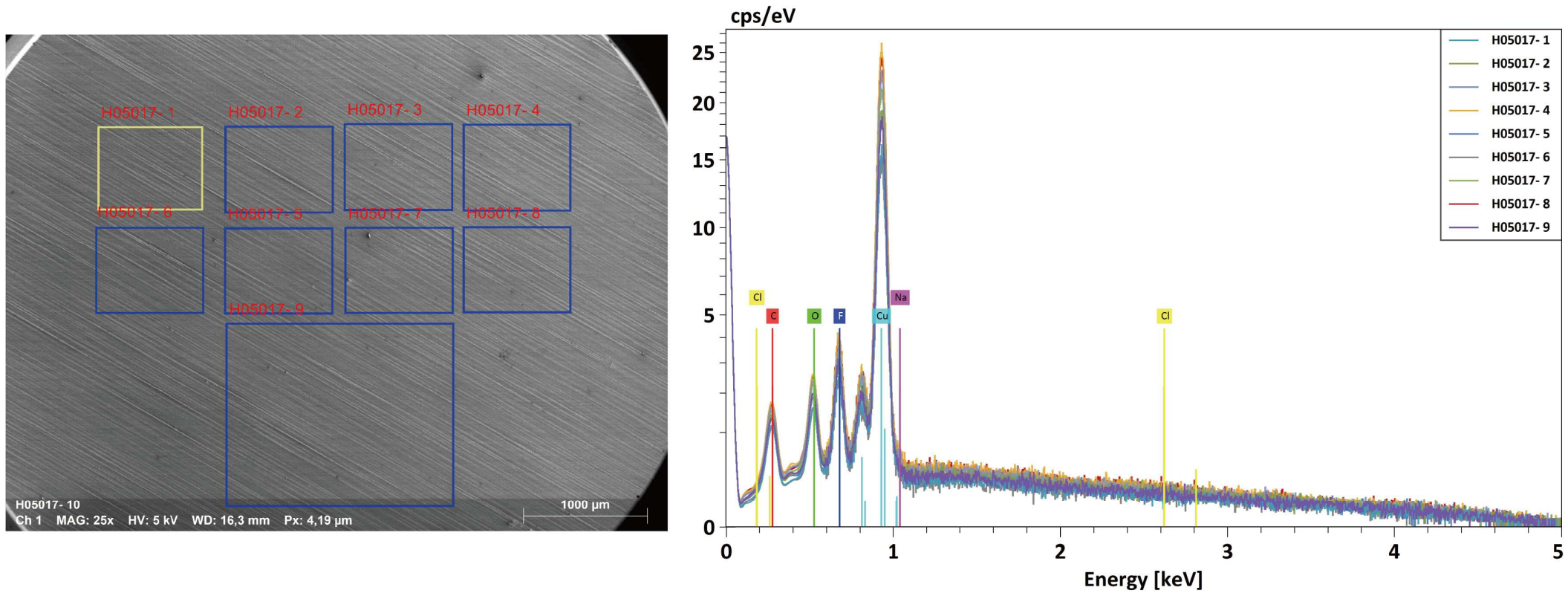

Element / Position	Atom [%]					
	Cu	O	F	Cl	C	Na
H05017-1	61.6	9.8	12.7	0	15.9	0
H05017-2	62.1	9.6	13.3	0.1	14.9	0
H05017-3	62.2	10.1	12.9	0.2	14.6	0
H05017-4	62.3	9.9	13.3	0	14.5	0
H05017-5	62.1	9.7	13.6	0	14.6	0
H05017-6	60.8	10	13.4	0	15.8	0
H05017-7	61.6	10.1	13.5	0	14.8	0
H05017-8	62.1	9.8	13.4	0.2	14.5	0
H05017-9	61.3	10.4	13.4	0.1	14.8	0

Fig. S6. SEM-EDS measurements of a Teflon-coated Cu foil after impact of 50000 neutral drops (35 µL, 10 mM NaCl). The drops were generated from the grounded needle and fall directly onto the Teflon-coated Cu foil surface. Compared with Fig. S5, no obvious damage patterns were observed in the surface morphology. Furthermore, EDS did not detect any elemental characteristics consistent with the abundant corrosion products.

a

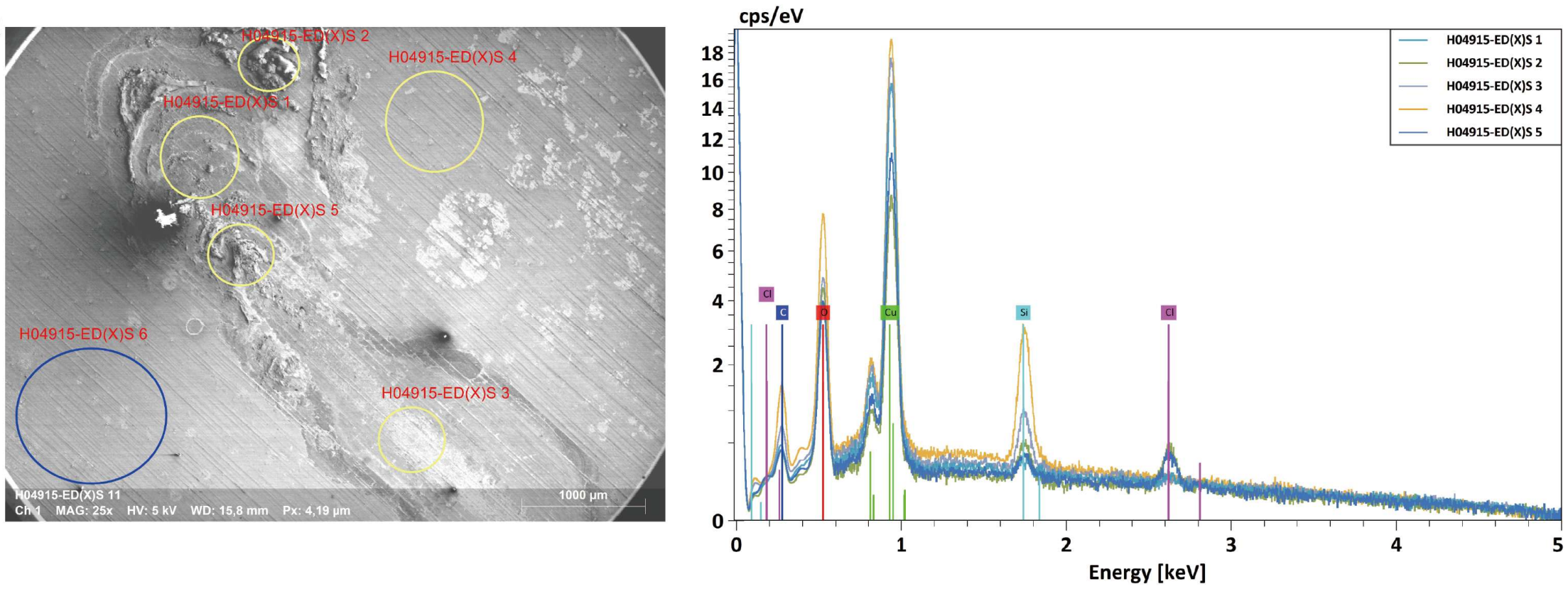

b

Position \ Element		Atom [%]				
		Cu	O	Si	Cl	C
Corroded	H04915-ED(X)S 1	65.2	29.2	1.3	0.8	3.5
Corroded	H04915-ED(X)S 2	41.4	41.3	6.1	7	4.2
Corroded	H04915-ED(X)S 3	58.5	30	5.8	0.2	5.5
Uncorroded	H04915-ED(X)S 4	43.2	33.1	14	0	9.7
Corroded	H04915-ED(X)S 5	51.6	36	3.4	5.4	3.6

c

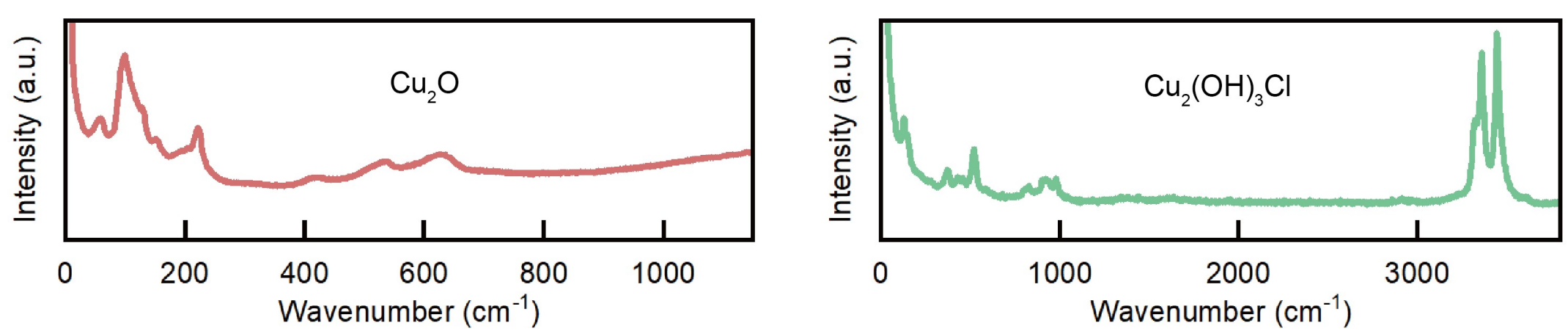

Fig. S7. SEM-EDS and Raman measurements of the corrosion area on a superhydrophobically coated Cu foil after impact of 50000 charged drops (35 µL, 10 mM NaCl). Water drops were charged by sliding down commercial FEP films. **(a-b)** SEM-EDS result. Compared with the uncorroded area, the amount of silicon decreased in the corroded area. This indicates that commercial superhydrophobic coating (main component is silica (SiO_2)) is destroyed. But, the oxygen did not decrease as the silicon, and even increased in some areas. At the same time, the chlorine increased in the corroded area, which shows that the corrosion products were mainly oxides and chlorides. **(c)** Raman result. The spectra of two substances were mainly detected: cuprous oxide and basic cupric chloride.

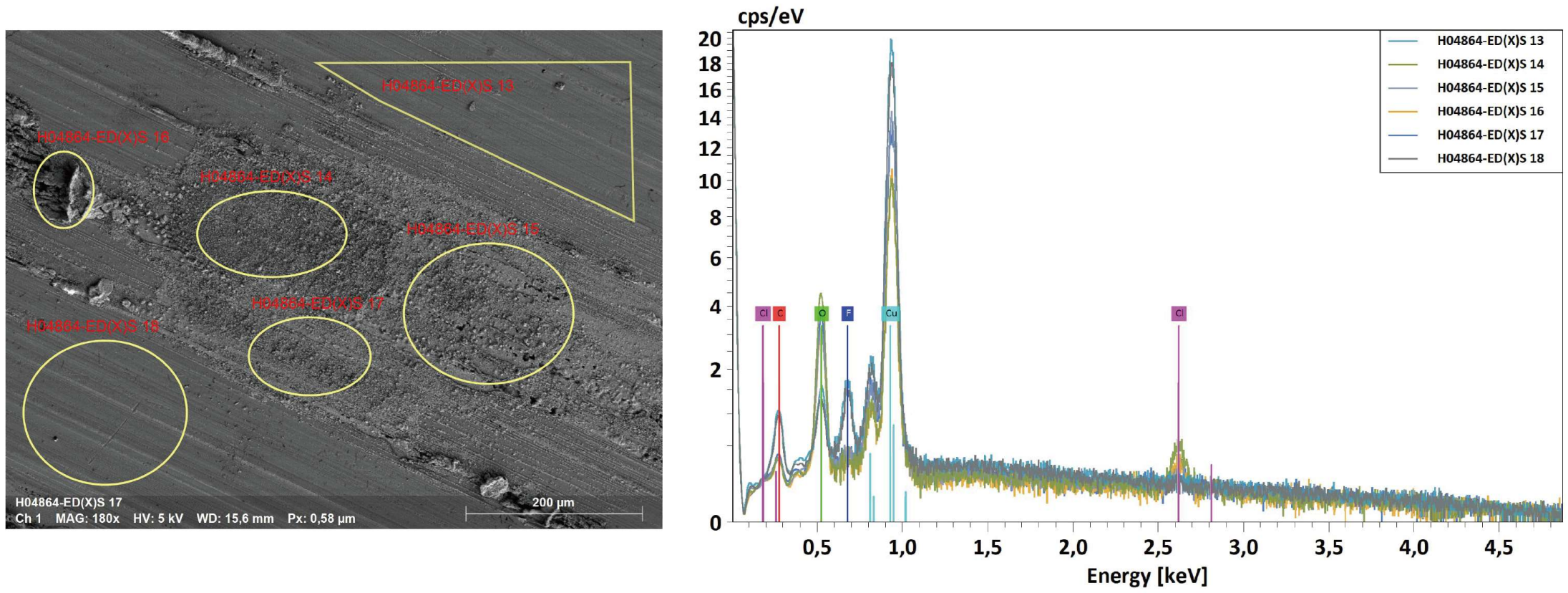

Element / Position		Atom [%]				
		Cu	O	F	Cl	C
Uncorroded	H04864-ED(X)S 13	74.3	8.5	7.6	0	9.7
Corroded	H04864-ED(X)S 14	47	40.7	0.3	9.8	2.1
Corroded	H04864-ED(X)S 15	68.7	28	0.7	1.2	1.5
Corroded	H04864-ED(X)S 16	57.3	33.1	1	6.1	2.5
Corroded	H04864-ED(X)S 17	61.3	31	0.7	4	3
Uncorroded	H04864-ED(X)S 18	74.7	7.3	7.2	0.2	10.6

Fig. S8. SEM-EDS measurements of a Teflon-coated Cu foil after impact of 10000 charged drops (35 µL, 10 mM NaCl). Water drops were charged by sliding down commercial FEP films. Fluorine and carbon decreased significantly in corroded areas as compared to uncorroded areas. This indicates that Teflon was locally removed. Oxygen and chlorine were increased in the corroded areas, showing that the corrosion products are mainly oxides and chlorides.

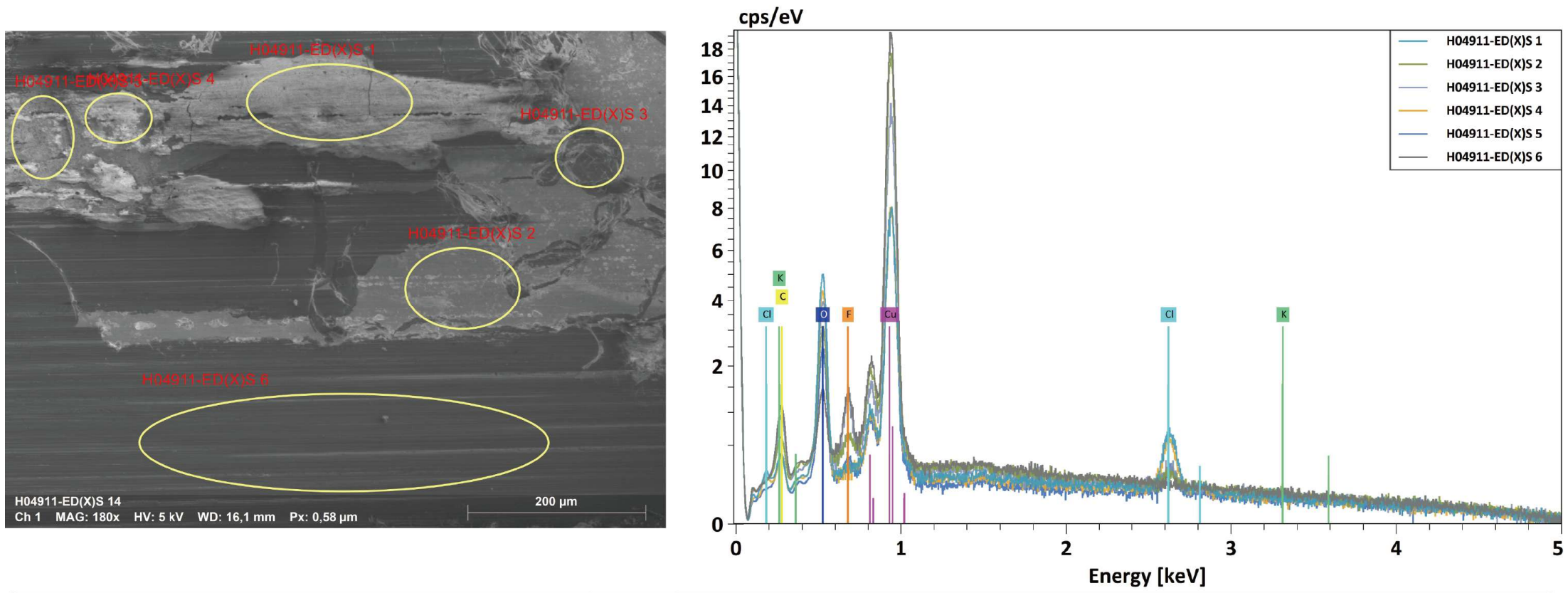

Position		Atom [%]				
	Element	Cu	O	F	Cl	C
Corroded	H04911-ED(X)S 1	37.4	45.5	0.2	13.8	2.9
Corroded	H04911-ED(X)S 2	69.9	22.4	1.6	1.8	4.2
Corroded	H04911-ED(X)S 3	53.8	28.6	6.2	2.8	8.5
Corroded	H04911-ED(X)S 4	41	42.8	0.1	12.5	3.5
Corroded	H04911-ED(X)S 5	55	31.8	1.5	5.7	5.6
Uncorroded	H04911-ED(X)S 6	75.2	7.9	6	0	10.5

Fig. S9. SEM-EDS measurements of a Teflon-coated Cu foil after impact of 10000 charged drops (35 µL, 10 mM KCl). Water drops were charged by sliding down commercial FEP films. Significant fluorine and carbon decreased significantly in most corroded areas as compared to uncorroded areas, indicating local Teflon removal. An increase in oxygen and chlorine in the corroded areas showed that the corrosion products are mainly oxides and chlorides.

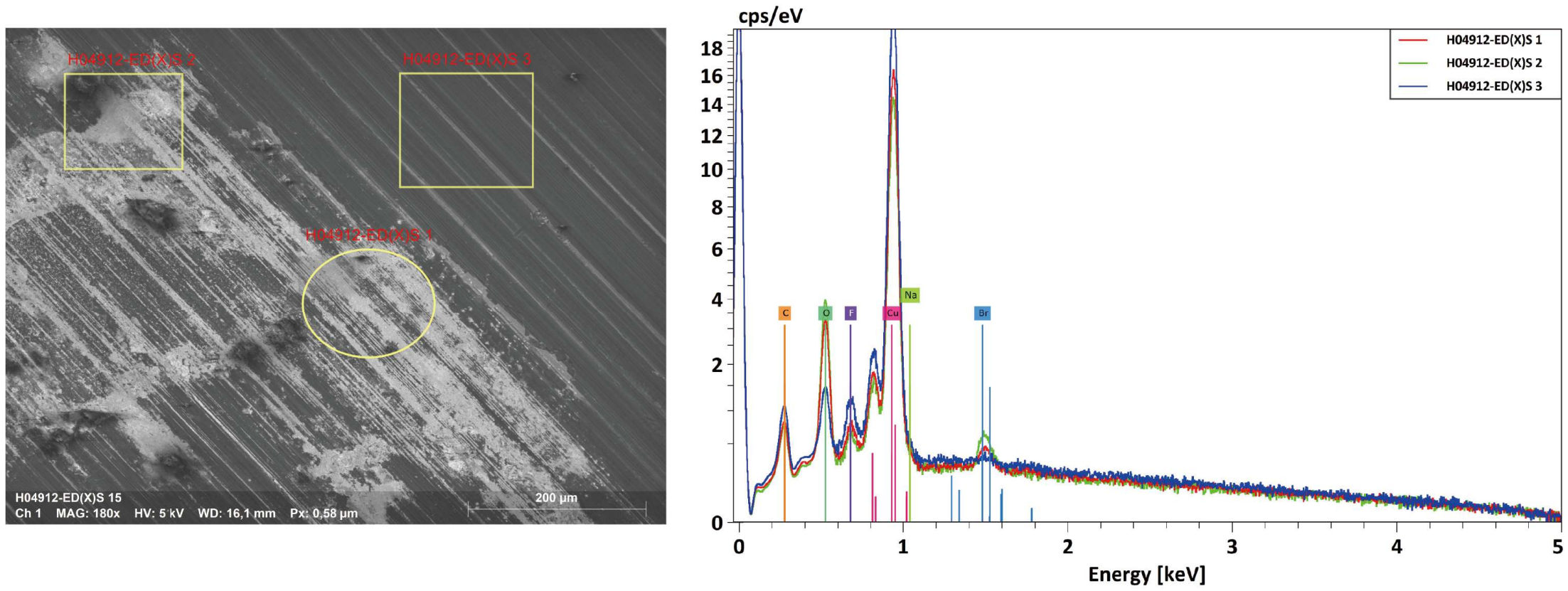

Position	Element	Atom [%]				
		Cu	O	F	Br	C
Corroded	H04912-ED(X)S 1	64.8	23.2	2.4	1.5	7.9
Corroded	H04912-ED(X)S 2	58.3	29.4	1.9	2.9	7.2
Uncorroded	H04912-ED(X)S 3	78	7.5	4.3	0.3	9.9

Fig. S10. SEM-EDS measurements of a Teflon-coated Cu foil after impact of 10000 charged drops (35 µL, 10 mM NaBr). Water drops were charged by sliding down commercial FEP films. Fluorine and carbon decreased in corroded areas as compared to uncorroded areas, which indicates that Teflon was removed. Oxygen and bromine were increased in the corroded areas, showing that the corrosion products are mainly oxides and bromides.

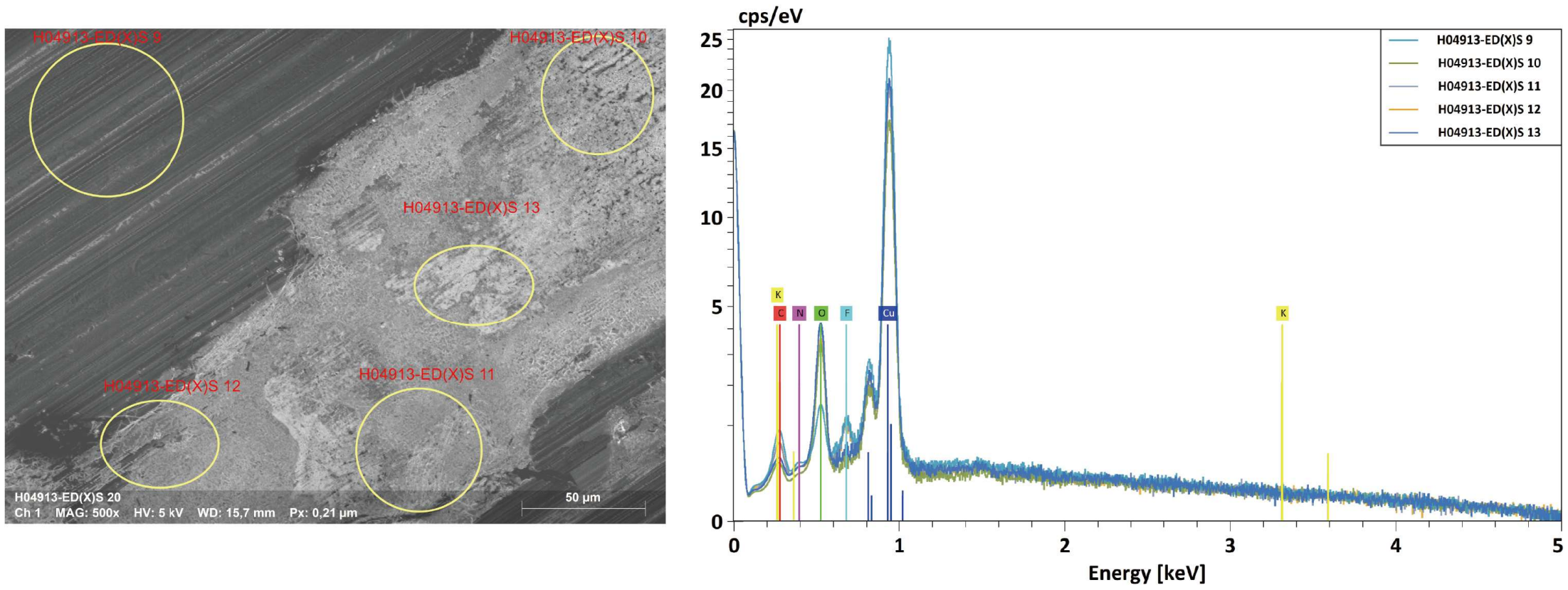

Position		Cu	O	F	N	C
Element		Atom [%]				
Uncorroded	H04913-ED(X)S 9	81.3	7.2	3.4	0.2	7.7
Corroded	H04913-ED(X)S 10	71	26.4	0.3	0.1	2.2
Corroded	H04913-ED(X)S 11	65.6	25.9	3.4	0	4.7
Corroded	H04913-ED(X)S 12	67.9	23	3.4	0	5.5
Corroded	H04913-ED(X)S 13	70.4	27.4	0.5	0	1.7

Fig. S11. SEM-EDS measurements of a Teflon-coated Cu foil after impact of 10000 charged drops (35 µL, 10 mM KNO_3). Water drops were charged by sliding down commercial FEP films. Fluorine and carbon decreased in some corroded areas, which indicates that Teflon was removed. Oxygen was increased in the corroded areas, showing that the corrosion products are mainly oxides. No effect of nitrogen was observed.

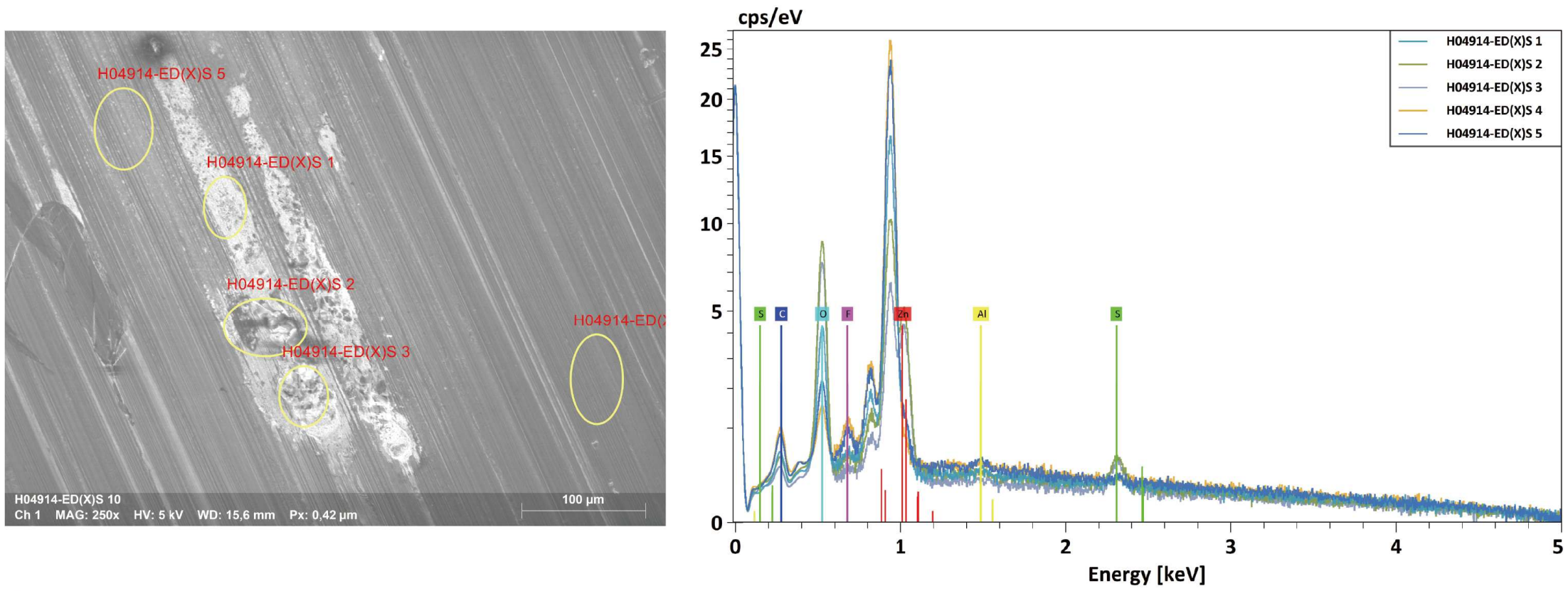

Position \ Element		Atom [%]					
		Cu	O	F	S	C	Zn
Corroded	H04914-ED(X)S 1	60.8	29	1.5	0.6	3.6	4.2
Corroded	H04914-ED(X)S 2	31	51	0.8	2.7	1.9	12.4
Corroded	H04914-ED(X)S 3	23.7	57.7	0.6	3	1.3	13.6
Uncorroded	H04914-ED(X)S 4	82.6	6.5	3	0	7.8	0
Corroded	H04914-ED(X)S 5	74.5	11.1	2.6	0.4	6.9	3.5

Fig. S12. SEM-EDS measurements of a Teflon-coated Cu foil after impact of 10000 charged drops (35 µL, 10 mM $ZnSO_4$) impact. Water drops were charged by sliding down commercial FEP films. Fluorine and carbon decreased in the corroded areas as compared to uncorroded areas, which indicates that Teflon is removed. Oxygen and zinc were increased significantly in the corroded areas, sulfur increased slightly showing that the corrosion products may some compounds related to zinc and oxides.

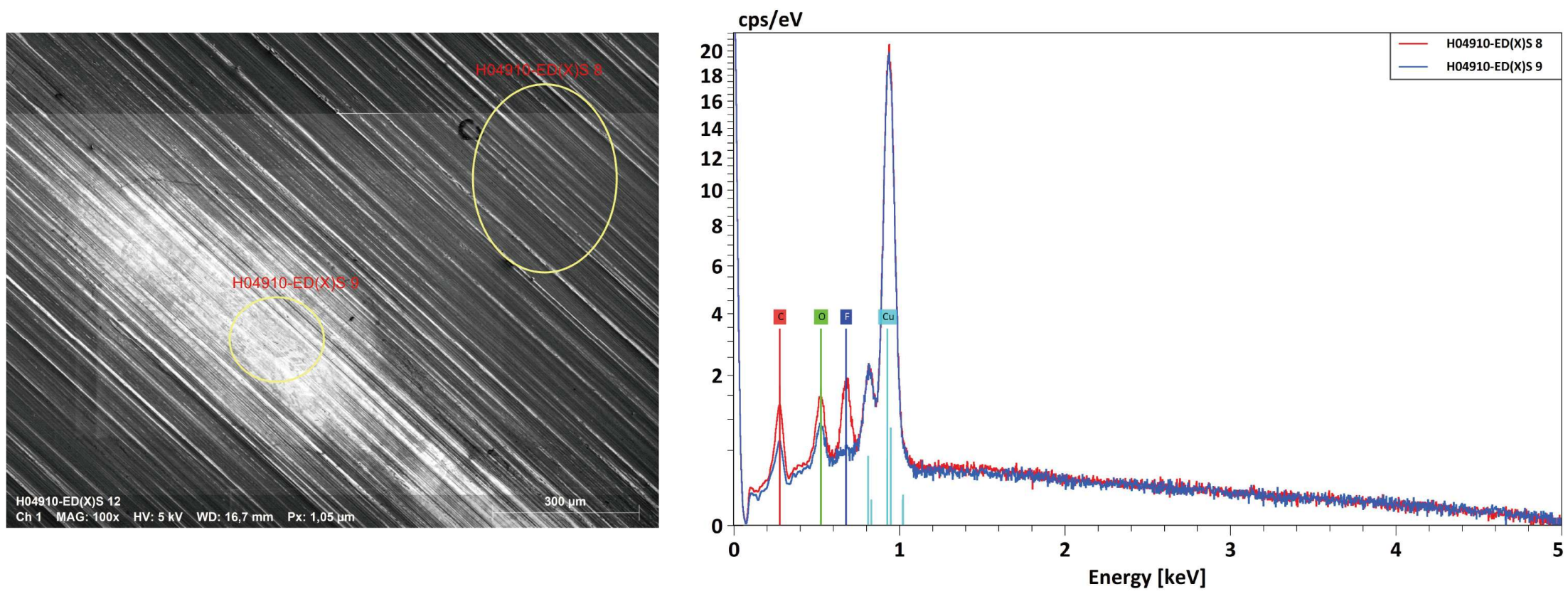

Position \ Element		Atom [%]			
		Cu	O	F	C
Uncorroded	H04910-ED(X)S 8	73.1	7.4	8.4	11.1
Corroded	H04910-ED(X)S 9	89	4.8	0.7	5.5

Fig. S13. SEM-EDS measurements of a Teflon-coated Cu foil after impact of 10000 charged drops (35 µL, deionized water). Water drops were charged by sliding down commercial FEP films. Fluorine and carbon decreased in corroded areas as compared to uncorroded areas, which indicates that Teflon was locally removed. Oxygen did not increase as in the other cases. Thus, deionized water showed a reduced corrosion. But deionized water is rarely encountered in natural environments.

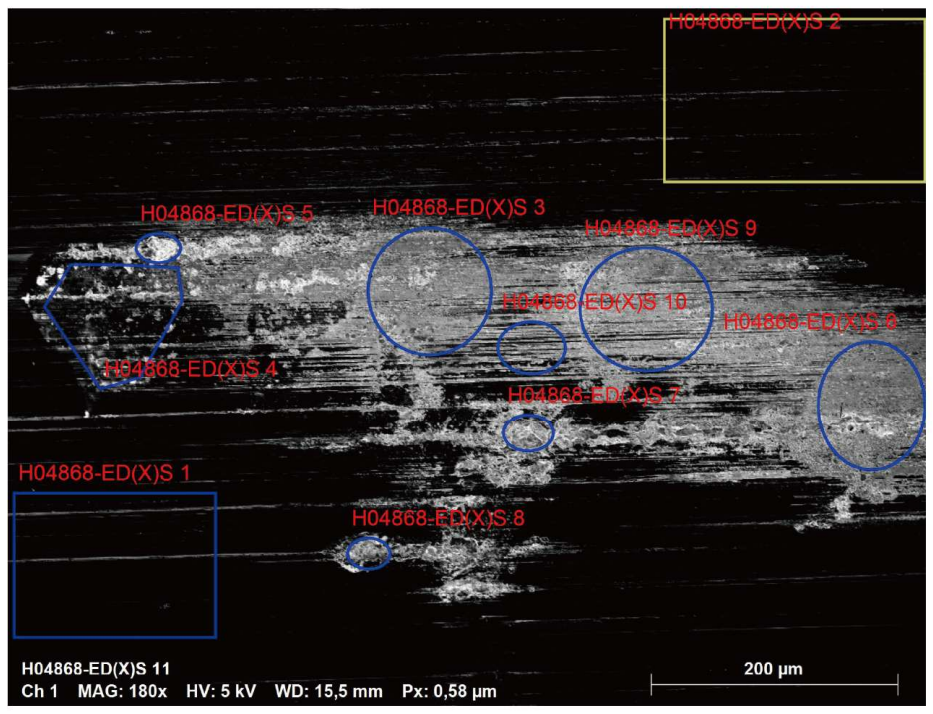

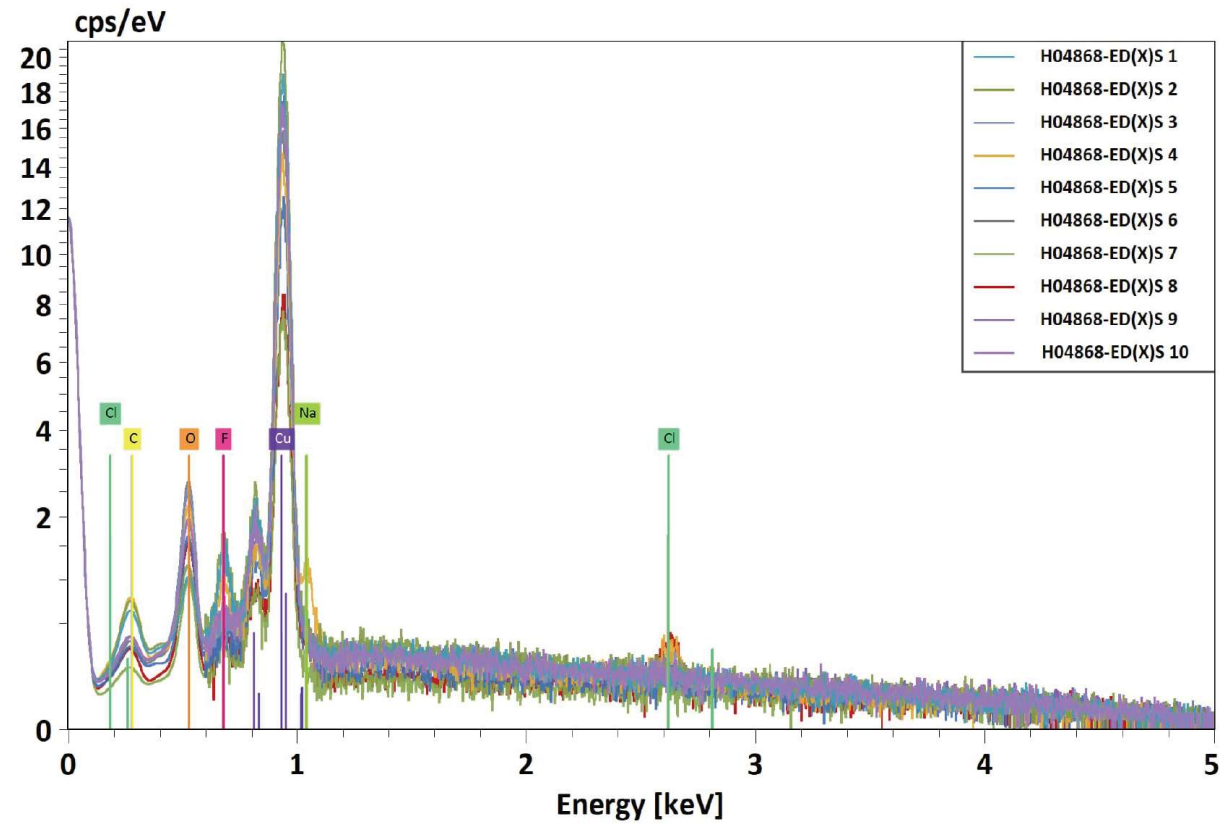

Position		Element: Atom [%] Cu	O	F	Cl	C
Uncorroded	H04868-ED(X)S 1	77.6	6.7	6.6	0	9.1
Uncorroded	H04868-ED(X)S 2	76.1	6.7	6.7	0.1	10.4
Corroded	H04868-ED(X)S 3	71.9	22.5	0.8	0.9	3.9
Corroded	H04868-ED(X)S 4	54.5	17.7	4.5	3.9	13.6
Corroded	H04868-ED(X)S 5	70	19.7	2	2.2	4.5
Corroded	H04868-ED(X)S 6	68.5	24.8	1.6	1.3	3.8
Corroded	H04868-ED(X)S 7	72.7	19.3	3.3	1.5	3.2
Corroded	H04868-ED(X)S 8	56.5	24.1	1.3	10.6	7.5
Corroded	H04868-ED(X)S 9	72.7	21.2	1.4	0.7	4
Corroded	H04868-ED(X)S 10	76.9	17.2	2.1	0.3	3.5

Fig. S14. SEM-EDS measurements of a Teflon-coated Cu foil after impact of 10000 charged drops (35 µL, 10 mM NaCl). Water drops were charged by sliding down commercial FEP films. The experiment was conducted in an oxygen-free environment and the solution was also degassed to remove dissolved oxygen. Fluorine and carbon decreased in corroded areas as compared to uncorroded areas, which indicates that Teflon is removed. Oxygen still increased but not as much as in a normal environment. Chloride increased significantly. It shows that although the corrosion effect of spontaneously charged drops is affected by oxygen environment, it does not play a decisive role.

Discussion 3: Surface blistering on aluminum induced by charged drops

Contents: supporting text and Fig. S15-S16

We prepared a 100 nm thick aluminium layer on glass slides by vacuum thermal evaporation. The aluminium surface was then coated with either a 60 nm thick Teflon film by dip-coating or a PFOTS molecular layer by chemical vapour deposition. The aluminum surface is hydrophobic after coated Teflon film or PFOTS. Hydrophobic surfaces help impacting water drops to run off the surface after impact. Unlike the surface defects observed on copper substrates, bubble-like structures were observed on the aluminium surface under confocal microscopy after impact of 3000 charged drops (Fig. S15 and S16). These structures showed almost no obvious change after heating to 80°C for 0.5 h, indicating that they were not bubbles supported by residual liquid water or volatile water pockets. The bubble-like structures were more likely fixed interfacial damage or corrosion features.

We hypothesize that these bubble-like structures originated from charged-drop-induced local dielectric breakdown and subsequent aluminium corrosion. Dielectric breakdown locally damaged the coating, either the Teflon film or the PFOTS molecular layer, as well as the Al_2O_3 passivation layer. Water could therefore directly contact the underlying metallic Al and induce local oxidation and hydration reactions. This process can be described by the simplified reaction:

$$2\,Al\ + 6\,H_2O \rightarrow 2Al(OH)_3 + 3H_2\uparrow$$

The aluminium–water reaction is thermodynamically feasible, but it is normally inhibited by the dense and well-adhered aluminium oxide layer on the Al surface. Therefore, dielectric breakdown induced by charged droplets could locally disable the protective layer and allow aluminium to react with water. The internal formation of hydrated oxidation products and local H_2 generation may further causes the surface to blister[6,7].

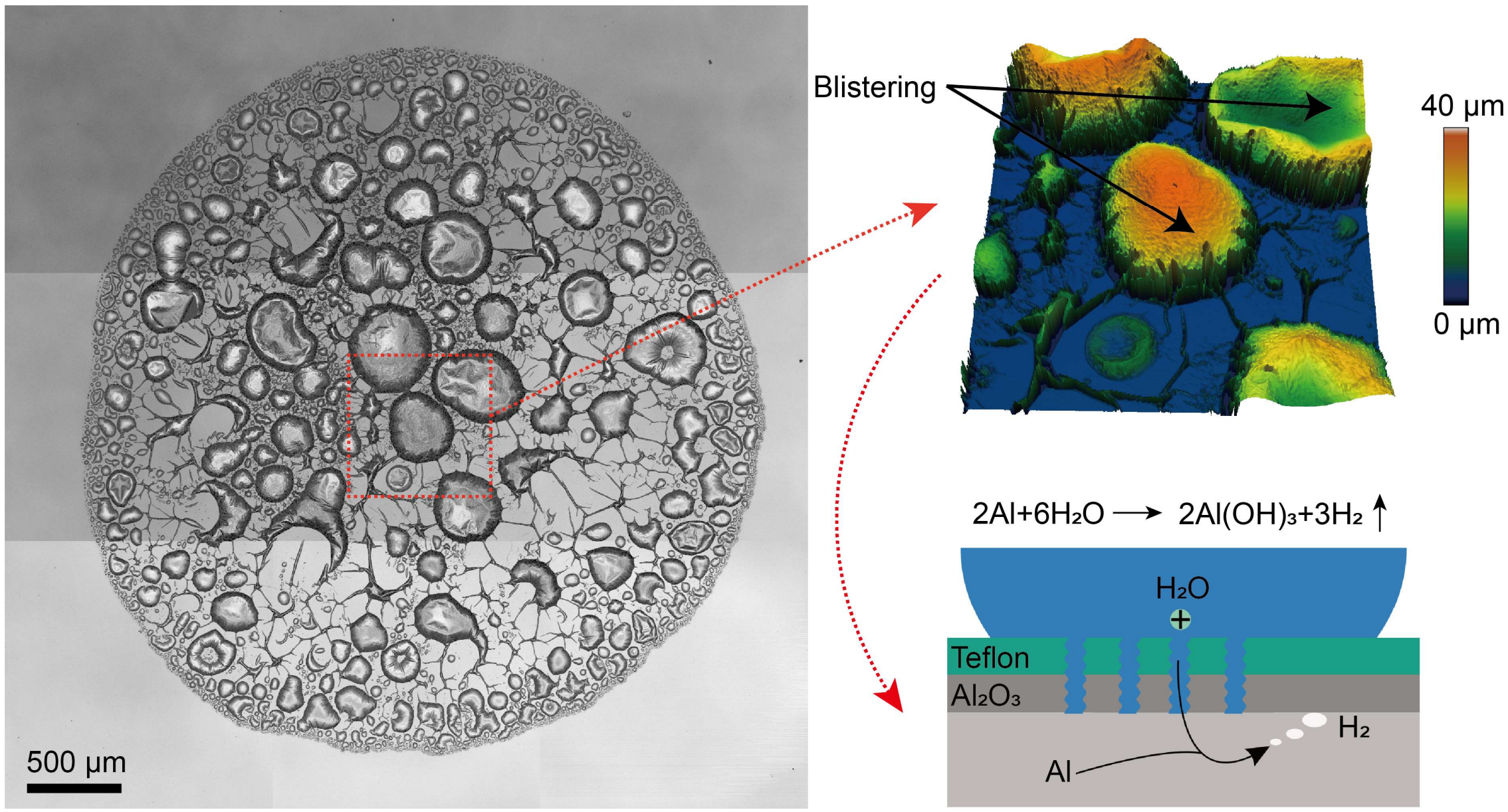

Fig. S15. Teflon-coated aluminium surface after the impact of 3000 charged water drops (35 µL, 1 mM NaCl). Reflection mode confocal microscopy 2D and 3D images of Teflon-coated aluminium after impact of 3000 charged water drops, which had run down a PFOTS-on-quartz plate. Surface blistering was observed.

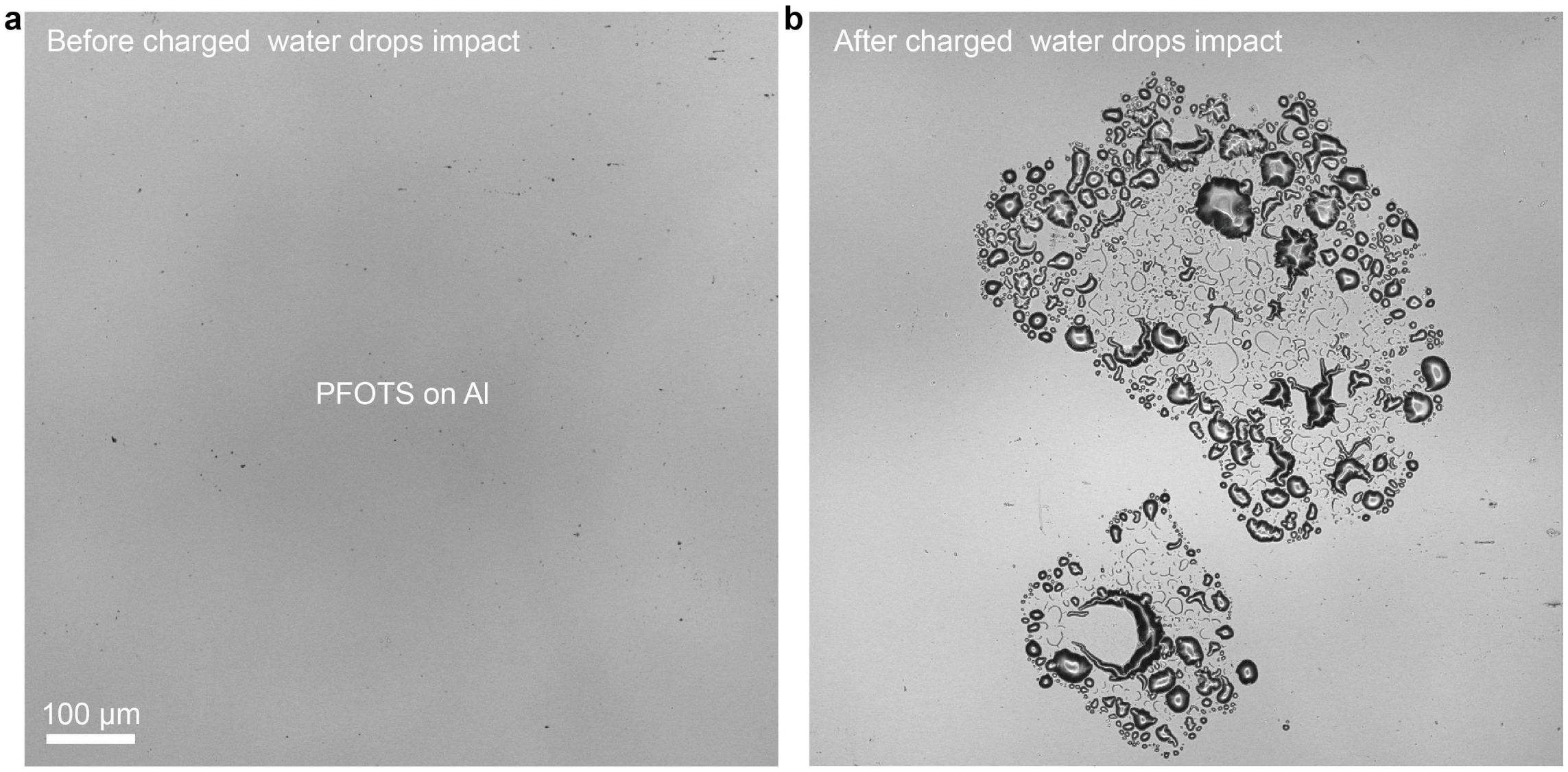

Fig. S16. Reflection confocal microscopy images of naturally oxidized aluminum coated with a PFOTS monolayer before (a) and after (b) contact with 1000 charged water drops (35 μL, 1 mM NaCl). Water drops had run down a PFOTS-on-quartz plate. Surface blistering was observed after the impact of charged water drops.

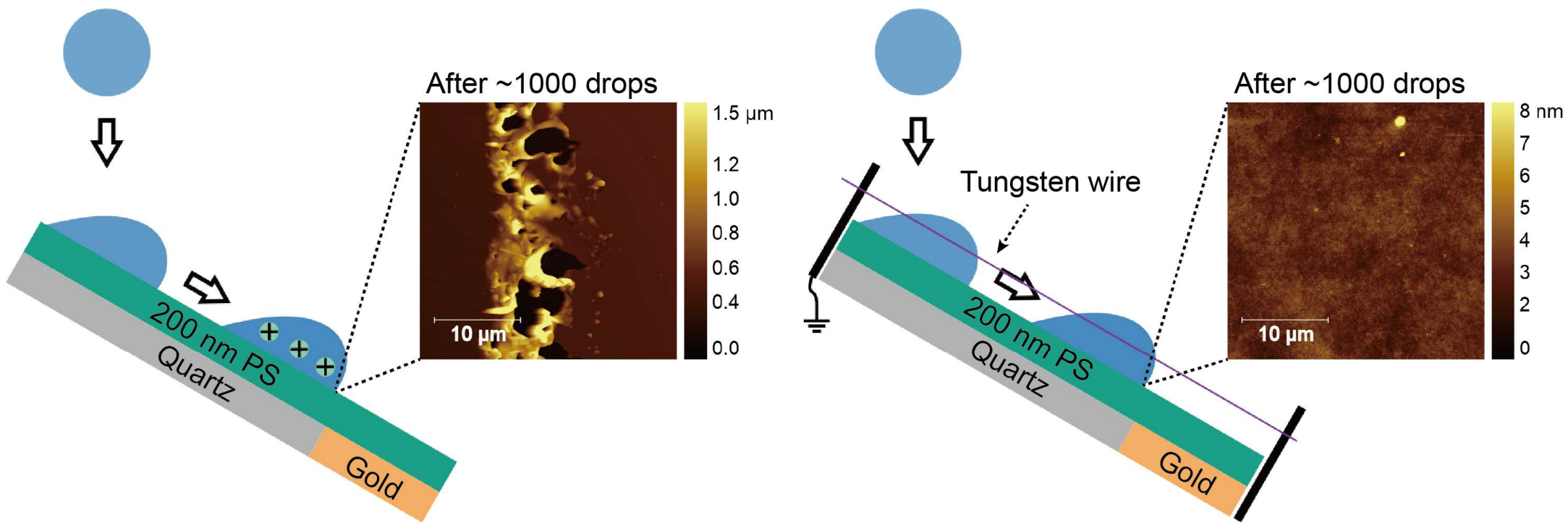

Fig. S17. Comparison of damage of charged and electrically neutral drops (ground by a tungsten wire). By connecting the drop with thin tungsten wire, 25 µm diameter, spanned at ≈1 mm height above the surface, charging of the drops can be suppressed without affecting their movement. After 1000 charged drops (35 µL, 1mM NaCl) sliding along the surface, we observed surface damage close to the quartz-copper boundary area. The same experiment with electrically neutral drops results in no surface damage, similar to the surface before sliding.

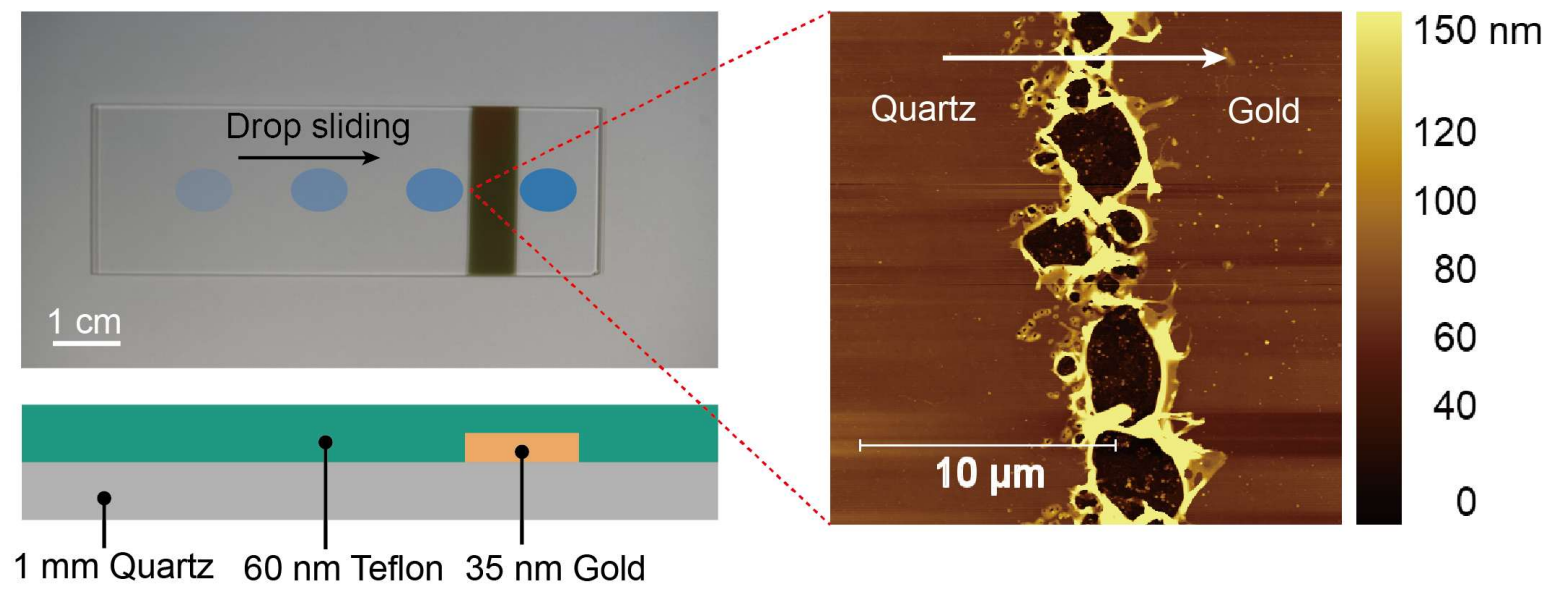

Fig. S18. Example of the spontaneous charged drop corrosion effect in a composite sample. First, a 35 nm gold layer was sputtered onto a small area on the surface of the quartz plate (75×25×1 mm). Then the entire surface was covered by a 60 nm Teflon film. The gold layer is isolated and not grounded. Despite the fact that only a small amount of gold was buried under the coating, the spontaneously charged drops (~1000, 35 µL, 1 mM NaCl) still disrupted the surface at the quartz-gold boundary area.

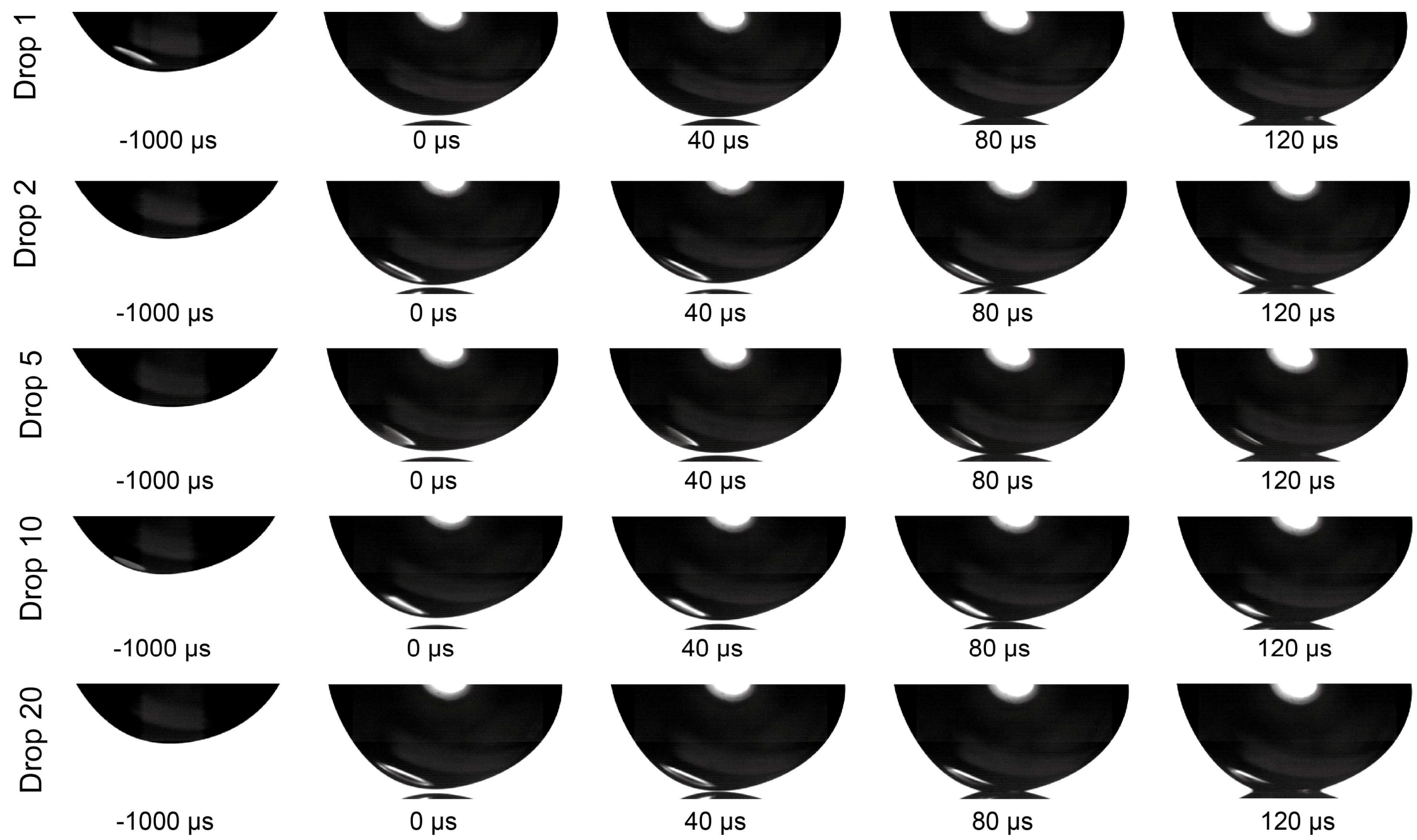

Fig. S19. High speed video camera images of electrically neutral drops as they approach a Teflon-coated copper surface. The drops (35 µL, 1mM NaCl) are generated from the grounded needle and fall (~4 cm) directly onto the Teflon-coated copper surface.

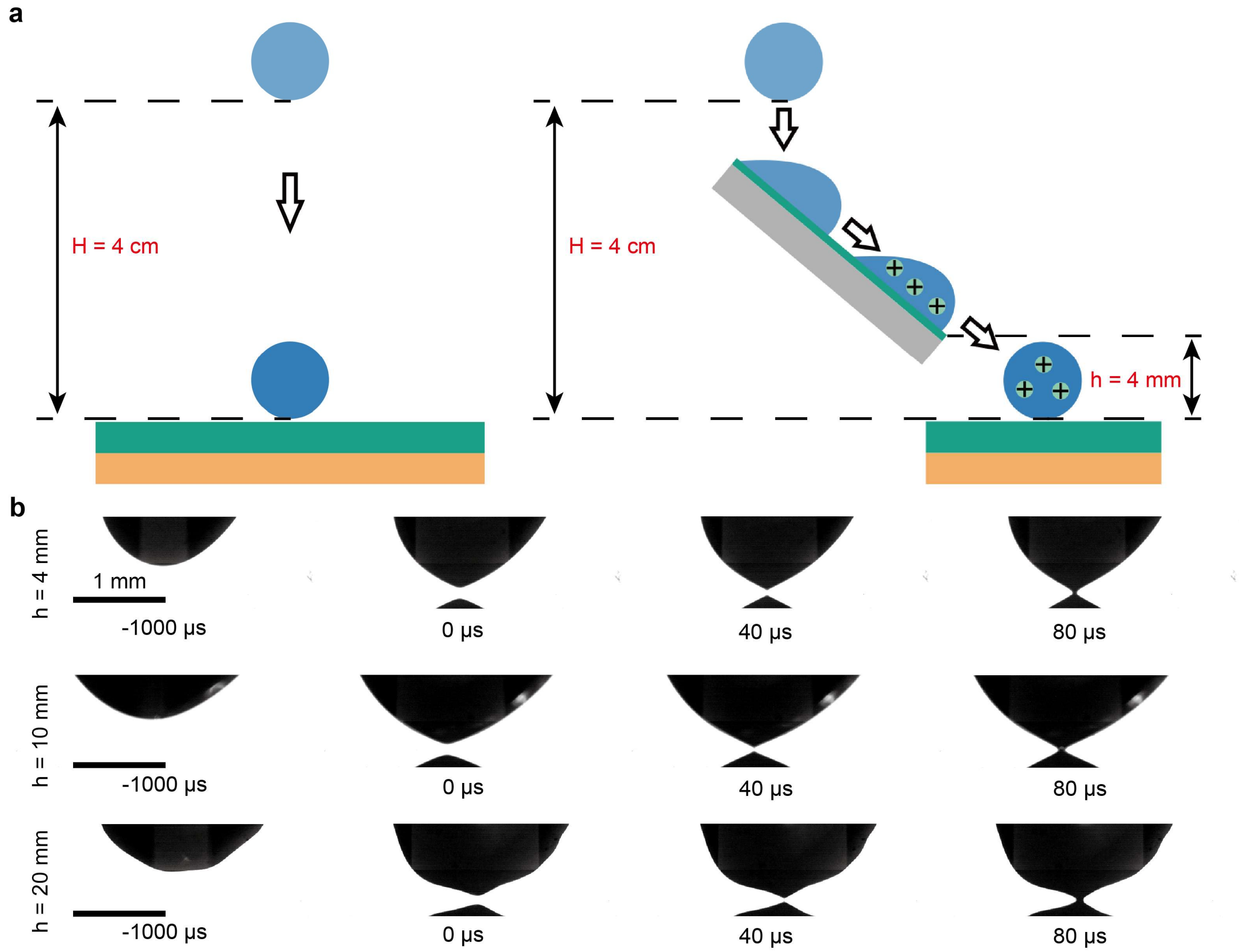

Fig. S20. The effect of the falling height of charged drops on discharge. (a) H is the height of the drop from leaving the needle to hitting the sample surface, and h is the height from the bottom of the tilted surface to the sample surface. **(b)** A commercial FEP film was used for slide electrification of 35 μL, 1 mM NaCl water drops. We varied the height from the bottom of the tilted surface to the sample surface by h = 4 mm, 10 mm, 20 mm. For all heights, the drop discharged as it approached the sample surface, although at higher heights the drop shape oscillated.

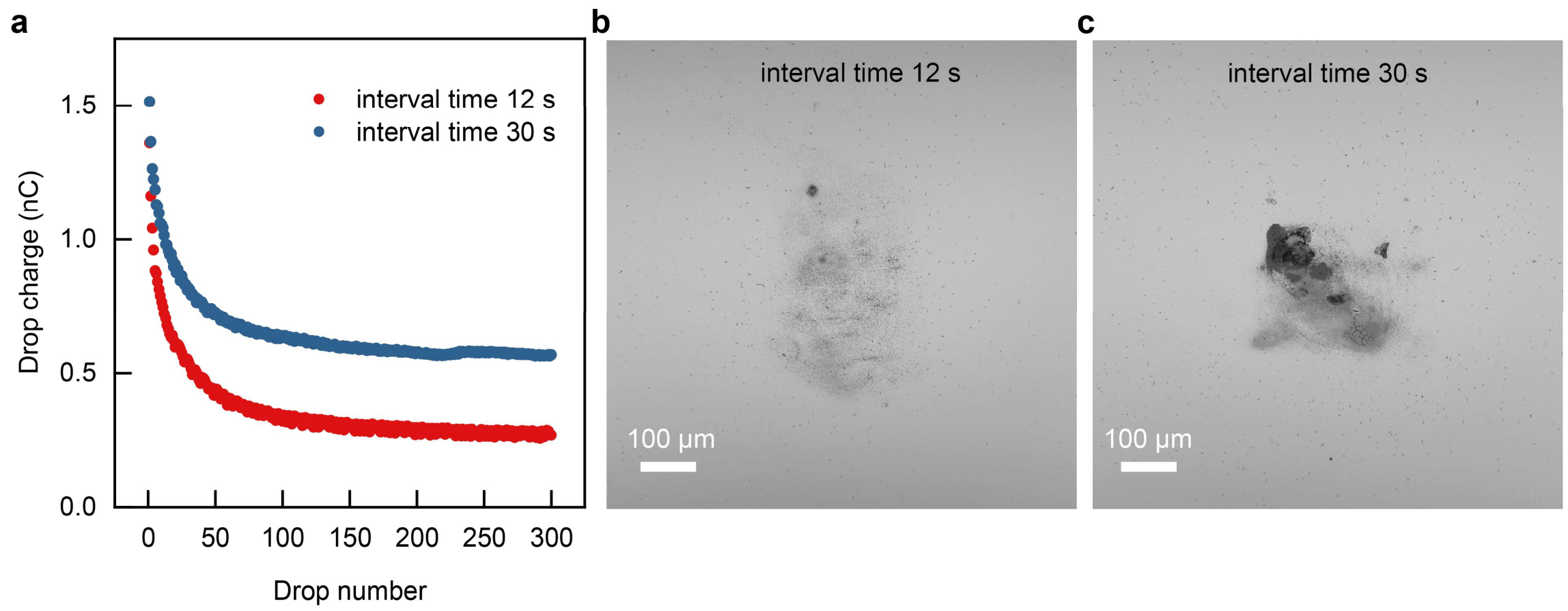

Fig. S21. Charged drop impact experiment without ionizing air blower. Water drops (35 µL, 1 mM NaCl) sliding down a PFOTS-coated glass and then impacting on a Teflon-coated Cu sample. The interval time between the drops were set to 12s and to 30s. **(a)** Charge of a drop versus the drop number. In both cases, the drop charge reached a stable value after 200 drops. **(b)** and **(c)** Surface morphology after 3000 charged drop impacted at the 12 s and 30 s interval condition.

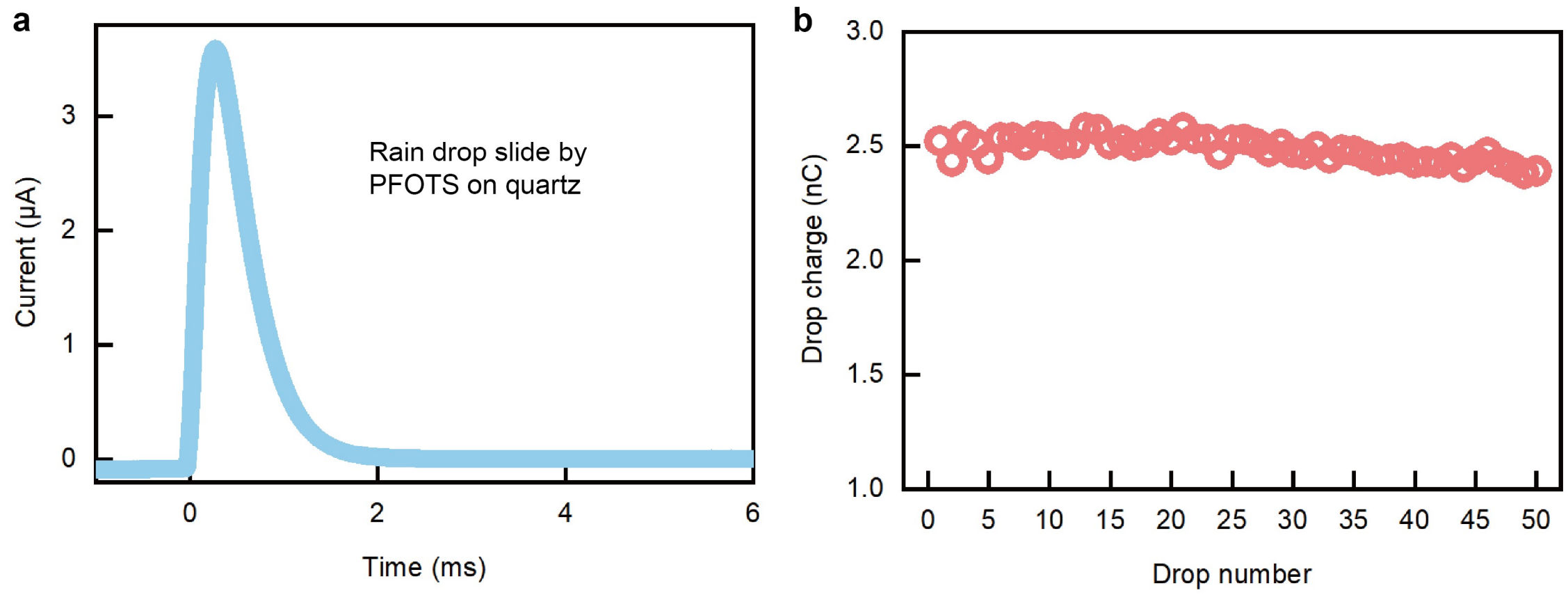

Fig. S22. Slide electrification of rainwater drops. Rainwater was collected directly from the Mainz area using glass bottles and a funnel and then used for experiments. **(a)** Measured current signals for single raindrop sliding on PFOTS on quartz. **(b)** The amount of charge generated by sliding electrification of 50 raindrops (35 μL, interval time 12 s) was measured continuously.

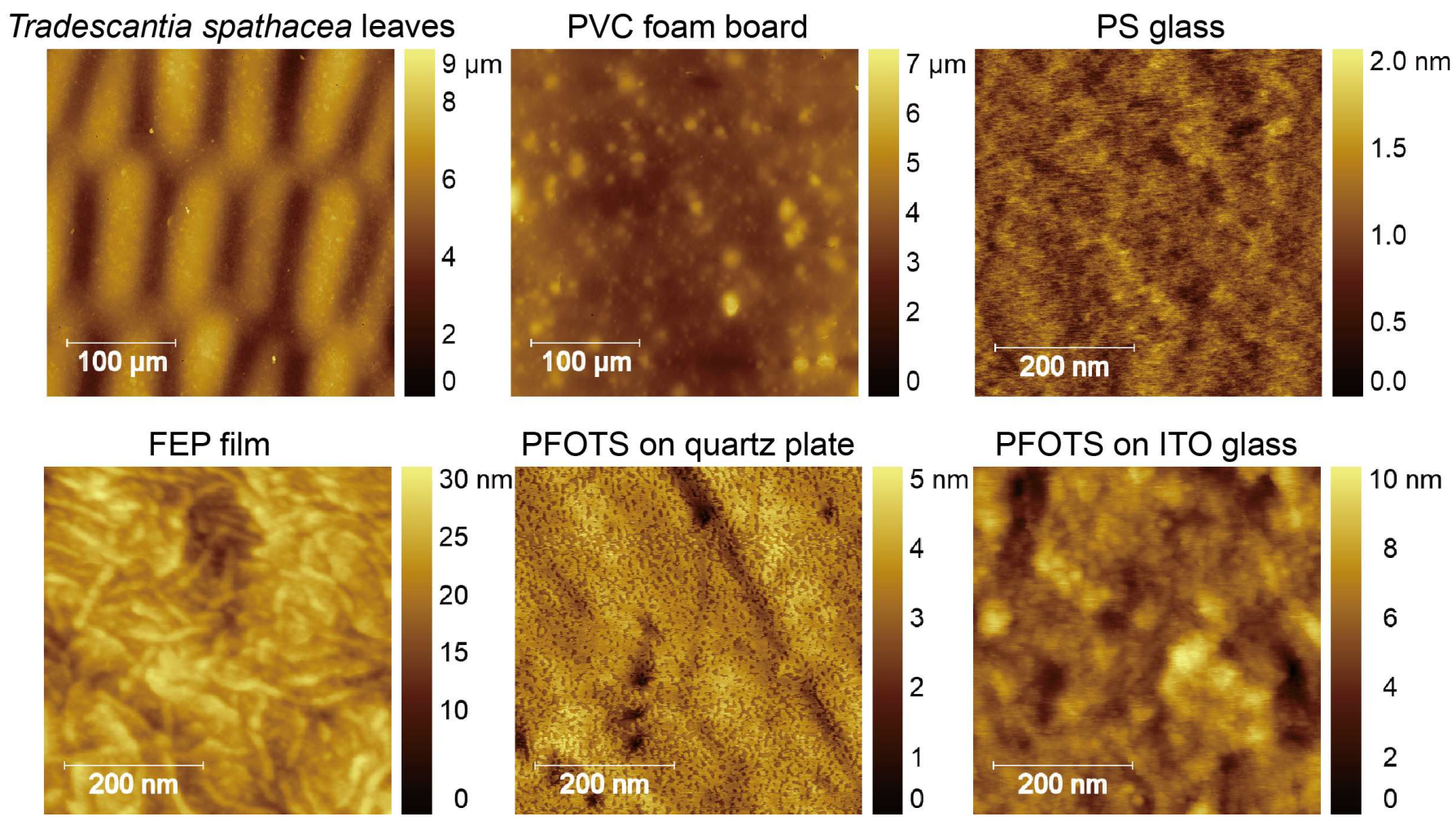

Fig. S23. Surface topography of samples used for slide electrification of drops. Images of *Tradescantia spathacea* leaves and PVC foam boards were obtained using confocal microscope (confocal white light microscope, NanoFocus µsurf, NanoFocus AG). PS glass, FEP film, PFOTS on quartz plate and PFOTS on ITO glass only showed features on the sub-µm scale. For this reason, these surfaces were imaged using AFM (Dimension Icon, Bruker).

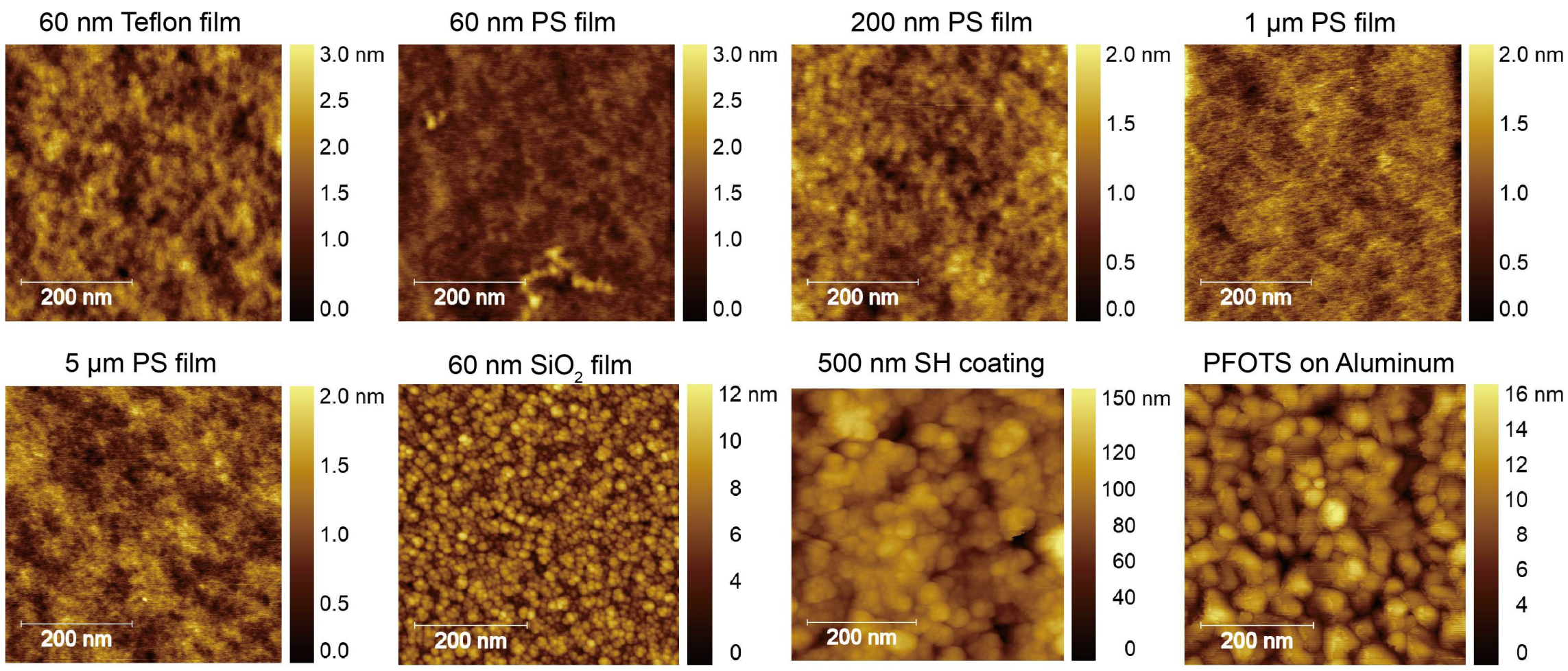

Fig. S24. Surface topography of coatings imaged by AFM (Dimension Icon, Bruker).

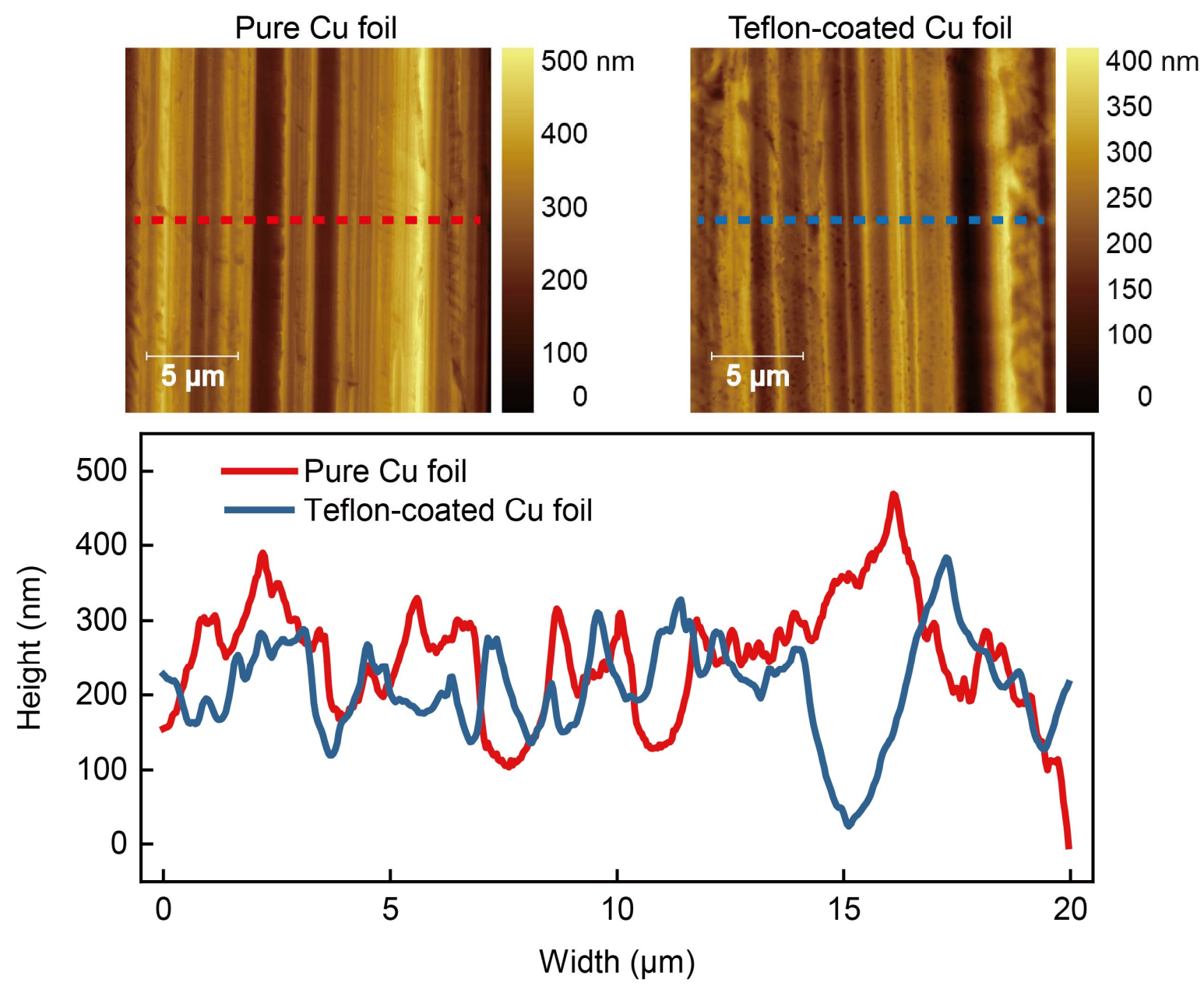

Fig. S25. Surface topography of a commercial Cu foil before and after Teflon coating. Surfaces were imaged by AFM (Dimension Icon, Bruker). The corresponding height profiles were extracted along the dashed lines in the AFM images. Commercial Cu foil has a rough surface with undulations of 200-500 nm in height. After coating with a Teflon film, the undulation amplitude is slightly reduced but remains visible.

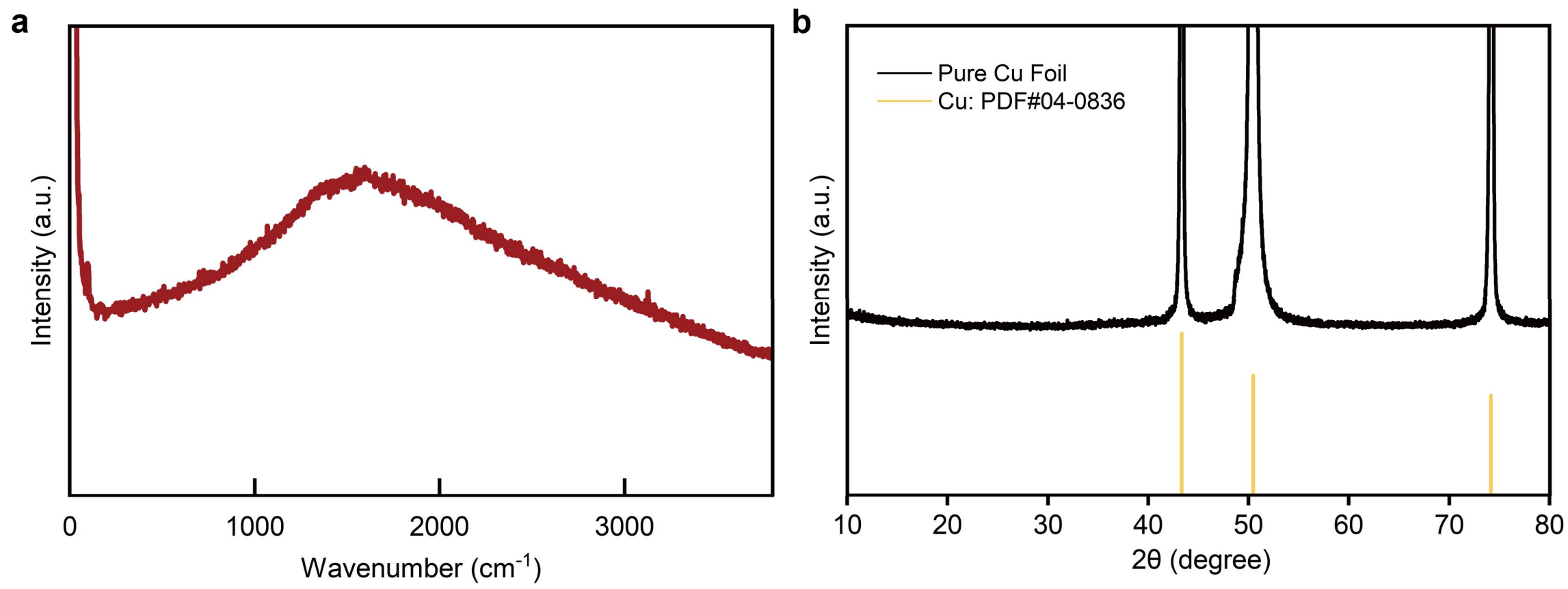

Fig. S26. Raman and X-ray diffraction results of pristine copper foil. (a) Raman result show no distinct vibrational modes characteristic for specific chemical bonds. **(b)** XRD results predominantly exhibited the characteristic diffraction peaks of copper.

Discussion 4: Electrochemical impedance spectroscopy of sputtered Cu samples with coatings

Contents: supporting text, Fig. S27, and Table S2

To better connect to prior knowledge of corrosion, we performed EIS measurements with polystyrene and Teflon coated samples prepared on sputtered Cu layers. We used a 3.5 wt% NaCl aqueous solution (~600 mM), which is a commonly used electrolyte in corrosion studies. From the Nyquist plots in Fig. S27a, it can be seen that the pristine bare Cu sample exhibits the smallest impedance arc, with a magnitude in the 1 kΩ range. After coating the Cu with a 60 nm PS film, the impedance arc increased to the 100 kΩ range, indicating that the polymer film markedly increased the interfacial impedance. For the 60 nm Teflon film, the impedance arc was even larger than that of the PS film with the same thickness. As the thickness of the PS film further increases, the impedance arc continues to enlarge, indicating that increasing film thickness further enhances the interfacial resistance. Fig. S27b shows the corresponding Bode modulus plots, which provide a comparison of the impedance differences among the samples. The impedance modulus in the low-frequency region mainly reflects the barrier performance and corrosion resistance of the coating. At low frequencies, the coated samples exhibit significantly higher impedance than pristine Cu. The impedance increases with increasing film thickness. Fig. S27c shows the Bode phase angle plots. Compared with pristine Cu, the coated samples show higher and broader phase angle peaks, indicating a stronger capacitive response. This suggests that the coatings provide a more effective barrier against charge transfer and electrolyte penetration.

To further understand the corrosion-related characteristics, we also fitted the EIS data using equivalent circuit models. The corresponding models are shown as the insets in the Nyquist plots in Fig. S27a. In these circuits, R_1 represents the solution resistance, R_2 the charge transfer resistance, R_3 the coating resistance. C_2 and C_3 represent the double layer capacitance and coating capacitance. Since the samples do not behave as ideal parallel-plate electrodes, all the capacitance-related elements are expressed as constant phase elements (CPEs). The fitted values of several representative circuit elements are summarized in Table S2. Among them, R_2 and R_3 are key parameters reflecting the corrosion protection performance of the samples. Both R_2 and R_3 increase continuously with increasing film thickness, indicating that the coating enhances the resistance of the system to charge transfer and ion penetration. In particular, R_2 increases from 6 kΩ for pristine Cu to 60 MΩ for the 5 μm PS coating, corresponding to an increase of nearly 4 orders of magnitude.

Taken together, the Nyquist plots, Bode plots, and equivalent-circuit fitting results consistently demonstrate that the developed coatings can significantly increase the interfacial resistance and effectively suppress interfacial electrochemical processes, indicating their excellent barrier properties and corrosion protection capability.

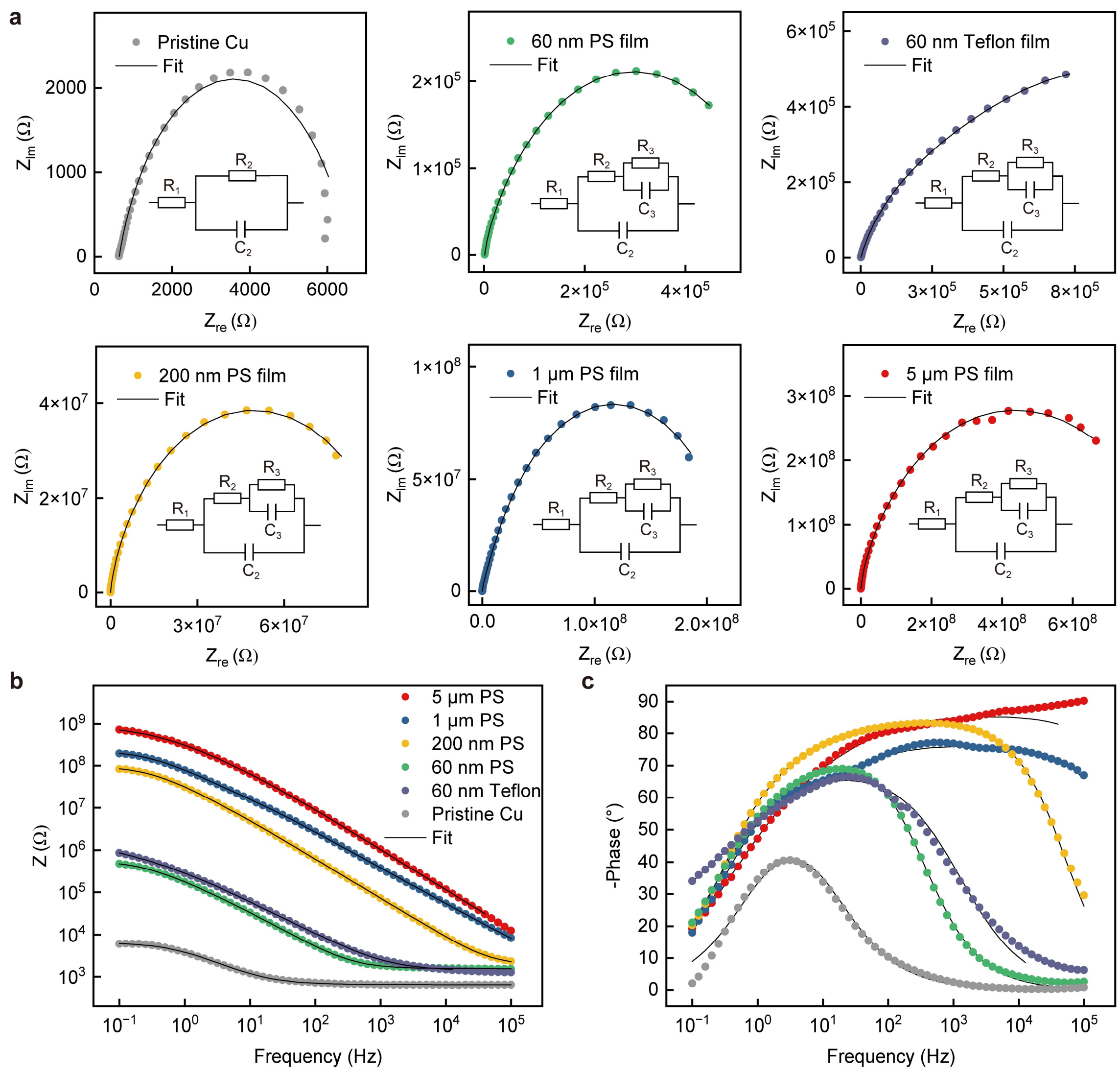

Fig. S27. Electrochemical impedance spectroscopy of sputtered Cu samples with coatings. EIS was performed using a potentiostat (Autolab PGSTAT204) in a three-electrode configuration. The electrolyte was 600 mM aqueous NaCl. **(a)** Nyquist plots of pristine Cu, PS films with different thicknesses coated on Cu, and a 60 nm Teflon film coated on Cu. The pristine bare Cu exhibits the smallest impedance arc. After coating, the arc increases markedly. And the arc further increases with film thickness. The insets show the equivalent circuit models used to fit the EIS data. R_1: solution resistance, R_2: charge transfer resistance, R_3: coating resistance, C_2 and C_3 represent the double layer capacitance and coating capacitance, n: exponent of the C. all the C elements are constant phase elements (CPEs). **(b)** Bode modulus plots. At frequency of 10^{-1}, the coated samples exhibit significantly higher impedance than pristine Cu. **(c)** Bode phase angle plots. Compared with pristine Cu, the coated samples show higher and broader phase angle peaks. The black lines in all figure represent the corresponding fitting results.

Table S2. Fitting parameters for Fig. S27.

Samples	R_2 (Ω)	C_2 ($Ω^{-1}·s^n$)	n_2	R_3 (Ω)	C_3 ($Ω^{-1}·s^n$)	n_3
**Pristine Cu**	$5.9×10^3$	$4.8×10^{-5}$	0.79			
**60 nm PS**	$2×10^5$	$7.9×10^{-7}$	0.87	$4×10^5$	$8.9×10^{-7}$	0.69
**60 nm Teflon**	$7.3×10^5$	$6.6×10^{-7}$	0.78	$9.9×10^5$	$1.6×10^{-6}$	0.7
**200 nm PS**	$2.5×10^7$	$4×10^{-9}$	0.92	$7.6×10^7$	$4×10^{-9}$	0.67
**1 µm PS**	$4.1×10^7$	$1.5×10^{-9}$	0.85	$1.9×10^8$	$1.4×10^{-9}$	0.75
**5 µm PS**	$5.9×10^7$	$2.3×10^{-10}$	0.95	$9×10^8$	$6.7×10^{-10}$	0.56

a

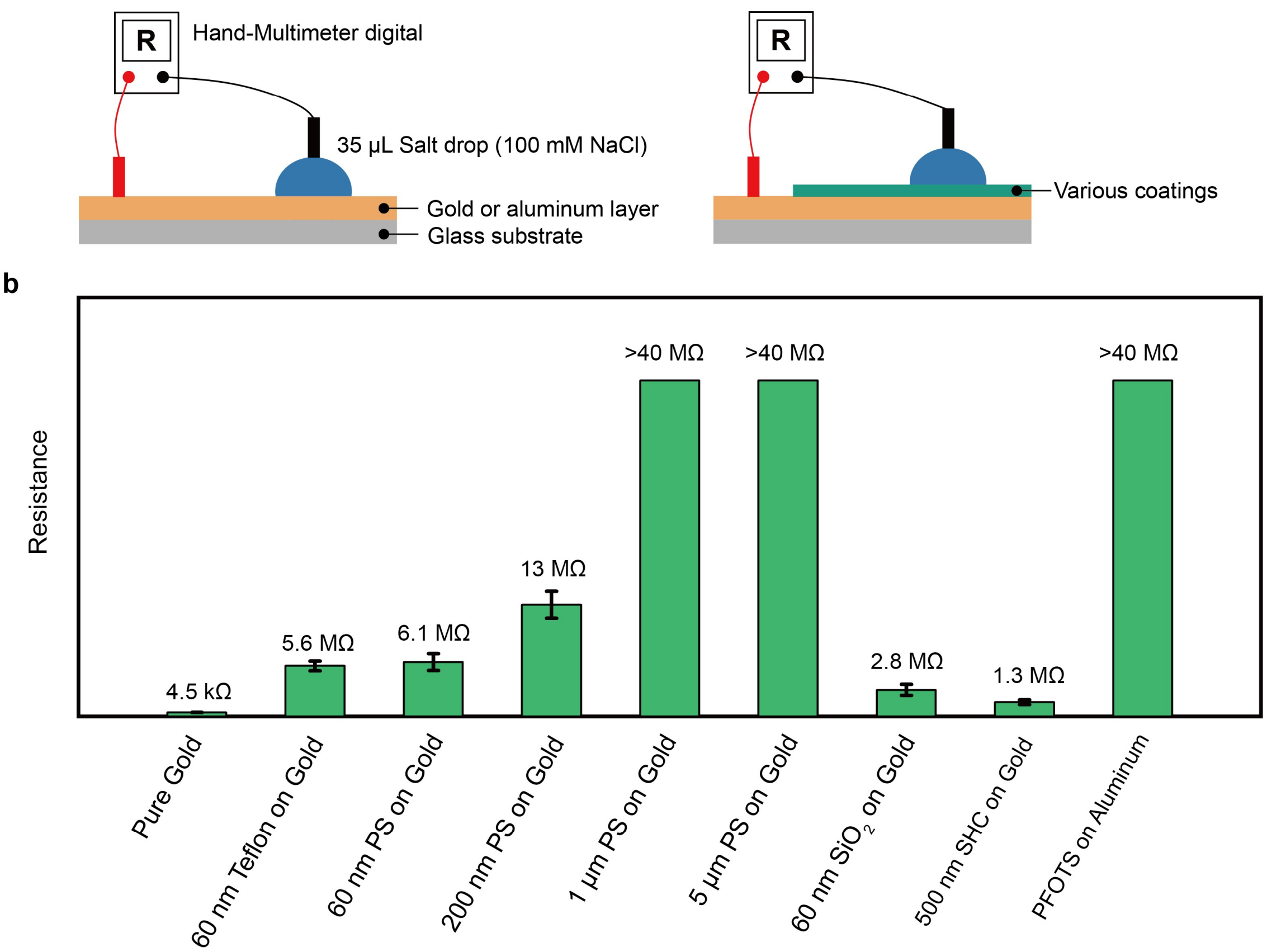

Fig. S28. Electrical properties of coatings. (a) Diagram of measurement method. A hand-Multimeter digital (Extech EX330, range of resistance: 0.1 Ω – 40 MΩ) was used to measure the resistance between an aqueous drop (35 µL 100 mM NaCl) and metal substrate. One electrode is connected directly to the metal layer, while the other is connected to the stationary drop. As shown on the right, an example without any coatings on gold is taken as reference; the remaining resistance of 4.5 kΩ is attributed to drop. Then, various coatings were prepared on the gold layer. The drop was placed on top to measure the resistance between the drop and the underlying gold layer. **(b)** Resistance measurement results. The resistance value for the reference is 4.5 kΩ. For 60 nm Teflon film, 60 nm PS film, 200 nm PS film, 60 nm SiO_2 layer, 500 nm Superhydrophobic coating (SHC), the resistance results are 5.6 MΩ, 6.1 MΩ, 13 MΩ, 2.8 MΩ, 1.3 MΩ respectively. For 1 µm and 5 µm PS film, its resistance exceeds the instrument's measurement range, i.e., greater than 40 MΩ. In particular, for PFOTS (monolayer) coated on aluminum, its resistance also greater than 40 MΩ. The results are the average and standard deviation of measurements taken at 5 different locations.

Reference:

1. Kear, G., Barker, B. D. & Walsh, F. C. Electrochemical corrosion of unalloyed copper in chloride media—a critical review. *Corrosion Science* **46**, 109-135 (2004). doi.org/10.1016/S0010-938X(02)00257-3
2. Wong, W. S. Y. et al. Tuning the Charge of Sliding Water Drops. *Langmuir* **38**, 6224-6230 (2022). doi.org/10.1021/acs.langmuir.2c00941
3. Chen, B. et al. Water–solid contact electrification causes hydrogen peroxide production from hydroxyl radical recombination in sprayed microdroplets. *Proceedings of the National Academy of Sciences* **119**, e2209056119 (2022). doi.org/10.1073/pnas.2209056119
4. Heindel, J. P., LaCour, R. A. & Head-Gordon, T. The role of charge in microdroplet redox chemistry. *Nature Communications* **15**, 3670 (2024). doi.org/10.1038/s41467-024-47879-0
5. Shen, Y. et al. Unveiling the Role of Seawater Microdroplets in Accelerating Steel Corrosion. *Journal of the American Chemical Society* (2026). doi.org/10.1021/jacs.5c20003
6. Rozenak, P. Hemispherical bubbles growth on electrochemically charged aluminum with hydrogen. *International Journal of Hydrogen Energy* **32**, 2816-2823 (2007). doi.org/10.1016/j.ijhydene.2007.01.009
7. Xie, D.-G. *et al.* In situ study of the initiation of hydrogen bubbles at the aluminium metal/oxide interface. *Nature Materials* **14**, 899-903 (2015). doi.org/10.1038/nmat4336
